# TMT proteomics analysis reveals the mechanism of bleomycin-induced pulmonary fibrosis and effects of Ginseng honeysuckle superfine powdered tea

**DOI:** 10.1186/s13020-023-00769-x

**Published:** 2023-05-24

**Authors:** Xiaoli Li, Xin Yu, Yuan Gao, Wenqian Zhao, Yajuan Wang, Fei Yu, Chunli Fu, Haiqing Gao, Mei Cheng, Baoying Li

**Affiliations:** 1 Department of Pharmacy, Qilu Hospital of Shandong University, Jinan, 250012 China; 2 Department of Geriatric Medicine, Qilu Hospital of Shandong University, Jinan, 250012 China; 3 Key Laboratory of Cardiovascular Proteomics of Shandong Province, Qilu Hospital of Shandong University, 107 Wenhuaxi Road, Jinan, 250012 Shandong People’s Republic of China; 4 Jinan Clinical Research Center for Geriatric Medicine, 202132001, Jinan, 250012 China; 5 Jinan Aixinzhuoer Medical Laboratory, Jinan, 250100 China

**Keywords:** Ginseng honeysuckle superfine powdered tea, Pulmonary fibrosis, Proteomics, Network pharmacology, Serum pharmacochemistry

## Abstract

**Background:**

Pulmonary fibrosis (PF) is a chronic and potentially fatal lung disease and disorder. Although the active ingredients of ginseng honeysuckle superfine powdered tea (GHSPT) have been proven to have anti-inflammatory and antioxidant effects, the mechanism of GHSPT on PF remains unclear. The present study was to explore the underlying mechanism of GHSPT in treating PF based on proteomics and network pharmacology analysis and to confirm it in vivo.

**Materials and methods:**

We used intratracheal instillation of bleomycin to induce the PF mouse model and GHSPT (640 mg/kg) intragastrically administrated to PF mice for 21 days. The lung tissues were harvested for TMT-based proteomics. The UPLC-Q-Exactive MS/MS analyze the serum migrant compounds of GHSPT in the PF mice. Moreover, components of GHSPT were harvested from the pharmacology database of the TCMSP system. PF-related targets were retrieved using NCBI and GeneCards databases.

**Results:**

Our results showed that GHSPT significantly alleviated PF mice. Proteomics analysis showed that 525 proteins had significantly changed in the lung of untreated PF mice. Among them, 19 differential proteins were back-regulated to normal levels after GHSPT therapy. Moreover, 25 compounds originating from GHSPT were identified in the serum sample. Network analysis showed 159 active ingredients and 92 drug targets against PF. The signaling pathways include apoptosis, ferroptosis, cytokine-cytokine receptor, P53, and PI3K-Akt signaling pathway.

**Conclusion:**

The evidence suggests that GHSPT might play an effective role in the treatment of PF by multi-target interventions against multiple signaling pathways.

**Supplementary Information:**

The online version contains supplementary material available at 10.1186/s13020-023-00769-x.

## Introduction

Pulmonary fibrosis (PF) is a diffuse inflammatory disease that is the inevitable progression of many lung diseases at the end stage, resulting in respiratory failure and death with high morbidity and mortality [1]. PF is characterized by abnormal proliferation and apoptosis of fibroblasts and excessive accumulation of extracellular matrix. Many varieties of factors can trigger pulmonary fibrosis, such as viral and bacterial infection, cigarette exposure, and environmental particles [2]. There is growing evidence to support the link between PF, oxidative stress, epithelial-to-mesenchymal transition (EMT), and ferroptosis [3, 4]. However, the molecular mechanisms of PF occurrence have not been fully elucidated. Therefore, it is of great clinical significance to elucidate the possible pathogenesis of PF and to search for effective drug targets for treatment.

In order to develop the cure for PF, scientists from various countries have carried out a lot of exploration. Until now, there are very few drugs that can delay the onset of pulmonary fibrosis internationally, with modest benefits and considerable side effects. There is still a lack of effective treatment for PF. Therefore, the development of new drugs for the treatment of pulmonary fibrosis has important practical significance to improve the quality of life and prolong the survival time of patients.

Botanical drug can exert anti-inflammatory, antioxidant effects, which may play crucial roles in treating of various diseases, including infectious diseases, cardiovascular diseases and cancer. Some researchers found that the treatment of integrated traditional Chinese and Western medicine had better effects, effectively reduced the severity of PF without increasing adverse drug reactions. From ancient times to the present, people have realized that medicine and food have the same origin. Studies have shown that botanical drug can protect against various lung diseases [5, 6]. Ginseng honeysuckle superfine powdered tea (GHSPT) includes the *Panax ginseng C.A. Mey*, *Lonicera japonica Thunb*, *Wurfbainia villosa var. villosa, Citrus × aurantium f. deliciosa*, *Poria cocos*, *Glycyrrhiza uralensis Fisch. ex DC*, *Gardenia jasminoides J. Ellis*, and *Camellia sinensis L. Kuntze*. Many researchers have reported that ginseng, honeysuckle, and licorice all play an important role against PF [7, 8]. Experimental studies have shown that ginseng and licorice have anti-inflammatory, anti-oxidation, antitussive and expectorant activities. For hundreds of years, tea has been used as a health drink and an important source of biological activity, most of which are polyphenols. Tea polyphenols have anti-inflammatory, anti-tumorigenic, antioxidant, anti-arteriosclerosis, and anti-proliferative properties [9]. However, the molecular mechanism of GHSPT in the treatment of PF remains unclear.

With the development of multiomics technology, many diseases have been proven to be caused by a combination of many factors. Therefore, it has become a trend to treat diseases from multicomponent, multitarget, and multipathway, involving the use of a variety of drugs. Botanical drug has synergistic regulation by multicomponent, multitarget, and multipathway in the treatment of diseases. Proteomics and network pharmacology are powerful tools for the comprehensive analysis of drug targets [10, 11]. In this work, we adopted proteomics and network pharmacology to investigate the molecular mechanism of GHSPT for PF treatment.

## Materials and methods

### Materials

GHSPT (Batch No: 20210512) was produced by China Tea (Hunan) Co., Ltd (Changsha, China). The preparation of GHSPT is ultrafine powder. Then we made a decoction of GHSPT with a concentration of 64 mg/mL. Detailed information of GHSPT was described in Additional file 4. TMT 10 plex kit was purchased from Thermo Scientific (Carlsbad, USA). Bleomycin hydrochloride was purchased from MedChem Express (New Jersey, USA). The antibodies of E-cadherin, heat shock protein 90 (HSP90), Transcription factor AP-1 (JUN), matrix metalloproteinase 1 (MMP1), P53, protein kinase B (Akt), signal transducer and activator of transcription 3 (STAT3), 1-phosphatidylinositol-3-phosphate 5-kinase (PIKfyve), FMR2 family member 4 (AFF4) and β-actin were all purchased from Proteintech (Wuhan, China). The antibody of angiomotin-like protein 2 (Amotl2) was purchased from BOSTER (Wuhan, China). The antibodies of transforming growth factor-β1 (TGF-β1), p-Akt (Ser473), and p-STAT3 (Ser727) were purchased from ABclonal (Wuhan, China). The antibody of glucagon-like peptide 1 receptor (GLP1R) was purchased from Servicebio (Wuhan, China). The antibody of α-smooth muscle actin (α-SMA) was purchased from Abcam (Cambridge, USA). All other chemical reagents were purchased with analytical grade.

### Animals

Male C57BL/6J mice (n = 50, 7 weeks old) were purchased from SPF Biotechnology Co., Ltd (Beijing, China). All mice were housed in standard cages with temperature of 20–22 °C and humidity of 55 ± 5%, and received laboratory pellet chow and tap water ad libitum. The mice were kept under observation for one week prior to the start of experiment. All procedures were approved by the Animal Ethics Committee of Shandong University (Approval No: 22022). C57BL/6J mice were selected as the control group (CC, n = 10). The other mice received a single dose of bleomycin (2 mg/kg, intratracheal instillation) freshly dissolved in normal saline. The 38 successfully operated mice were divided into 2 groups: an untreated PF group (PF, n = 19) and another PF group treated by GHSPT with a dosage of 640 mg/kg (based on the body surface area conversion index of human and mouse) by intragastric administration for 21 days. At the end of the experiments, all mice were sacrificed under sodium pentobarbital anesthesia. For in vivo component analysis, plasma was collected 2 h after GHSPT intragastric administration. Lungs were dissected. The tissues and sera were kept at −80 °C until further analysis.

### Estimation of body weight, lung weight, survival rate and coefficient

At the end of the experiment, mice and lung were weighed. Lung coefficient was determined by lung weight (mg) versus body weight (g). The survival rate was evaluated.

### Light microscopy

Left lungs were excised and fixed in 4% paraformaldehyde, and embedded in paraffin, and cut into 4 μm-thick sections. Then they were stained with hematoxylin and eosin (HE), Masson’s Trichrome, and immunofluorescence (E-cadherin: 1–200, α-SMA: 1:1000). Ashcroft score was used to evaluate lung fibrosis. The mass intensity (collagen area/total area) of every group was measured by Image J software and was expressed in arbitrary units as a percentage of the total area of tissue section stained.

### TMT-labeled quantitative proteomic analysis

The three lung tissues of each group (CC, PF, and GH) were selected to extract proteins. Protein was extracted using SDT lysis buffer. Samples were boiled for 5 min and further ultrasonicated and boiled again for another 3 min. Undissolved cellular debris was removed by centrifugation at 16,000 g for 20 min. The supernatant was collected and quantified with a BCA Protein Assay Kit (Bio-Rad, USA). The digested samples were labeled with TMT reagent according to the kit protocol. LC-MS/MS was performed on a Q Exactive HF-X mass spectrometer coupled with Easy 1200 nLC (Thermo Fisher Scientific). The full MS scans were acquired at a resolution of 60,000 at m/z 200, and 45,000 at m/z 200 for MS/MS scans. The maximum injection time was set to 50 ms for MS and 50 ms for MS/MS. The normalized collision energy was 32 and the isolation window was set to 1.2 m/z. Dynamic exclusion duration was 60 s.

The MS data were analyzed using the search engine Sequest HT in the Proteome Discoverer software (version 2.4, Thermo Scientific) for database retrieval. MS data were searched against the Uniprot-Mus musculus (Mouse) [10090]-88027-20220607, which is derived from the network address https://www.uniprot.org/taxonomy/10090 protein database, its protein entry: 88,027. The database search results were filtered and exported with < 1% false discovery rate (FDR) at peptide-spectrum-matched level, and protein level, respectively.

### Identification of serum migrant compounds of GHSPT

The six serum samples of in each group (GH and PF) were selected to extract metabolites. 100 µL serum sample was thoroughly mixed with 400 µL of cold methanol acetonitrile (v/v, 1:1) via vortexing. The samples were obtained by ultrasonic 1 h in the ice bath and placed at −20 ℃ for 1 h and centrifuged at 16,000 g and 4 °C for 20 min. The supernatants were then harvested and dried under vacuum UPLC-MS analysis.

Chromatographic separation was completed on the Water UPLC I-class-TripleTOF^®^ 5600 + MS system which was equipped with a Waters UPLC BEH Amide column ( 1.7 μm 2.1 × 100 mm). The injection volume is 5ul, the column temperature is 40 ℃, the flow rate is 300ul/min, the chromatographic mobile phase A: water + 20mM ammonium acetate, B: acetonitrile, the chromatographic gradient elution procedure is as follows: 0–0.1 min, 2% A; 0.1–12 min, A changes linearly from 2 to 25%; 12–13.5 min, A changes linearly from 25 to 60%; 13.5–15 min, A holds at 60%; 15–15.1 min, A changes linearly from 60 to 2%; 15.1–18 min, A holds at 2%. The experiments of ESI-MS were conducted in positive and negative ion modes, respectively. Mass spectrometry parameters were set as follows: spray voltage: 3.8 kv (positive) and 3.2 kv (negative). Capillary temperature 320 ℃; sheath gas (nitrogen) flow: 30 arbitrary units; Aux Gas flow: 5 arb; Probe Heater Temp: 350 ℃; S-Lens RF Level 50. Full mass scan (m/z 60–1200) and MS/MS spectra were recorded at a resolution of 70,000 at m/z 200, and 17,500 at m/z 200.

### Network pharmacology analysis

GHSPT has a total of 8 botanical drug components, and the potential active ingredients and serum migrant compounds were searched in the TCMSP (Traditional Chinese Medicine Systems Pharmacology Database and Analysis Platform, http://tcmspw.com/tcmsp.php). The potential active ingredients in GHPST were screened according to the pharmacokinetic parameters of oral bioavailability (OB) ≥ 30% and drug likeness (DL) ≥ 0.18 screening conditions.

The targets related to the PF pathogenesis were acquired by retrieving GeneCards (http://www.genecards.org) and NCBI (http://www.ncbi.nlm.nih.gov). All collected targets were mixed together, and repeated targets were removed to find the unique project to establish a PF-related target set. With R software (version 4.0.3) and Venn Diagram package, we mapped the potential gene targets (PF), and related targets (potential active ingredients and serum migrant compounds of GHSPT), and found the potential targets of GHPST in treating PF.

The botanical drug—ingredients- target network of GHPST in preventing and treating PF was constructed by the Cytoscape software (version 3.7.1). Among them, “node” represents active ingredients and related targets, and “edge” represents the relationship between them. Network topology analysis was carried out for the obtained action network, and the degree value of the node was used to reflect the importance of the “node”. The degree value was positively correlated with the importance of “node”.

### Bioinformatic analysis

The global protein changes data in the lung were analyzed with the Perseus software, Microsoft Excel, and R statistical computing software. Proteins whose abundance was down-regulated more than 0.83-fold, and up-regulated less than 1.20-fold were ignored to increase statistical sensitivity. The potential targets of GHSPT and the differently expressed proteins of lung tissues were analyzed. To annotate the sequences, information was extracted from the Swiss-Prot, Gene Ontology (GO), and Kyoto Encyclopedia of Genes and Genomes (KEGG). GO and KEGG enrichment analyses were carried out with Fisher’s exact test, and FDR correction for multiple testing was also performed. GO terms were grouped into three categories: cell component (CC), biological process (BP), and molecular function (MF). Construction of protein-protein interaction (PPI) networks was also conducted by using the STRING database with the cytoscape software.

### Western blot analysis

The lung samples were homogenized in ice-cold lysis buffer containing PMSF (Beyotime Biotechnology, Jiangsu, China). An equal amount of protein was separated by SDS-PAGE (10%) and transferred onto polyvinylidene difluoride membranes. The membrane was sealed with PBST-5% skimmed milk or PBST-5% BSA, and then incubated overnight with the antibody at 4 °C as follows: E-cadherin (1:5000), α-SMA (1:1000), PIKfyve (1:500), AFF4 (1:2000), Amotl2 (1:1000), and GLP1R (1:1000), HSP90AA1 (1:2000), JUN (1:2000), MMP1 (1:1000), TGF-β1 (1:1000), P53 (1:1000), Akt (1:1000), pho-Akt (Ser473, 1:1000), STAT3 (1:2000), pho-STAT3 (Ser727, 1:1000). Secondary antibody (Beyotime, China) was applied for 1 h at room temperature. The intensity of immunoblot bands was normalized to that of β-actin (1:2000). Densitometry was obtained for quantification of each identified protein band and analyzed with Image J densitometry software.

### Statistical analysis

Data were expressed as mean ± standard deviation. Statistical analysis between groups was made using one-way analysis of variance (ANOVA) and Student’s t-test for comparisons. *P*-value < 0.05 was considered statistically significant. All analyses were performed with SPSS for Windows software version 22.0 (SPSS, Chicago, USA).

## Results

### Effects of GHSPT on body weight, survival rate, lung weight and coefficient, and histological findings

**Fig. 1:**
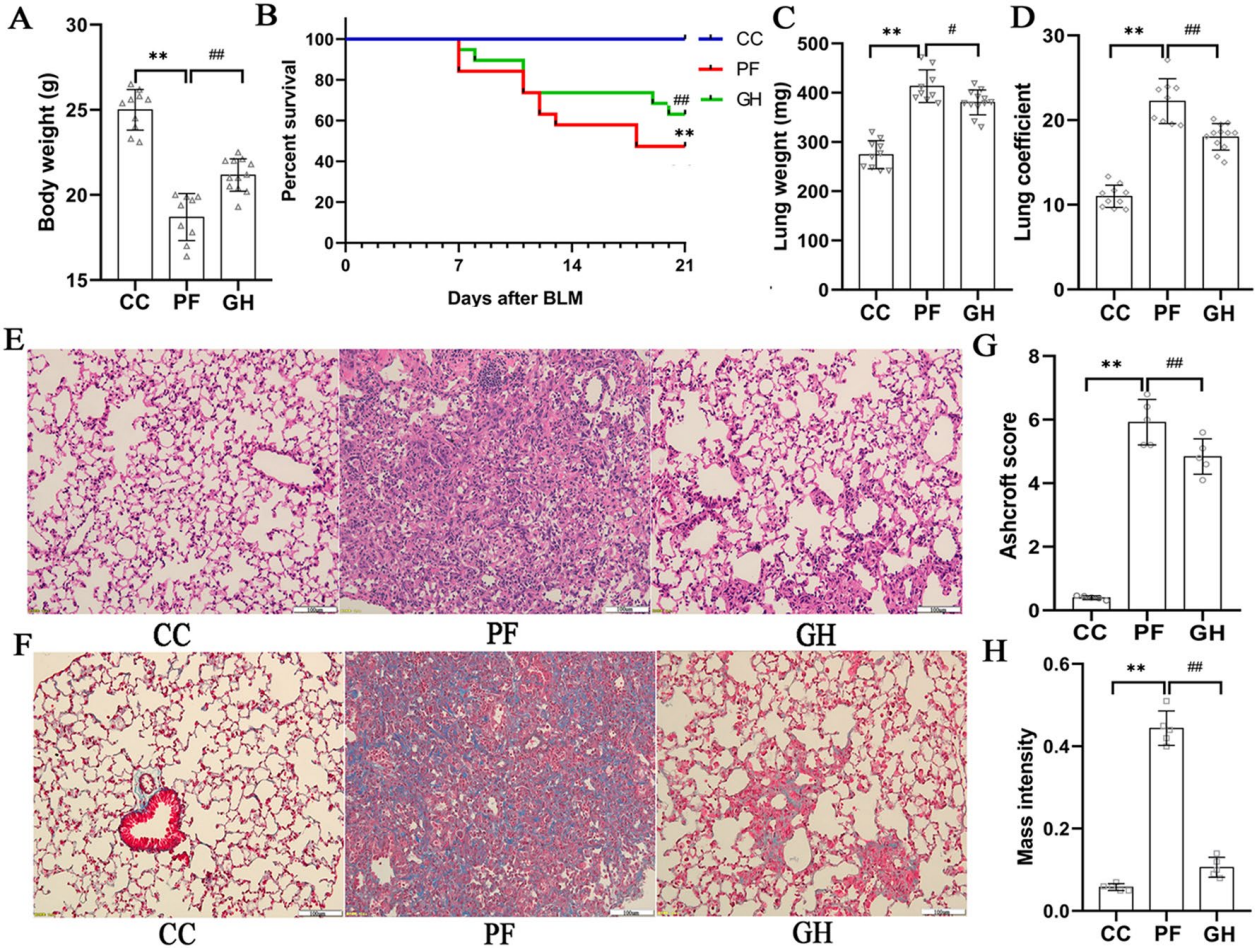
Effects of GHSPT on body weight, survival rate, lung weight and coefficient, and histological findings of the lung in bleomycin-induced PF mice. **A** Body weight changes of the mice at 11 weeks old. **B** Survival rate changes of the mice from 8 to 11 weeks old. **C** Lung weight changes of the mice at 11weeks old. **D** Lung coefficient changes of the mice at 11-week-old. **E** Representative light micrographs of the lung tissue (Hematoxylin Eosin; bar: 100 μm). **F** Representative light micrographs of the lung tissue (Masson’s Trichrome; bar: 100 μm). **G** Ashcroft score changes of lung histopathology in the mice. **H** Mass intensity (collagen area/total area) of pulmonary fibrosis in the mice. ^*^P < 0.05, ^**^P < 0.01 compared with CC group; ^#^*P* < 0.05, ^##^*P* < 0.01 compared with PF group. CC: control group; PF: bleomycin-induced PF group; GH: GHSPT treated bleomycin-induced PF group. GHSPT: ginseng honeysuckle superfine powdered tea; PF: pulmonary fibrosis

The number that remained alive at the end of the research in the three groups was 10, 9, and 12 respectively of group CC, PF, and GH. The body weight and survival rate of mice showed a significant decrease in the PF group (*P* < 0.01). GHSPT treatment group significantly increased body weight and survival rate at 21 days (*P* < 0.01, Fig.1A and B). At the end of the experiment, the lung weight and lung coefficient of the PF group were significantly higher than that in the CC group, while those in the GH group were decreased (*P* < 0.05, Fig. 1C and D).

Under light microscopy, the lung fibrosis and collagen content were significantly higher observed in the PF group than those of the CC group. A decreased level of fibrotic formation was observed in the lung of the GH group when treated with GHSPT (Fig. 1E and F). Moreover, the ashcroft score and mass intensity showed a significant increase in the PF group (*P* < 0.01). GHSPT treatment group significantly decreased the ashcroft score and mass intensity (*P* < 0.01, Fig. 1G and H).

### Effects of GHSPT on the expression of E-cadherin and α-SMA

**Fig. 2:**
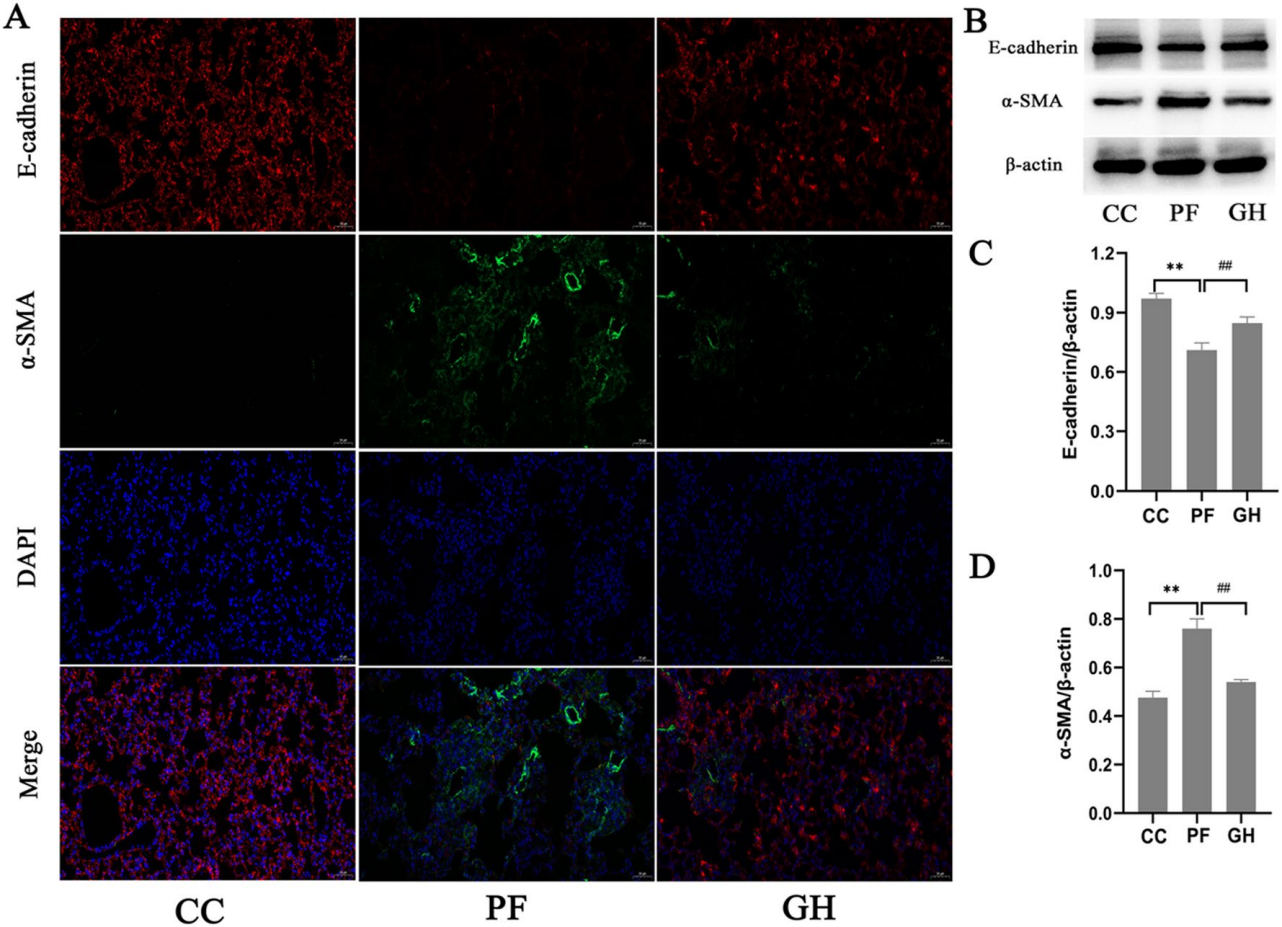
Effects of GHSPT on the expression of E-cadherin and α-SMA of the lung in bleomycin-induced PF mice. **A** Immunofluorescence images of E-cadherin and α-SMA in the lung tissue (×200). **B** Western blot images of E-cadherin and α-SMA in the lung tissue. **C**, **D** Data were expressed as the expression ratio of E-cadherin/β-actin and α-SMA/β-actin. ^*^P < 0.05, ^**^P < 0.01 compared with CC group; ^#^*P* < 0.05, ^##^*P* < 0.01 compared with PF group. CC: control group; PF: bleomycin-induced PF group; GH: GHSPT treated bleomycin-induced PF group. GHSPT: ginseng honeysuckle superfine powdered tea; PF: pulmonary fibrosis

GHSPT affects pulmonary EMT in BLM-induced PF, we investigated the expression of E-cadherin and α-SMA in the lung tissues of mice. By immunofluorescence, E-cadherin positive areas were decreased in the lung tissues of mice, and GHSPT nearly completely restored the E-cadherin-positive area in the lung tissues. By contrast, α-SMA-positive areas were considerably increased in the lung tissues of the PF group, while GHSPT significantly decreased the α-SMA-positive areas (Fig. 2A). To further investigate the effect of GHSPT on EMT, we measured the protein expression of E-cadherin and α-SMA by western blot. Consistently, GHSPT increased the protein expression of E-cadherin, and inhibited the protein expression of α-SMA in PF group (Fig. 2B, C and D, P < 0.01).

### Protein identification and potential targets of GHSPT in treating PF

**Fig. 3:**
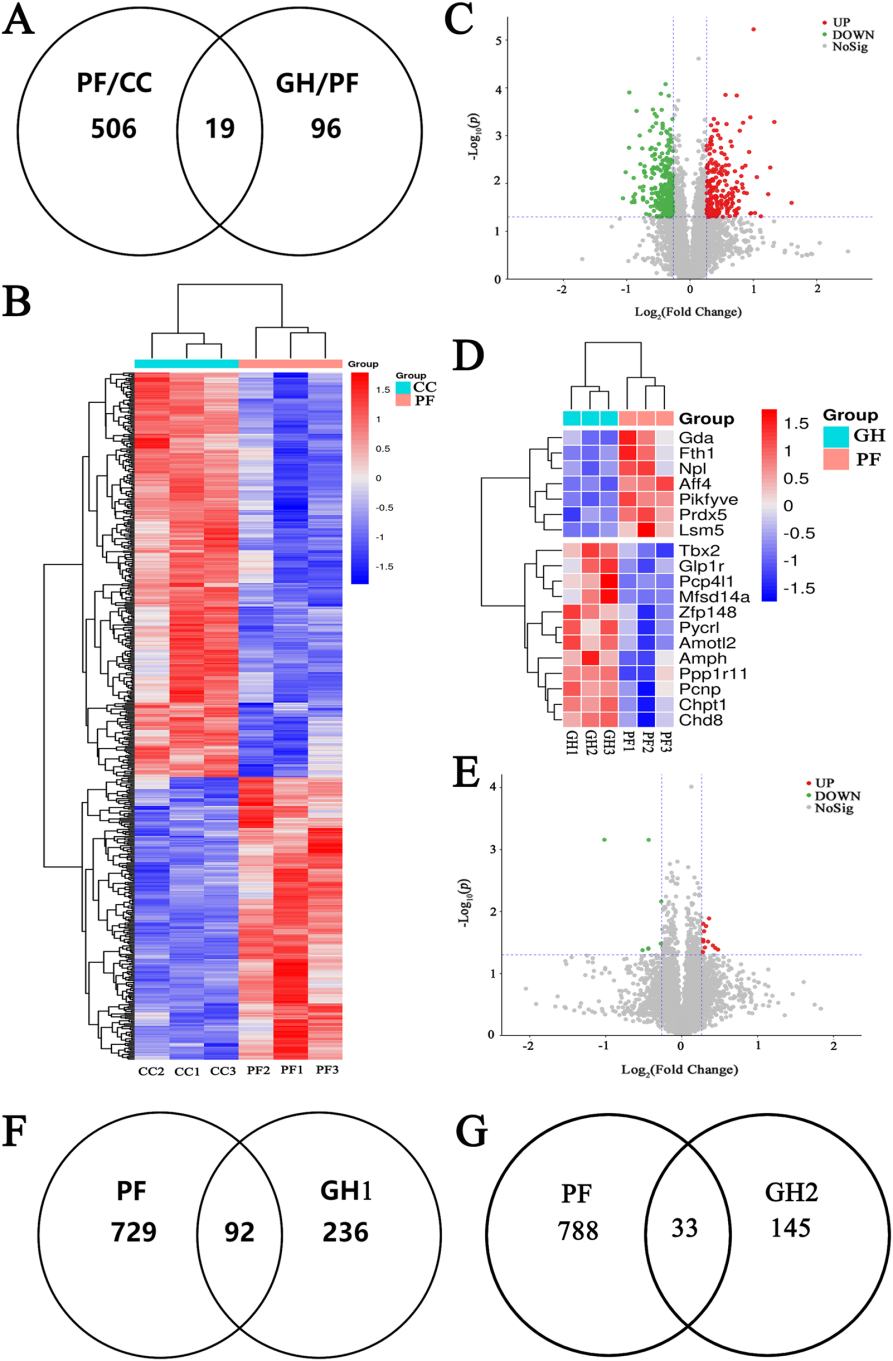
Protein identification and potential targets of GHSPT in treating PF. **A** Proteomics analysis of 19 back-regulated proteins after GHSPT therapy in the lung tissue of PF mice. Volcano plot indicating significantly altered proteins identified in the combined datasets, **B** between PF group and CC group; **C** between GH group and PF group. Cluster heatmap indicating significantly altered proteins identified in the combined datasets, **D** between PF group and CC group; **E** between GH group and PF group. **F** A total of 821 and 328 targets of PF and potential active ingredients (GHSPT), respectively, and they shared 92 overlapping targets. **G** A total of 821 and 178 targets of PF and serum migrant compounds (GHSPT), respectively, and they shared 33 overlapping targets. *CC* control group, *PF* bleomycin-induced PF group, *GH* GHSPT treated bleomycin-induced PF group, *GH1* potential active ingredients of GHSPT, *GH2* serum migrant compounds of GHSPT, *GHSPT* ginseng honeysuckle superfine powdered tea, *PF* pulmonary fibrosis

Proteomics analysis showed that 525 proteins had significantly changed in the lung of untreated PF mice (Additional file 4: Table S1). Among them, 19 differential proteins were back-regulated to normal levels after GHSPT therapy (Fig. 3A, and Table 1). These results demonstrate that bleomycin-induced mice have significant effects on the lung proteins, but that these effects are reversed by GHSPT treatment in PF mice. The back-regulated proteins include peroxiredoxin-5, AFF4, PIKfyve, E3 ubiquitin-protein ligase PPP1R11, Amotl2 and GLP1R. Volcano plot and cluster heatmap indicating significantly altered proteins identified in the combined datasets. Proteins plotted against log-transformed fold change in abundance between the PF group and CC group (Fig. 3B and C), and between the GH group and PF group (Fig. 3D and E).

**Table 1:** Identified significant differenced expression proteins of lung in the pulmonary fibrosis mice as reversed by Ginseng honeysuckle superfine powdered tea

Accession	Gene symbol	Protein name	Molecular weight (kDa)	Expression ratio (PF/CC)	Expression ratio (GH/PF)
P99029	Prdx5	Peroxiredoxin-5, mitochondrial	21.9	1.28	0.83
P09528	Fth1	Ferritin heavy chain	21.1	1.41	0.83
Q71RH5	Gda	Guanine deaminase	51.0	1.60	0.70
Q9DCJ9	Npl	N-acetylneuraminate lyase	35.1	1.51	0.74
Q9ESC8	Aff4	AF4,FMR2 family member 4	126.6	2.52	0.49
H3BKC5	Lsm5	U6 snRNA-associated Sm-like protein LSm5 (Fragment)	4.6	1.53	0.74
D3Z5N5	Pikfyve	1-Phosphatidylinositol-3-phosphate 5-kinase	231.9	1.21	0.74
Q6P8I4	Pcnp	PEST proteolytic signal-containing nuclear protein	19.0	0.75	1.21
A0A668KM31	Amph	Amphiphysin	89.8	0.79	1.36
Q61624	Zfp148	Zinc finger protein 148	88.7	0.74	1.23
Q6W8Q3	Pcp4l1	Purkinje cell protein 4-like protein 1	7.5	0.51	1.27
Q9DCC4	Pycrl	Pyrroline-5-carboxylate reductase 3	28.7	0.81	1.22
D3YU39	Chpt1	Cholinephosphotransferase 1	45.4	0.72	1.25
A5A4Z0	Ppp1r11	E3 ubiquitin-protein ligase PPP1R11	14.1	0.68	1.33
Q60707	Tbx2	T-box transcription factor TBX2	75.0	0.78	1.22
O35659	Glp1r	Glucagon-like peptide 1 receptor	53.0	0.60	1.39
P70187	Mfsd14a	Hippocampus abundant transcript 1 protein	53.0	0.79	1.24
Q3TP05	Amotl2	Angiomotin-like protein 2	80.1	0.77	1.28
Q09XV5	Chd8	Chromodomain-helicase-DNA-binding protein 8	290.7	0.67	1.22

We obtained 17 active ingredients in Panax ginseng C.A. Mey, 17 in Lonicera japonica Thunb, 8 in Wurfbainia villosa var. villosa, 5 in Citrus × aurantium f. deliciosa, 6 in Poria cocos, 88 in Glycyrrhiza uralensis Fisch. ex DC, 12 in Gardenia jasminoides J.Ellis, and 6 in Camellia sinensis (L.) Kuntze (Additional file 4: Table S2). We obtained 92 potential therapeutic targets of GHSPT (between potential active ingredients and PF), and 33 related targets of GHSPT (between serum migrant compounds and PF) (Fig. 3F and G, Additional file 4: Table S3,S4 and S5).

### Identification of serum migrant compounds and botanical drugs‑active ingredients-drug targets network analysis of GHSPT

**Fig. 4:**
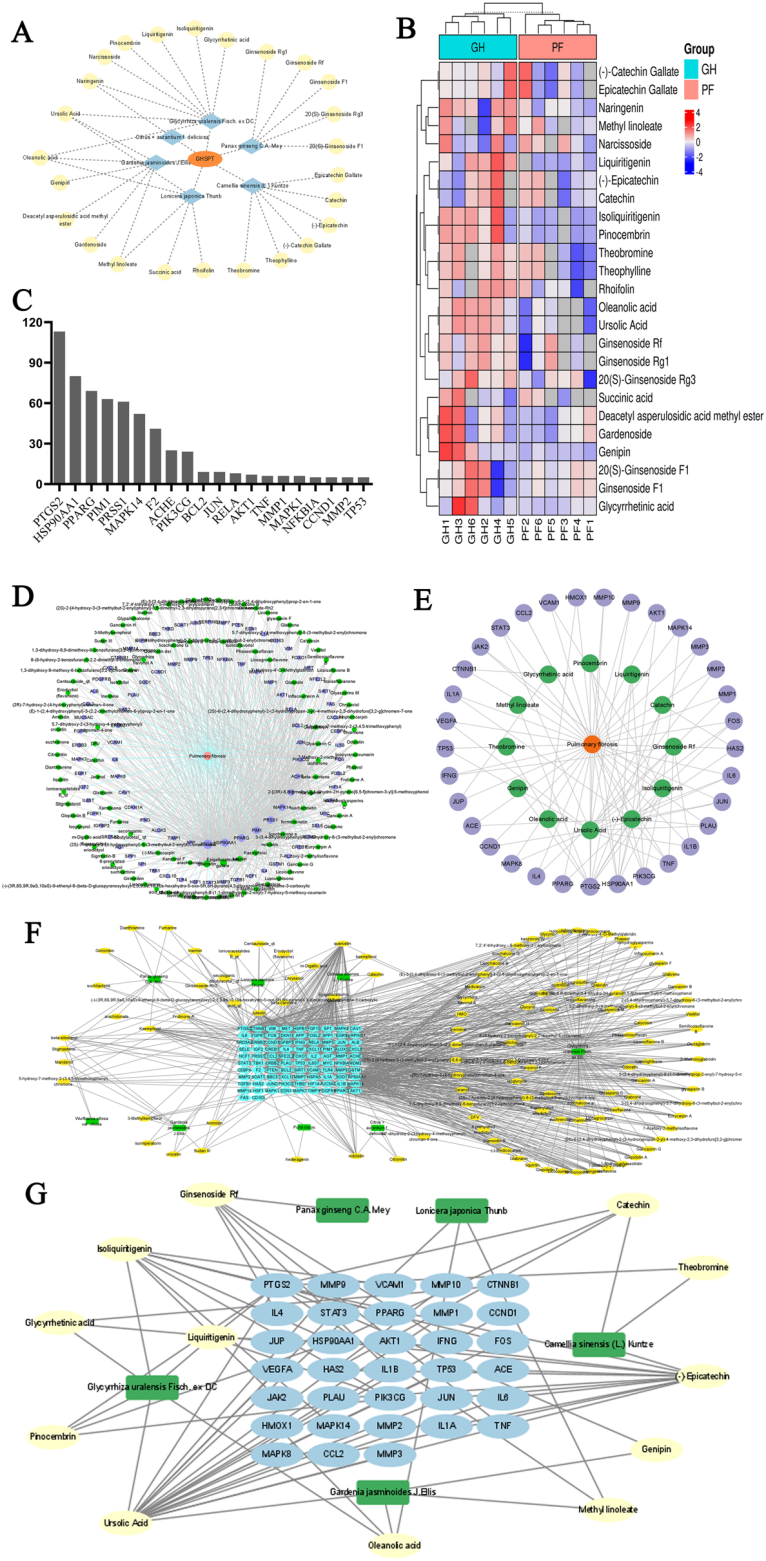
Identification of serum migrant compounds and botanical drugs‑active ingredients-drug targets network analysis of GHSPT. **A** GHSPT- component- serum migrant compounds network. **B** Cluster heatmap of serum migrant compounds of GHSPT. **C** Top 20 potential targets and related active ingredients number. GHSPT: ginseng honeysuckle superfine powdered tea. **D** Disease‑ potential active ingredients-target network of GHSPT. **E** Disease‑ serum migrant compounds-target network of GHSPT. **F** Botanical drugs‑potential active ingredients-drug targets network of GHSPT. **G** Botanical drugs‑serum migrant compounds-drug targets network of GHSPT

The overall distribution trend of all samples was observed through PCA analysis is shown in Additional file 4: Fig. S1. Based on the basis of standards and related literatures, 25 compounds from GHSPT in total were identified initially (Table 2). These compounds involved prenol lipids, flavonoids, isoflavonoids, iridoid glycoside, tetracyclic triterpenoids, organooxygen compounds, glycerolipids, linear 1, 3-diarylpropanoids, fatty acyls, carboxylic acids, and derivatives and imidazopyrimidines. According to previous research, above compounds might originate from *Panax ginseng C.A. Mey, Lonicera japonica Thunb, Citrus × aurantium f. deliciosa, Poria cocos, Glycyrrhiza uralensis Fisch. ex DC, Gardenia jasminoides J.Ellis* and *Camellia sinensis (L.) Kuntze* in GHSPT. The GHSPT-component- serum migrant compounds network was developed (Fig. 4A). The cluster heatmap of serum migrant compounds of GHSPT was shown in Fig. 4B.

**Table 2:** Characterization of serum bioactive ingredients of Ginseng honeysuckle superfine powdered tea by UPLC-Q-Exactive MS/MS

No.	Ingredient	Formula	RT (min)	Precursor ion	Fragment ions (m,z)	Class	Source
1	Glycyrrhetinic acid	C_3__0_H_46_O_4_	1.39	[M + H]+	471.3469	Prenol lipids	GU
2	Genipin	C_11_H_14_O_5_	9.79	[M + H]+	227.0914	Prenol lipids	GJ
3	Liquiritigenin	C_15_H_12_O_4_	11.81	[M-H]−	255.0663	Flavonoids	GU
4	Oleanolic acid	C_30_H_48_O_3_	1.40	[M + H]+	457.3676	Prenol lipids	GU, LJ, GJ
5	Ursolic acid	C_30_H_48_O_3_	1.40	[M + H]+	457.3676	Prenol lipids	GU, LJ, GJ
6	Naringenin	C_15_H_12_O_5_	1.37	[M-H]−	271.0612	Flavonoids	GU, CA
7	Deacetyl asperulosidic acid methyl ester	C_17_H_24_O_11_. NH_3_	5.25	[M-H]−	420.1511	Iridoid glycoside	GJ
8	Gardenoside	C_17_H_24_O_11_. NH_3_	5.25	[M-H]−	420.1511	Prenol lipids	GJ
9	20(S)-Ginsenoside F1	C_36_H_62_O_9_. Na	4.55	[M-H]−	660.4219	Tetracyclic triterpenoids	PG
10	Ginsenoside F1	C_36_H_62_O_9_. Na	4.55	[M-H]−	660.4219	Prenol lipids	PG
11	20(S)-Ginsenoside Rg3	C_42_H_72_O_13_N	12.29	[M-H]−	797.4931	Tetracyclic triterpenoids	PG
12	Ginsenoside Rf	C_42_H_72_O_14_. HCOOH	1.24	[M + H]+	847.505	Glycerolipids	PG
13	Ginsenoside Rg1	C_42_H_72_O_14_. HCOOH	1.24	[M + H]+	847.505	Prenol lipids	PG
14	Isoliquiritigenin	C_15_H_12_O_4_	11.81	[M-H]−	255.0663	Linear 1, 3- diarylpropanoids	GU
15	Pinocembrin	C_15_H_12_O_4_	11.81	[M-H]−	255.0663	Flavonoids	GU
16	Rhoifolin	C_27_H_30_O_14_	10.63	[M-H]−	577.1563	Flavonoids	LJ
17	Succinic acid	C_4_H_6_O_4_	13.58	[M-H]−	117.0193	Carboxylic acids and derivatives	LJ
18	Methyl linoleate	C_19_H_34_O_2_	1.37	[M-H]−	293.2486	Fatty acyls	LJ, GJ
19	Theobromine	C_7_H_8_N_4_O_2_	10.66	[M-H]−	179.0575	Imidazopyrimidines	CS
20	Theophylline	C_7_H_8_N_4_O_2_	10.66	[M-H]−	179.0575	Imidazopyrimidines	CS
21	(−)-Epicatechin	C_15_H_14_O_6_	13.32	[M-H]−	289.0718	Flavonoids	CS
22	Catechin	C_15_H_14_O_6_	13.32	[M-H]−	289.0718	Flavonoids	CS
23	Narcissoside	C_28_H_32_O_16_	12.69	[M-H]−	623.1617	Flavonoid glycoside	GU
24	(−)-Catechin gallate	C_22_H_18_O_10_	10.41	[M + H]+	443.0973	Flavonoids	CS
25	Epicatechin gallate	C_22_H_18_O_10_	10.41	[M + H]+	443.0973	Flavonoids	CS

*CA* *Citrus  aurantium f. deliciosa*, *CS* *Camellia sinensis* (L.) Kuntze, *GJ* *Gardenia jasminoides J.Ellis*, *GU* *Glycyrrhiza uralensis Fisch.* ex DC, *LJ Lonicera japonica Thunb*, *PG Panax ginseng C.A. Mey*

The top 20 potential targets include prostaglandin-endoperoxide synthase 2 (PTGS2), heat shock protein 90 alpha family class A member 1 (HSP90AA1), peroxisome proliferator-activated receptor gamma, proto-oncogene serine/threonine-protein kinase Pim-1, trypsin-1, mitogen-activated protein kinase 14, prothrombin, acetylcholinesterase, phosphatidylinositol-4, 5-bisphosphate 3-kinase catalytic subunit gamma isoform. PTGS2 and HSP90AA1 can interact with up to 113 and 80 related active ingredients (Fig. 4C). Disease‑potential active ingredients and serum migrant compounds-target networks were developed, involving 92 targets and 33 targets (Fig. 4D and E). Botanical drug‑potential active ingredients and serum migrant compounds-target networks were developed (Fig. 4F and G).

### GO function enrichment analysis

**Fig. 5:**
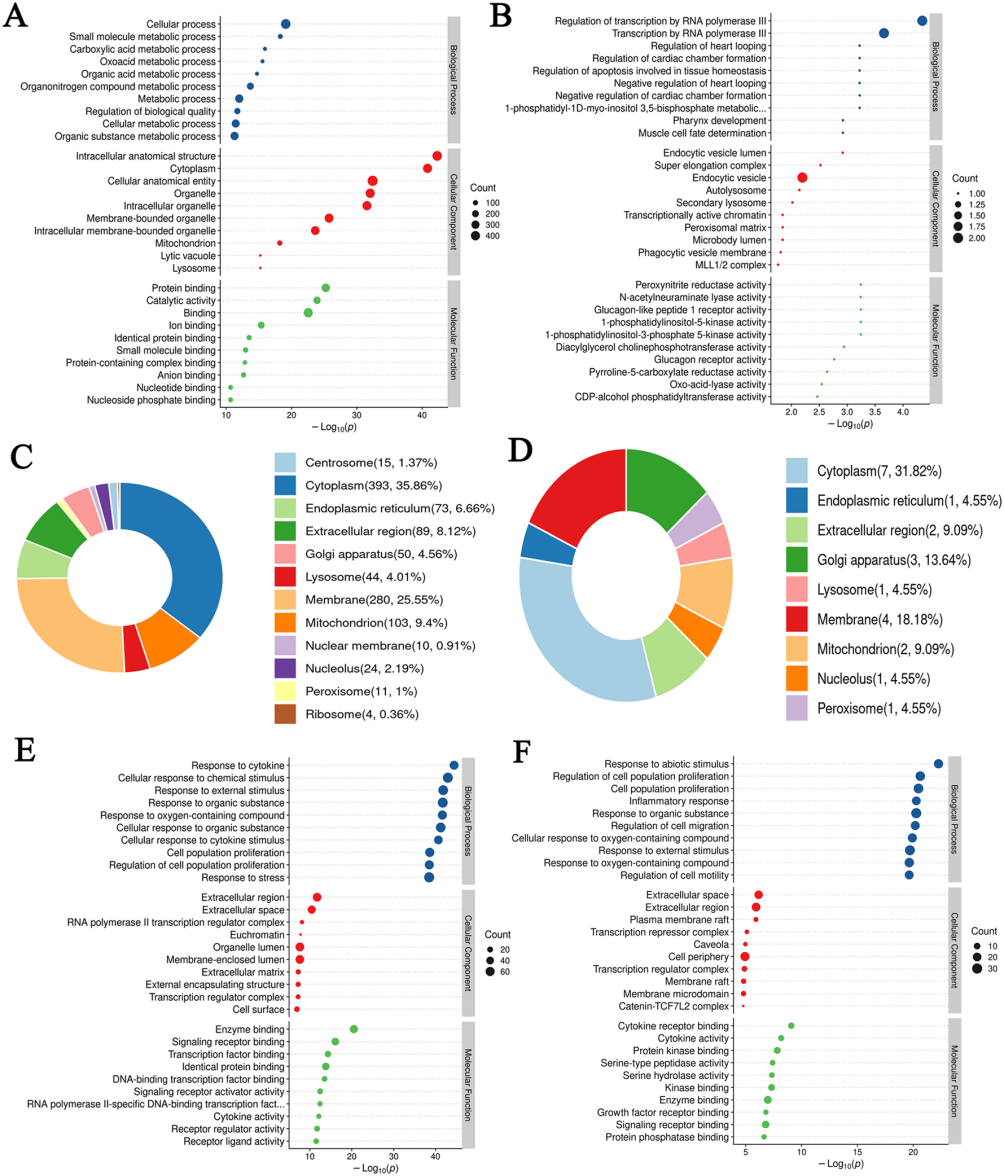
GO Function Enrichment Analysis. **A** Identified differential expression proteins between CC and PF groups. **B** Identified differential expression proteins between the GH group and the PF group. **C** Subcellular localization of differential expression proteins between CC and PF groups. **D** Subcellular localization of differential expression proteins between PF and GH groups. **E** GO analysis of targets of GHSPT (potential active ingredients) in treating PF. **F** GO analysis of targets of GHSPT (serum migrant compounds). *CC* control group, *PF* bleomycin-induced PF group, *GH* GHSPT treated bleomycin-induced PF group, *GHSPT* ginseng honeysuckle superfine powdered tea, *PF* pulmonary fibrosis

For the analysis of BP, CC and MF of differentially expression proteins (DEPs) between CC group and PF group, majority of obtained proteins was involved in cellular process, metabolic process, and related to intracellular anatomical structure, cytoplasm, and included protein binding, catalytic activity (Fig. 5A). For the analysis of BP, CC and MF of proteins normalized by GHSPT treatment was involved in regulation of transcription by RNA polymerase III, endocytic vesicle, and peroxynitrite reductase activity (Fig. 5B). The subcellular localization of 525 DEPs was mainly in the cytoplasm (35.86%), membrane (25.55%), and mitochondrion (9.4%) (Fig. 5C). The subcellular localization of back-regulated proteins by GHSPT was mainly in the cytoplasm (31.82%), membrane (18.18%) and golgi apparatus (13.64%) (Fig. 5D).

GO enrichment analyzed 92 drug targets of potential active ingredients and 33 drug targets of serum migrant compounds GHSPT in treating PF in BP, CC and MF (Fig. 5E and F). GO analysis of targets of GHSPT (potential active ingredients), BP-enriched targets mainly include response to cytokine, regulation of transcription by RNA polymerase III and regulation of apoptosis involved in tissue homeostasis. CC-enriched targets are related to intracellular anatomical structure, cytoplasm, and endocytic vesicle. Enriched MF mainly include enzyme binding, signaling receptor binding, catalytic activity, peroxynitrite reductase activity, and glucagon like peptide 1 receptor activity. GO analysis of targets of GHSPT (serum migrant compounds), BP-enriched targets mainly include cell population proliferation and inflammatory response. CC-enriched targets are related to extracellular region and cell periphery. Enriched MF mainly include enzyme binding and signaling receptor binding.

### KEGG pathway enrichment analysis

**Fig. 6:**
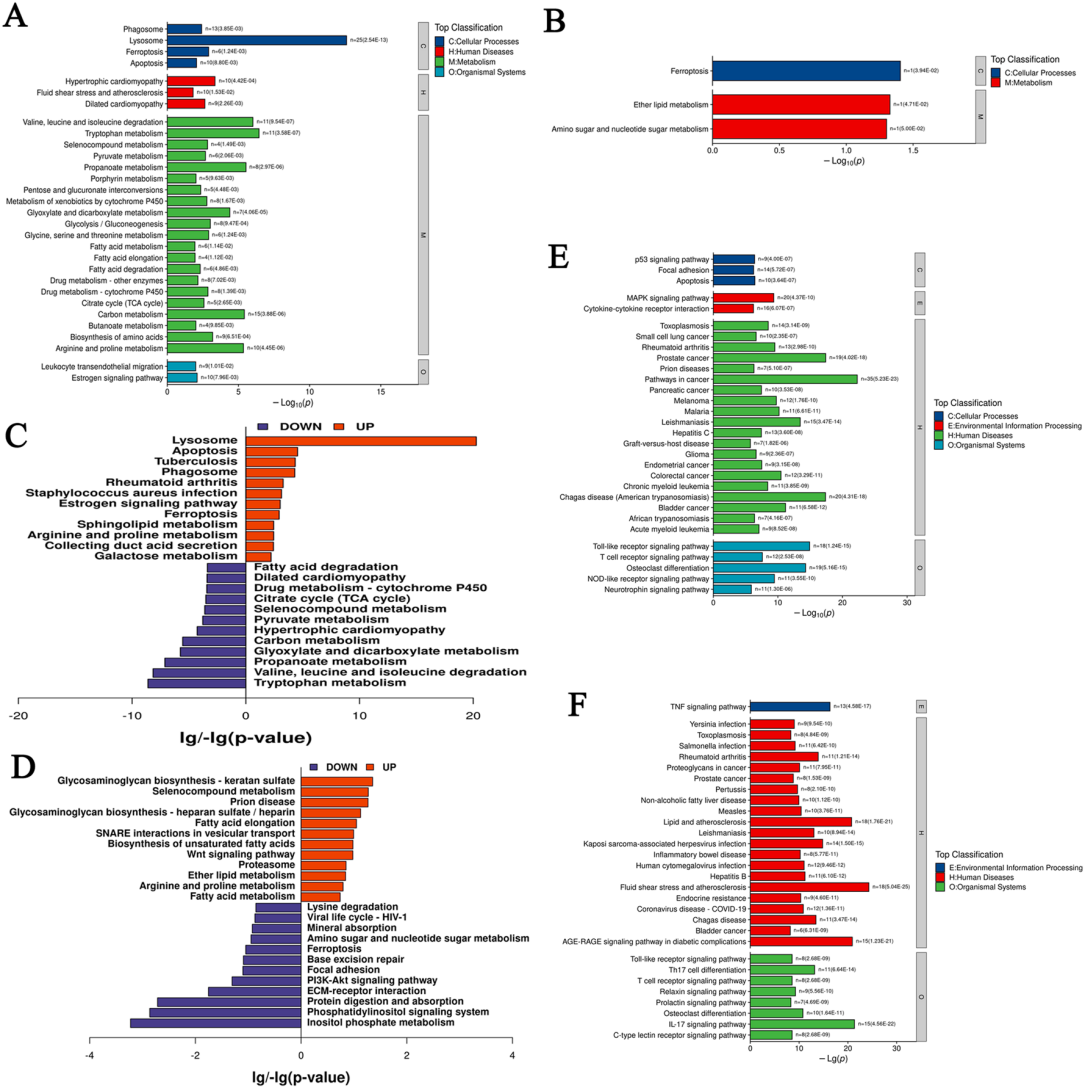
KEGG Pathway Enrichment Analysis. **A**: Identified differential expression proteins between CC and PF groups. **B**: Identified differential expression proteins between the GH group and the PF group. **C**: Up-regulation or down-regulation of KEGG signaling pathways in the PF group. **D**: Up-regulation or down-regulation of KEGG signaling pathways in the GH group. **E**: KEGG pathways analysis of targets of GHSPT (potential active ingredients) in treating PF. **F**: KEGG pathways analysis of targets of GHSPT (serum migrant compounds). CC: control group; PF: bleomycin-induced PF group; GH: GHSPT treated bleomycin-induced PF group. GHSPT: ginseng honeysuckle superfine powdered tea; PF: pulmonary fibrosis

KEGG signaling pathways analyzed 525 DEPs in PF groups, and 19 proteins treated with GHSPT (Fig. 6A, B, Additional file 4: Table S6 and S7). The up-regulation or down-regulation of KEGG signaling pathways in the PF group was shown in Fig. 6C. The up-regulation signaling pathways mainly include apoptosis, lysosome, ferroptosis, and estrogen signaling pathway. The down-regulation signaling pathways mainly include fatty acid degradation, pyruvate metabolism, and propanoate metabolism. The up-regulation or down-regulation of KEGG signaling pathways in the GH group was shown in Fig. 6D. The up-regulation signaling pathways mainly include fatty acid elongation, ether lipid metabolism, and Wnt signaling pathway. The down-regulation signaling pathways mainly include amino sugar and nucleotide sugar metabolism and PI3K-Akt signaling pathway. In this study, the significantly enriched KEGG signaling pathways analyzed 92 drug targets of potential active ingredients and 33 drug targets of serum migrant compounds GHSPT (Fig. 6E and F, Additional file 4: Table S8). The enriched signaling pathways mainly include apoptosis, P53 and MAPK signaling pathway, ferroptosis, lysosome, phagosome, NOD-like receptor, and VEGF signaling pathway ether lipid metabolism and amino sugar and nucleotide sugar metabolism (potential active ingredients), and TNF, Toll-like receptor and C-type lectin receptor signaling pathways (serum migrant compounds).

### PPI network analysis

**Fig. 7:**
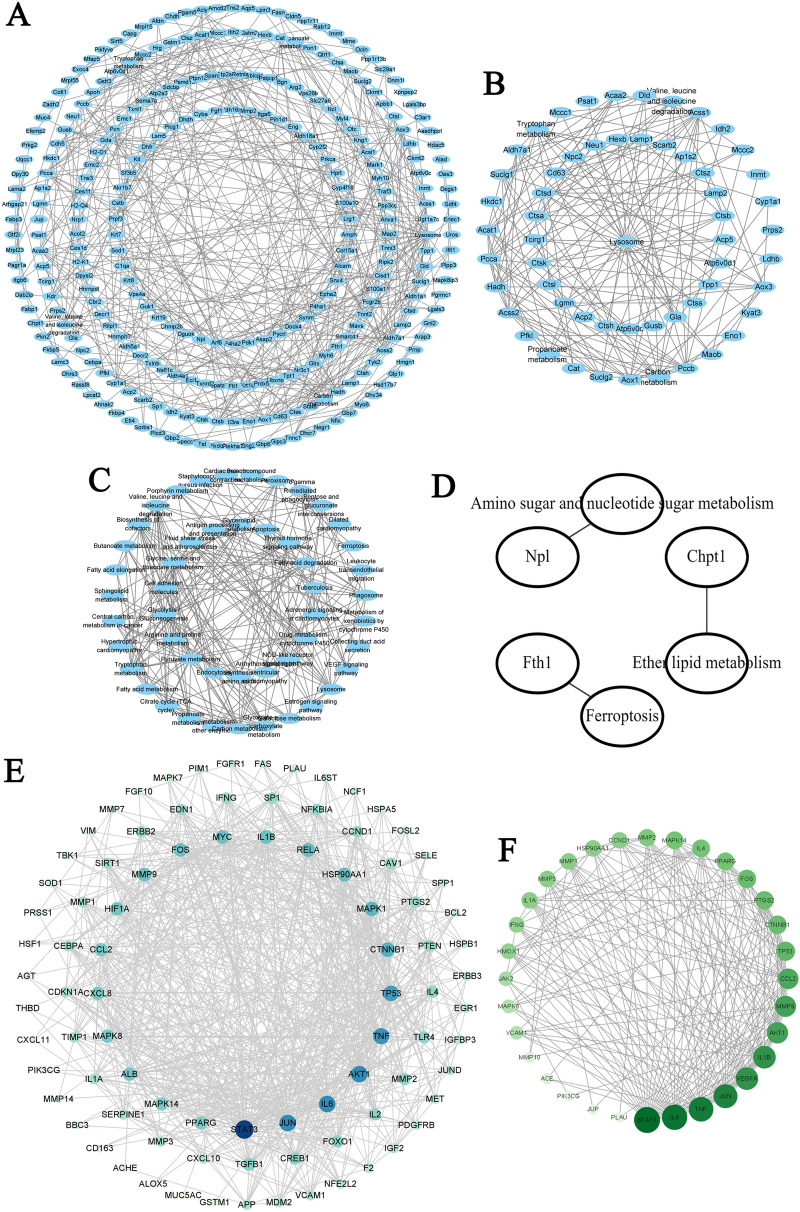
PPI network analysis. **A** PPI pathway-gene (all) network between PF group and CC group. **B** PPI pathway-gene (part) network between PF group and CC group. **C** PPI pathway- pathway network between PF group and CC group. **D** PPI pathway-gene network between GH group and PF group. **E** PPI networks of 92 targets of GHSPT (potential active ingredients) for the treatment of PF. **F** PPI networks of 33 targets of GHSPT (serum migrant compounds) for the treatment of PF. *CC* control group, *PF* bleomycin-induced the PF group, *GH* GHSPT treated bleomycin-induced the PF group, *GHSPT* ginseng honeysuckle superfine powdered tea, *PF* pulmonary fibrosis

In the PPI pathway-gene, and pathway-pathway network of 525 DEPs, the lysosome, apoptosis, ferroptosis, NOD-like receptor, VEGF, and estrogen signaling pathways were at the core (Fig. 7A, B and C). In the PPI pathway-gene network of 19 proteins treated with GHSPT, these networks were related to ferroptosis, ether lipid metabolism, and amino sugar and nucleotide sugar metabolism pathway (Fig. 7D). There are 92 nodes (representing targets of potential active ingredients) and 33 nodes (representing targets of serum migrant compounds) (Fig. 7E and F). The larger node indicates the greater degree value.

### Validation of drug targets of proteomics and network pharmacology with western blot

**Fig. 8:**
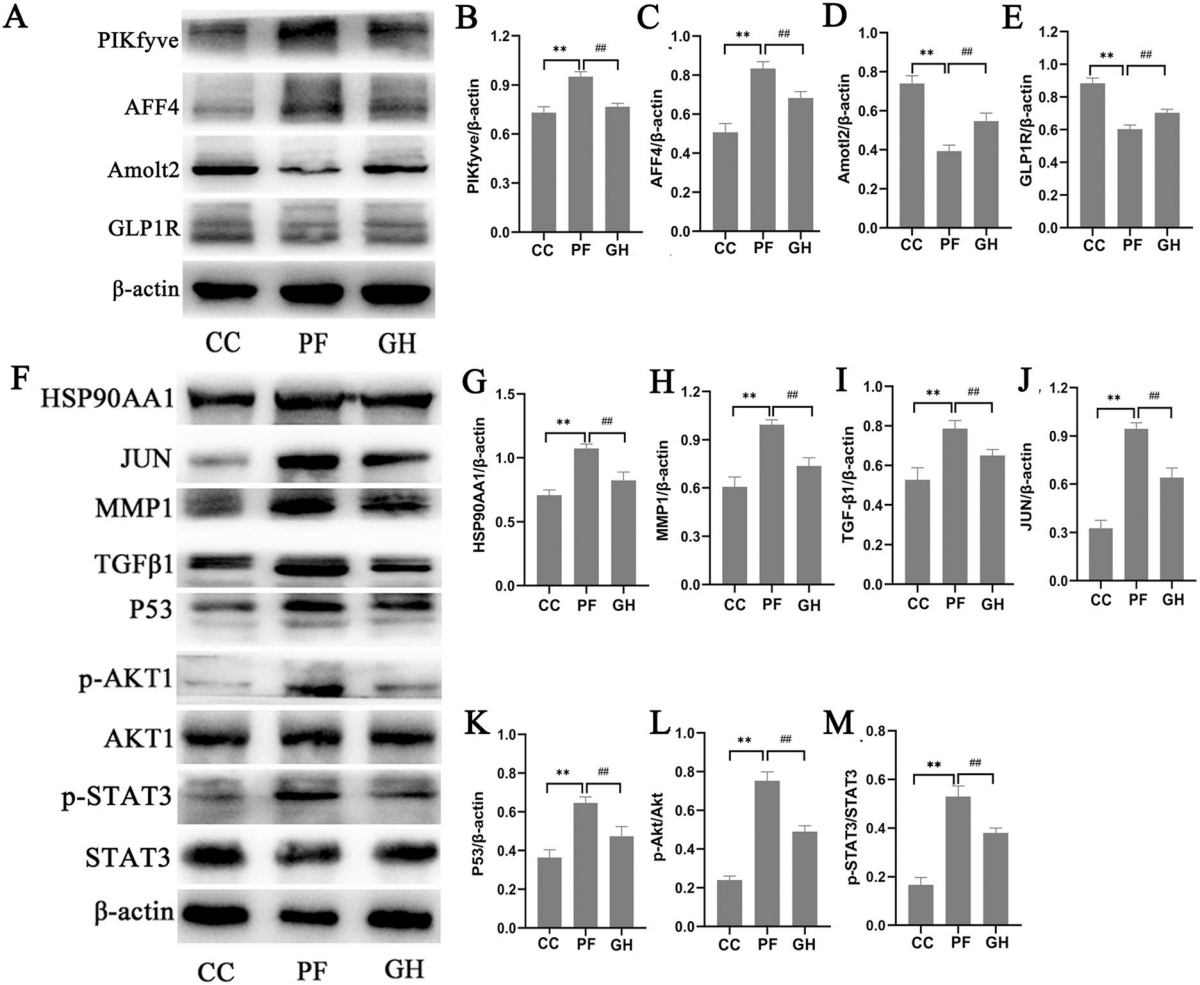
Validation of drug targets of proteomics and network pharmacology with western blot. **A** Western blot images of PIKfyve, AFF4, Amotl2, and GLP1R in the lung tissue. **B**–**E** Data were expressed as the expression ratio of PIKfyve/β-actin, AFF4/β-actin, Amotl2/β-actin, and GLP1R/β-actin (n = 3 per group). **F** Western blot images of HSP90AA1, MMP1, TGF-β1, JUN, P53, p-Akt, Akt, p-STAT3 and STAT3 in the lung tissue. **G**–**M**: Data were expressed as the expression ratio of HSP90AA1/β-actin, MMP1/β-actin, TGF-β1/β-actin, JUN/β-actin, P53/β-actin, p-Akt/Akt, and p-STAT3/STAT3 (n = 3 per group).^*^P < 0.05, ^**^P < 0.01 compared with CC group; ^#^*P* < 0.05, ^##^*P* < 0.01 compared with PF group. C*C:* control group, *PF* bleomycin-induced PF group, *GH* GHSPT treated bleomycin-induced PF group, *GHSPT* ginseng honeysuckle superfine powdered tea, *PF* pulmonary fibrosis

Among regulated proteins, we validated four differentially regulated proteins identified in our LC-MS/MS analysis using western blot assays. Consistently, the protein expression of PIKfyve and AFF4 was up-regulated in the lung tissues of the PF group. Moreover, the expression of Amotl2 and GLP1R was lower in the PF group than those in the CC group. GHSPT inhibited the protein expression of PIKfyve and AFF4, and increased the protein expression of Amotl2 and GLP1R in the PF group (Fig. 8A, B, C, D and E). Among 92 drug targets of network pharmacology, HSP90AA1, MMP1, TGF-β1, JUN, P53, p-Akt (Ser473) and p-STAT3 (Ser727) were validated with western blot. It was found that the protein expression of HSP90AA1, MMP1, TGF-β1, JUN, P53, p-Akt (Ser473) and p-STAT3 (Ser727) in the lung of PF group was higher than that in the CC group. After treatment with GHSPT, the expression of those was lower than that in the PF group (Fig. 8F, G, H, I, J, K and L, and Fig. 8M). They were consistent with the results of network pharmacology and LC-MS/MS analysis.

## Discussion

Pulmonary fibrosis is one of the main causes of death in elderly patients with chronic lung disease. It was previously thought to be caused by chronic inflammation. At present, the level of inflammatory factors, the proliferation of pulmonary fibroblasts, and the promotion of alveolar EMT play important roles in the development of pulmonary fibrosis [3, 12, 13]. Many research have reported that bleomycin is often used to build PF models. This model replicates the chronic progression and more obvious inflammatory response, similar to the pathogenesis of human PF. The mechanism of bleomycin-induced PF model is that bleomycin induces DNA breakage and oxidative stress, leading to cell apoptosis, promoting the production of inflammatory factors and fibrosis [14, 15]. In this study, bleomycin was successfully induced in a PF in mice model, which showed lung tissue inflammation and fibrosis, and regulated the progression of EMT. Moreover, GHSPT alleviated the PF and increase the survival rate of bleomycin-induced mice model. Thus, it is important to identify new therapeutic targets for PF and reveal the molecular mechanism of GHSPT.

In this study, we carried out a serum pharmacochemistry strategy to determine the effective ingredients in GHSPT. A total of 25 potential bioactive substances in vivo were successfully identified or tentatively characterized. Many ingredients played effective roles against PF via various molecular mechanisms, such as glycyrrhetinic acid, oleanolic acid, naringenin, calycosin, naringin, curdione, ginsenoside Rg1, isoliquiritigeni, pinocembrin, succinic acid, epicatechin, which further supported our serum pharmacochemistry findings [16–20]. Moreover, we analyzed the potential active ingredients and serum migrant compounds of GHSPT for the treatment of PF, and revealed the molecular mechanism and action targets based on proteomics and network pharmacology. The key drug targets of ingredient-target-disease and identified DEPs between CC and PF groups, and back-regulated proteins treated with GHSPT have been further obtained by GO and KEGG enrichment analysis. 19 drug targets treated with GHSPT were identified through proteomics. We validated the protein expression of PIKfyve, AFF4, Amotl2, and GLP1R with western blot. We also found that HSP90AA1, MMP1, TGF-β1, JUN, P53, and p-Akt are key targets in the network of GHSPT for the treatment of PF.

PIKfyve is a phosphoinositol 5 kinase that synthesizes PtdIns5P and PtdIns diphosphate. Su et al. reported that PIKfyve as a pharmacological target to interfere with host cell endocytosis is an effective way to block virus infection [21]. Moreover, inhibiting PIKfyve may be a new and effective method to reduce the proliferation and migration of vascular smooth muscle cells and vascular restenosis by affecting mammalian targets of rapamycin complex 1-mediated glucose utilization [22]. AFF4 is a member of the AF4 family and contains a serine-rich transcriptional activation domain. It is a vital transcription regulatory factor in many physiological processes. AFF4 can promote the invasion and migration of melanoma cells by mediating EMT and c-Jun activity [23]. EMT also plays an important role in the development of pulmonary fibrosis. In this study, GHSPT inhibited the protein expression of PIKfyve and AFF4. This might be a new strategy for treating for PF.

Amotl2 is a member of the angiomotin protein family and an important regulator of signal transduction and biological activity [24]. Amotl2 is located at the tight junction of cells and plays a key role in regulating cytoskeleton organization and cell polarity. Overexpression of Amotl2 inhibits TGF-β1 induced proliferation and ECM production by down-regulating YAP1 activation [25]. This indicates that Amotl2 plays an important role in airway remodeling of asthma. GLP1R is expressed in different regions of lung tissue, such as tracheal submucosal glands, alveolar type II cells, and pulmonary artery smooth muscle [26]. GLP1R activation plays an important role in lung function under normal and pathological conditions. GLP1R agonist (liraglutide) was able to reverse pulmonary fibrosis in the bleomycin-induced rat model [27]. Our study proved that the protective effect of GHSPT on PF is related to the regulation of Amotl2 and GLP1R expression.

HSP90 protein family is one of the most abundant molecular chaperones, which is highly conserved from bacteria to eukaryotes. There are two cytoplasmic isoforms of HSP90 in mammalian cells, including of HSP90α and HSP90β. HSP90AA1 is the HSP90α coding gene [28]. The expression of HSP90α is associated with a variety of physiological functions, not only to the heat shock response. HSP90 inhibition may also be a potential choice for inflammatory storms in acute respiratory patients [29, 30]. Bellaye et al. reported that an increase in circulating HSP90α patients with idiopathic pulmonary fibrosis, which was related with disease severity [31]. Our results found that HSP90AA1 can interact with up to 80 related active ingredients, and may potentially be the most important target for the treatment of PF with GHSPT.

MMP1 is a multifunctional protease responsible for degrading components of the extracellular matrix (ECM) and plays a key role in pulmonary fibrosis [32]. Inflammatory cytokines increase the expression of MMP1, and therefore stimulate airway remodeling. It was found that MMP1 was higher in lung tissues of patients with idiopathic pulmonary fibrosis. Inhibition of MMP1 with the small-molecule inhibitor GI254023X could significantly decrease lung fibrosis [33]. TGF- β1 also plays a central role in the pathogenesis of pulmonary fibrosis by promoting the differentiation of fibroblasts into myofibroblasts and produce excessive extracellular matrix, leading to the deterioration of pulmonary function [34]. MMP1 and TGF-β1 play an important role in the formation of pulmonary scarring and fibrosis. GHSPT inhibited the protein expression of HSP90AA1, MMP1, and TGF-β1 in the PF mice.

The enriched signaling pathways mainly include PI3K-Akt, P53, Wnt, NOD-like receptor, and VEGF signaling pathways in the proteomics and network pharmacology. Moreover, Akt, JUN, P53 and STAT3 are molecular hubs connecting many signaling pathways in cells including inflammatory response and biosynthesis of cytokines. Inhibition of PI3K-Akt, JUN, P53 and STAT3 can suppress inflammatory protein production and pulmonary fibrosis [35–37]. This might be a new treatment strategy for patients with pulmonary fibrosis, consistent with our enrichment analysis of drug targets.

## Conclusions

The treatment of pulmonary fibrosis by GHPST may be achieved by inhibiting inflammatory response, enhancing antioxidant effects, and decreasing the degree of EMT by multi-targets intervention and multiple signaling pathways. This study provides important molecular mechanisms and theoretical support of GHSPT in the treatment of pulmonary fibrosis. Further studies are needed to investigate the detailed molecular mechanisms at the cellular level. Therefore, these components of GHSPT can be used as dietary supplements or nutraceuticals in the treatment of fibrosis-related diseases including pulmonary fibrosis.

## Supplementary Information

**Additional file 1.** Protein identification table.

**Additional file 2.** Peptide identification table.

**Additional file 3.** Pos-neg table.

**Additional file 4**:** Table S1**. Identified significant differenced expression proteinsof the lung in the pulmonary fibrosis mice. **TableS2**. The potential activecomponents and ADME parameters of Ginseng honeysucklesuperfinepowdered tea. **TableS3**. The potential activeingredients and related targets of Ginseng honeysuckle superfinepowderedtea against pulmonary fibrosis. **TableS4**. The possible targets of Ginseng honeysuckle superfine powdered tea against pulmonaryfibrosis. **TableS5**. Serum migrant compounds and related targets of Ginseng honeysuckle superfinepowdered tea against pulmonaryfibrosis. **Table S6**. Pathway enrichment analysis of differential expressionproteins of the lungin the pulmonary fibrosis mice. **Table S7**. Pathway enrichment analysis of differential expressionproteins of the lung in the pulmonary fibrosis mice as reversed by Ginsenghoneysuckle superfine powdered tea. **Table S8**. Pathway enrichment analysis ofGinseng honeysuckle superfine powdered tea and pulmonaryfibrosis co-targeted genes. **Figure S1**. PCA plots for the proteomics of mice lung tissues. **Figure S2**. Metabolomic investigation of mice blood samples. (GH group, n=6;PF group, n=6).

## Data Availability

The data is available from the article and the Additional file 4. The proteomics data are available from the Proteome Xchange Consortium (PXD039732).

## Abbreviations

AFF4: FMR2 family member 4
α-SMA: α-Smooth muscle actin
Akt: Protein kinase B
EMT: Epithelial-to-mesenchymal transition
GHSPT: Ginseng honeysuckle superfine powdered tea
GLP1R: Glucagon-like peptide 1 receptor
HSP90: Heat shock protein 90
JUN: Transcription factor AP-1
MMP1: Matrix metalloproteinase 1
PF: Pulmonary fibrosis
PIKfyve: Phosphatidylinositol-3-phosphate 5-kinase
STAT3: Signal transducer and activator of transcription 3
TGF-β1: Transforming growth factor-β1
