# Supplementary material for: TMT proteomics analysis reveals the mechanism of bleomycin-induced pulmonary fibrosis and effects of Ginseng honeysuckle superfine powdered tea
Source: Chin Med. 2023 May 24;18:60. doi: 10.1186/s13020-023-00769-x (PMC10207824; doi:10.1186/s13020-023-00769-x)
Supplement: Supplementary file 4 — Additional file 4: Table S1. Identified significant differenced expression proteinsof the lung in the pulmonary fibrosis mice. TableS2. The potential activecomponents and ADME parameters of Ginseng honeysucklesuperfinepowdered tea. TableS3. The potential activeingredients and related targets of Ginseng honeysuckle superfinepowderedtea against pulmonary fibrosis. TableS4. The possible targets of Ginseng honeysuckle superfine powdered tea against pulmonaryfibrosis. TableS5. Serum migrant compounds and related targets of Ginseng honeysuckle superfinepowdered tea against pulmonaryfibrosis. Table S6. Pathway enrichment analysis of differential expressionproteins of the lungin the pulmonary fibrosis mice. Table S7. Pathway enrichment analysis of differential expressionproteins of the lung in the pulmonary fibrosis mice as reversed by Ginsenghoneysuckle superfine powdered tea. Table S8. Pathway enrichment analysis ofGinseng honeysuckle superfine powdered tea and pulmonaryfibrosis co-targeted genes. Figure S1. PCA plots for the proteomics of mice lung tissues. Figure S2. Metabolomic investigation of mice blood samples. (GH group, n=6;PF group, n=6). [file 13020_2023_769_MOESM4_ESM.docx]

**Supplementary Material**

**TMT proteomics analysis** **reveals the mechanism of** **bleomycin-induced pulmonary fibrosis and effects of Ginseng honeysuckle**

**superfine powdered tea**

Xiaoli Li^1^, Xin Yu^2,3,4^, Yuan Gao^2,3,4^, Wenqian Zhao^2,3,4^, Yajuan Wang^2,3,4^, Fei Yu^2,3,4^, Chunli Fu^2,3,4^, Haiqing Gao^2,3,4^, Mei Cheng^2,3,4*^, Baoying Li^3,5*^

*corresponding author

**Materials and methods**

**Materials**

Ginseng honeysuckle superfine powdered tea (GHSPT) detailed information:

(1) GHSPT (Batch No: 20210512) was produced by China Tea (Hunan) Co., Ltd (Changsha, China).

1) Proprietary product name: Ginseng honeysuckle superfine powdered tea.

2) The manufacturers’ name and supplier of the product: China Tea (Hunan) Co., Ltd (Changsha, China); China Tea Technology (Beijing) Co., Ltd (Beijing, China).

3) Pharmaceutical forms: ultrafine powder.

4) Batch number and date of production: batch No: 20210512; date of production: 20210512.

5) Details of storage conditions: store in a clean, dark, dry, odor-free, and well-ventilated environment.

(2) GHSPT components:

1) Panax ginseng C.A. Mey [Araliaceae; Radix et rhizoma]

2) Lonicera japonica Thunb [Caprifoliaceae; Flower stems, leaves et flowers]

3) Wurfbainia villosa var. villosa [Zingiberaceae; Fruit]

4) Citrus × aurantium f. deliciosa [Rutaceae; Peel]

5) Poria cocos [Polyporaceae; Sclerotium]

6) Glycyrrhiza uralensis Fisch. ex DC [Leguminosae; Radix et rhizoma]

7) Gardenia jasminoides J.Ellis [Rubiaceae; Fruit]

8) Camellia sinensis (L.) Kuntze [Theaceae; Leaves]

Table S1. Identified significant differenced expression proteins of the lung

in the pulmonary fibrosis mice

| Accession | Gene Symbol | Protein Name | Expression ratio (PF/CC) | *P* value |
| --- | --- | --- | --- | --- |
| P11679 | Krt8 | Keratin, type II cytoskeletal 8 | 1.33 | 7.48E-03 |
| Q9DCV7 | Krt7 | Keratin, type II cytoskeletal 7 | 1.77 | 4.65E-03 |
| P17182 | Eno1 | Alpha-enolase | 1.21 | 3.45E-02 |
| P19001 | Krt19 | Keratin, type I cytoskeletal 19 | 1.36 | 4.13E-02 |
| E9PZD8 | Cp | Ceruloplasmin | 1.24 | 9.10E-03 |
| Q60675 | Lama2 | Laminin subunit alpha-2 | 1.21 | 2.18E-02 |
| P05784 | Krt18 | Keratin, type I cytoskeletal 18 | 1.24 | 4.97E-02 |
| Q4FJV4 | Anxa1 | Annexin | 1.81 | 1.56E-02 |
| A0A7N9VR94 | Ahnak2 | AHNAK nucleoprotein 2 | 1.49 | 6.39E-03 |
| Q8CD23 | Ncl | Nucleolin | 1.21 | 4.08E-03 |
| P28653 | Bgn | Biglycan | 1.26 | 8.43E-03 |
| Q91XV3 | Basp1 | Brain acid soluble protein 1 | 1.67 | 9.37E-03 |
| Q3TXL5 | Ctsd | Cathepsin D | 2.01 | 5.97E-06 |
| P99029 | Prdx5 | Peroxiredoxin-5, mitochondrial | 1.28 | 8.13E-03 |
| O08677 | Kng1 | Kininogen-1 | 1.86 | 1.60E-02 |
| Q9CPX4 | Ftl1 | Ferritin | 1.65 | 1.23E-02 |
| Q03734 | Serpina3m | Serine protease inhibitor A3M | 2.08 | 7.36E-03 |
| Q3UJ44 | Capg | Macrophage-capping protein | 1.30 | 7.77E-04 |
| P09528 | Fth1 | Ferritin heavy chain | 1.41 | 6.28E-03 |
| P21956 | Mfge8 | Lactadherin | 1.24 | 2.41E-03 |
| P49935 | Ctsh | Pro-cathepsin H | 1.41 | 1.83E-02 |
| Q3V471 | Lgals3 | Galectin (Fragment) | 1.30 | 2.11E-03 |
| A0A498WGD8 | Txnl1 | Thioredoxin-like protein 1 | 1.23 | 2.62E-02 |
| O35382 | Exoc4 | Exocyst complex component 4 | 1.26 | 4.81E-02 |
| Q9WVJ2 | Psmd13 | 26S proteasome non-ATPase regulatory subunit 13 | 1.25 | 2.00E-02 |
| Q01339 | Apoh | Beta-2-glycoprotein 1 | 1.50 | 3.33E-02 |
| Q58E61 | Igh | Igh protein | 1.58 | 2.13E-02 |
| Q71RH5 | Gda | Guanine deaminase | 1.60 | 1.53E-02 |
| Q64282 | Ifit1 | Interferon-induced protein with tetratricopeptide repeats 1 | 1.58 | 1.66E-03 |
| Q8BSZ5 | Ctss | Uncharacterized protein | 1.64 | 1.26E-02 |
| P10605 | Ctsb | Cathepsin B | 1.67 | 1.45E-04 |
| P30412 | Ppic | Peptidyl-prolyl cis-trans isomerase C | 1.67 | 4.22E-02 |
| Q3ULW8 | Parp3 | Protein mono-ADP-ribosyltransferase PARP3 | 1.41 | 2.26E-02 |
| O35114 | Scarb2 | Lysosome membrane protein 2 | 1.35 | 5.51E-04 |
| E9Q2M9 | Wdfy4 | WD repeat- and FYVE domain-containing protein 4 | 1.22 | 5.00E-02 |
| Q64378 | Fkbp5 | Peptidyl-prolyl cis-trans isomerase FKBP5 | 1.71 | 2.31E-02 |
| Q61703 | Itih2 | Inter-alpha-trypsin inhibitor heavy chain H2 | 1.35 | 3.67E-02 |
| Q3TPF5 | Prmt1 | Methyltransf_25 domain-containing protein | 1.20 | 3.49E-02 |
| Q07797 | Lgals3bp | Galectin-3-binding protein | 1.32 | 3.60E-03 |
| A0A1L1SSH9 | Sparc | SPARC | 1.33 | 1.93E-02 |
| Q9QUR8 | Sema7a | Semaphorin-7A | 1.95 | 4.17E-02 |
| P30681 | Hmgb2 | High mobility group protein B2 | 1.26 | 3.13E-02 |
| Q640N1 | Aebp1 | Adipocyte enhancer-binding protein 1 | 1.56 | 1.32E-03 |
| Q3U3C2 | Npc2 | NPC intracellular cholesterol transporter 2 | 1.54 | 3.31E-02 |
| Q3UF30 | S100a10 | Protein S100-A10 | 1.33 | 4.93E-02 |
| Q9EST5 | Anp32b | Acidic leucine-rich nuclear phosphoprotein 32 family member B | 1.26 | 2.14E-02 |
| Q545N7 | Ckmt1 | Creatine kinase | 1.33 | 5.08E-03 |
| Q9CYL5 | Glipr2 | Golgi-associated plant pathogenesis-related protein 1 | 1.24 | 5.38E-03 |
| Q91W06 | Tcirg1 | V-type proton ATPase subunit a | 1.49 | 1.08E-02 |
| P00493 | Hprt | Hypoxanthine-guanine phosphoribosyltransferase | 1.31 | 5.72E-03 |
| P29621 | Serpina3c | Serine protease inhibitor A3C | 2.41 | 4.63E-03 |
| A2RTI3 | Lgmn | Legumain | 2.35 | 1.67E-02 |
| P12265 | Gusb | Beta-glucuronidase | 1.28 | 1.41E-02 |
| P51863 | Atp6v0d1 | V-type proton ATPase subunit d 1 | 1.22 | 4.51E-02 |
| P50543 | S100a11 | Protein S100-A11 | 1.57 | 3.02E-02 |
| A0A0R4J293 | Tgm1 | Protein-glutamine gamma-glutamyltransferase K | 1.30 | 1.20E-02 |
| F6VQH5 | Hnrnpdl | Heterogeneous nuclear ribonucleoprotein D-like | 1.34 | 2.70E-02 |
| A0A0R4J039 | Hrg | Histidine-rich glycoprotein | 1.48 | 2.88E-02 |
| P63028 | Tpt1 | Translationally-controlled tumor protein | 1.41 | 2.77E-02 |
| Q99J29 | Scpep1 | Carboxypeptidase | 1.42 | 2.70E-02 |
| O35326 | Srsf5 | Serine/arginine-rich splicing factor 5 | 1.34 | 1.93E-02 |
| P54923 | Adprh | ADP-ribosylhydrolase ARH1 | 1.24 | 2.29E-02 |
| Q9ES94 | Ctsz | Cathepsin X | 1.53 | 4.12E-03 |
| P20060 | Hexb | Beta-hexosaminidase subunit beta | 1.51 | 5.94E-03 |
| Q8CGE8 | Ifi205 | Interferon-activable protein 205-A | 1.38 | 3.85E-02 |
| P33434 | Mmp2 | 72 kDa type IV collagenase | 1.20 | 4.21E-02 |
| Q8BYU6 | Tor1aip2 | Torsin-1A-interacting protein 2 | 1.21 | 2.33E-02 |
| Q91YJ2 | Snx4 | Sorting nexin-4 | 1.30 | 4.17E-02 |
| P50427 | Sts | Steryl-sulfatase | 1.21 | 4.69E-03 |
| Q9D2D1 | Ctsa | Carboxypeptidase | 1.48 | 4.16E-03 |
| E9Q3F3 | Efemp2 | EGF-containing fibulin-like extracellular matrix protein 2 | 1.41 | 2.30E-03 |
| Q62426 | Cstb | Cystatin-B | 1.47 | 1.40E-04 |
| A0A1Y7VNM3 | Ctsl | Procathepsin L | 1.47 | 2.51E-02 |
| O35206 | Col15a1 | Collagen alpha-1(XV) chain | 1.23 | 4.05E-02 |
| Q544F6 | Cotl1 | Cotl1 protein | 1.50 | 4.20E-02 |
| E9PV48 | Ifit3b | Interferon-induced protein with tetratricopeptide repeats 3B | 1.24 | 3.62E-03 |
| Q6ZQM8 | Ugt1a7c | UDP-glucuronosyltransferase 1A7 | 1.29 | 5.04E-03 |
| A0A0R4J0H8 | Fndc3b | Fibronectin type III domain-containing protein 3B | 1.51 | 1.78E-02 |
| Q9QUH0 | Glrx | Glutaredoxin-1 | 1.25 | 1.84E-03 |
| Q61462 | Cyba | Cytochrome b-245 light chain | 1.54 | 4.84E-02 |
| O89086 | Rbm3 | RNA-binding protein 3 | 2.04 | 4.14E-02 |
| Q9DCJ9 | Npl | N-acetylneuraminate lyase | 1.51 | 2.47E-02 |
| Q8BGR9 | Ublcp1 | Ubiquitin-like domain-containing CTD phosphatase 1 | 1.25 | 9.08E-03 |
| Q9CRD2 | Emc2 | ER membrane protein complex subunit 2 | 1.25 | 1.53E-03 |
| Q3TWN8 | Aldh18a1 | Delta-1-pyrroline-5-carboxylate synthase | 1.29 | 4.07E-02 |
| A1L0V4 | H3c11 | Histone H3 | 1.41 | 3.23E-02 |
| Q71V27 | Csrp2 | Smooth muscle LIM protein | 1.27 | 8.57E-03 |
| Q8R1V4 | Tmed4 | Transmembrane emp24 domain-containing protein 4 | 1.23 | 1.02E-02 |
| A3KML5 | Atp6v0c | V-type proton ATPase proteolipid subunit | 1.25 | 4.78E-02 |
| Q9QZZ6 | Dpt | Dermatopontin | 1.27 | 2.69E-02 |
| Q3U6K9 | Psat1 | Phosphoserine aminotransferase | 1.36 | 3.46E-02 |
| Q8BG13 | Rbm3 | RNA-binding protein 3 | 1.66 | 4.27E-02 |
| Q9EPR5 | Sorcs2 | VPS10 domain-containing receptor SorCS2 | 1.26 | 4.81E-02 |
| Q6IFX2 | Krt42 | Keratin, type I cytoskeletal 42 | 1.34 | 2.38E-02 |
| A0A0A0UCD5 |  | Envelope protein | 1.21 | 3.97E-02 |
| A1L317 | Krt24 | Keratin, type I cytoskeletal 24 | 1.60 | 2.62E-02 |
| A0A0B4J1G1 | Fcgr2b | Fc receptor, IgG, low affinity IIb | 1.70 | 3.29E-02 |
| Q61112 | Sdf4 | 45 kDa calcium-binding protein | 1.27 | 3.17E-03 |
| Q9D809 | 2200002D01Rik | RIKEN cDNA 2200002D01 gene | 1.29 | 1.26E-02 |
| Q3UIW3 | Lamp2 | Uncharacterized protein | 1.26 | 6.06E-04 |
| Q5SX75 | P4ha2 | Procollagen-proline 4-dioxygenase | 1.64 | 4.64E-02 |
| Q9D5R5 | Ppp3cc | Serine/threonine-protein phosphatase | 1.94 | 4.26E-02 |
| Q8VH34 | Lamp1 | LAMP-1 | 1.28 | 5.70E-03 |
| D3YWT0 | Sec11a | Signal peptidase complex catalytic subunit SEC11 | 1.23 | 2.76E-02 |
| Q3TXB1 | C1qa | Complement C1q subcomponent subunit A | 1.55 | 2.09E-02 |
| Q8VEH8 | Erlec1 | Endoplasmic reticulum lectin 1 | 1.20 | 1.63E-03 |
| Q14AZ9 | Zadh2 | Zinc binding alcohol dehydrogenase, domain containing 2 | 1.94 | 4.13E-04 |
| Q60590 | Orm1 | Alpha-1-acid glycoprotein 1 | 1.75 | 7.00E-03 |
| B2KGT5 | Map2 | Microtubule-associated protein | 1.31 | 1.86E-02 |
| Q8VI93 | Oas3 | 2'-5'-oligoadenylate synthase 3 | 1.78 | 1.23E-02 |
| G3UZJ3 | Dtnb | Dystrobrevin beta | 1.20 | 1.60E-02 |
| Q91XL1 | Lrg1 | Leucine-rich HEV glycoprotein | 1.20 | 1.98E-03 |
| Q8CI95 | Osbpl11 | Oxysterol-binding protein-related protein 11 | 1.33 | 3.67E-02 |
| P21300 | Akr1b7 | Aldo-keto reductase family 1 member B7 | 1.36 | 4.09E-03 |
| Q78ZN4 | Retnla | XCP2 protein | 2.17 | 4.85E-02 |
| A1A4A7 | Pgam5 | Pgam5 protein | 1.27 | 1.06E-03 |
| Q5F228 | Slc36a1 | Proton-coupled amino acid transporter 1 | 1.79 | 5.38E-04 |
| Q6KCD5 | Nipbl | Nipped-B-like protein | 1.26 | 1.21E-02 |
| Q91W97 | Hkdc1 | Hexokinase HKDC1 | 1.60 | 1.88E-02 |
| Q9JL08 | S100A1 | Protein S100 | 1.58 | 1.29E-02 |
| Q9CPR5 | Mrpl15 | 39S ribosomal protein L15, mitochondrial | 1.25 | 1.70E-02 |
| Q3UBE4 | Sdcbp | Uncharacterized protein | 1.43 | 1.41E-02 |
| Q03145 | Epha2 | Ephrin type-A receptor 2 | 1.21 | 9.05E-03 |
| A0A0R4J0K5 | Cd84 | SLAM family member 5 | 1.23 | 3.98E-02 |
| Q3TXN3 | Mgat2 | Alpha-1,6-mannosyl-glycoprotein 2-beta-N-acetylglucosaminyltransferase | 1.21 | 9.26E-03 |
| Q8BYI6 | Lpcat2 | Lysophosphatidylcholine acyltransferase 2 | 1.50 | 3.74E-02 |
| Q8QZY6 | Tspan14 | Tetraspanin-14 | 1.21 | 2.24E-02 |
| Q9CZ83 | Mrpl55 | 39S ribosomal protein L55, mitochondrial | 1.21 | 8.84E-03 |
| Q8BT06 | Cd63 | Tetraspanin | 1.34 | 4.70E-02 |
| P00375 | Dhfr | Dihydrofolate reductase | 1.40 | 1.79E-03 |
| O08691 | Arg2 | Arginase-2, mitochondrial | 1.49 | 5.73E-04 |
| Q14AX6 | Cdk12 | Cyclin-dependent kinase 12 | 1.43 | 4.98E-02 |
| P30355 | Alox5ap | Arachidonate 5-lipoxygenase-activating protein | 1.46 | 1.94E-02 |
| Q4FJR0 | Nudt4 | Nudt4 protein | 1.25 | 3.79E-02 |
| Q923D4 | Sf3b5 | Splicing factor 3B subunit 5 | 1.69 | 1.42E-02 |
| Q8BHT6 | B3glct | Beta-1,3-glucosyltransferase | 1.33 | 1.82E-02 |
| Q8VCV1 | Abhd17c | Alpha/beta hydrolase domain-containing protein 17C | 1.36 | 4.35E-02 |
| A0A0R4J0P5 | Pstpip1 | Proline-serine-threonine phosphatase-interacting protein 1 | 1.50 | 2.32E-02 |
| Q9R0B6 | Lamc3 | Laminin subunit gamma-3 | 1.33 | 1.20E-02 |
| Q545T0 | Ctsk | Cathepsin K | 1.84 | 4.23E-03 |
| A2ADE0 | Plekhm2 | Pleckstrin homology domain-containing family M member 2 | 1.22 | 5.98E-03 |
| Q9R257 | Hebp1 | Heme-binding protein 1 | 1.35 | 1.42E-02 |
| A0A5F8MPS7 | Acp2 | Lysosomal acid phosphatase | 1.36 | 1.03E-02 |
| A2AGS6 | Trp53i11 | Tumor protein p53-inducible protein 11 | 1.22 | 3.11E-02 |
| B2RY30 | Exosc9 | Exosome complex component RRP45 | 1.36 | 9.75E-04 |
| Q6P8Y1 | Capsl | Calcyphosin-like protein | 1.30 | 3.64E-02 |
| A0A494BAS6 | Decr2 | Peroxisomal 2,4-dienoyl-CoA reductase [(3E)-enoyl-CoA-producing] | 1.23 | 4.68E-02 |
| Q9D3P8 | Plgrkt | Plasminogen receptor (KT) | 1.42 | 6.62E-03 |
| P51569 | Gla | Alpha-galactosidase A | 1.91 | 2.20E-03 |
| O09005 | Degs1 | Sphingolipid delta(4)-desaturase DES1 | 1.26 | 1.29E-03 |
| Q05117 | Acp5 | Tartrate-resistant acid phosphatase type 5 | 1.23 | 1.05E-02 |
| Q9ESC8 | Aff4 | AF4/FMR2 family member 4 | 2.52 | 5.17E-04 |
| A0A140LIT2 | Dhcr7 | 7-dehydrocholesterol reductase | 1.77 | 1.04E-02 |
| Q9JJC6 | Rilpl1 | RILP-like protein 1 | 1.59 | 2.54E-02 |
| Q14C50 | Slc27a6 | Solute carrier family 27 (Fatty acid transporter), member 6 | 1.21 | 2.85E-02 |
| Q3UDR8 | Yipf3 | Protein YIPF3 | 1.25 | 1.18E-03 |
| B2RRP1 | Tpgs1 | Gene trap ROSA b-geo 22 | 1.30 | 4.70E-02 |
| O88839 | Adam15 | Disintegrin and metalloproteinase domain-containing protein 15 | 1.27 | 1.27E-02 |
| Q9CV28 | Mindy3 | Ubiquitin carboxyl-terminal hydrolase MINDY-3 | 1.51 | 3.78E-02 |
| Q14DP4 | Pitpnm3 | Pitpnm3 protein | 1.47 | 8.77E-03 |
| H3BKC5 | Lsm5 | U6 snRNA-associated Sm-like protein LSm5 | 1.53 | 1.59E-02 |
| B2RU54 | Tmem104 | Transmembrane protein 104 | 1.28 | 8.15E-03 |
| Q9D211 | Cdkn2aipnl | CDKN2AIP N-terminal-like protein | 1.37 | 9.61E-03 |
| A0A0G2JEG3 | Slc35f6 | Solute carrier family 35 member F6 | 1.46 | 5.48E-03 |
| Q8CCH2 | Nhlrc3 | NHL repeat-containing protein 3 | 1.25 | 1.52E-02 |
| Q8BKZ2 | Gm9914 | Uncharacterized protein | 1.63 | 5.55E-03 |
| O70421 | Fzd1 | Frizzled-1 | 1.38 | 9.04E-03 |
| Q3UF68 | Prnp | Major prion protein | 1.34 | 4.27E-03 |
| D3Z5N5 | Pikfyve | 1-phosphatidylinositol-3-phosphate 5-kinase | 1.21 | 4.34E-03 |
| E9QMH1 | Apobec3 | DNA dC->dU-editing enzyme APOBEC-3 | 1.35 | 3.01E-02 |
| D3Z4D7 | Mfap5 | Microfibrillar-associated protein 5 | 1.63 | 3.72E-02 |
| O88736 | Hsd17b7 | 3-keto-steroid reductase/17-beta-hydroxysteroid dehydrogenase 7 | 1.59 | 1.72E-02 |
| Q8K262 | Plac9a | Placenta-specific protein 9 | 1.50 | 2.39E-02 |
| Q3TWJ1 | Rxrb | Retinoic acid receptor RXR-beta | 1.28 | 2.57E-02 |
| Q8BYP3 | Rhof | Rho-related GTP-binding protein RhoF | 1.26 | 3.37E-02 |
| A2ASZ8 | Slc25a25 | Calcium-binding mitochondrial carrier protein SCaMC-2 | 1.34 | 7.78E-04 |
| Q8C255 | Dpep2 | Dipeptidase 2 | 1.36 | 4.23E-02 |
| Q9ES34 | Ube3b | Ubiquitin-protein ligase E3B | 1.28 | 3.48E-02 |
| Q9WU81 | Slc37a2 | Glucose-6-phosphate exchanger SLC37A2 | 1.37 | 4.25E-02 |
| A0A0B4J1H6 | Igkv2-137 | Immunoglobulin kappa chain variable 2-137 | 1.25 | 1.37E-03 |
| Q9D8Z2 | Triap1 | TP53-regulated inhibitor of apoptosis 1 | 1.29 | 8.72E-03 |
| X5J4F8 |  | IgM heavy chain VDJ region | 1.50 | 2.70E-02 |
| Q99N16 | Cyp4f18 | Cytochrome P450 4F3 | 1.24 | 4.20E-02 |
| Q571G1 | Lpin3 | Phosphatidate phosphatase | 1.37 | 6.04E-03 |
| Q8C1E7 | Tmem120a | Ion channel TACAN | 1.31 | 1.97E-02 |
| Q9D6N1 | Car13 | Carbonic anhydrase 13 | 1.27 | 4.75E-02 |
| Q8VDP2 | C330007P06Rik | STING ER exit protein | 1.39 | 5.62E-03 |
| Q6KAT1 | Ltbp3 | MFLJ00070 protein | 1.35 | 3.53E-02 |
| Q3UL64 | Neu1 | Exo-alpha-sialidase | 1.21 | 2.21E-02 |
| A0A0R4J066 | Coq10b | Coenzyme Q-binding protein COQ10 homolog B, mitochondrial | 1.51 | 2.49E-02 |
| Q9DCL2 | Ciao2a | Cytosolic iron-sulfur assembly component 2A | 1.25 | 1.48E-02 |
| P58801 | Ripk2 | Receptor-interacting serine/threonine-protein kinase 2 | 1.72 | 7.88E-04 |
| A0A286YE23 | Il3ra | Interleukin-3 receptor subunit alpha | 1.21 | 4.64E-03 |
| Q9CR23 | Tmem9 | Proton-transporting V-type ATPase complex assembly regulator TMEM9 | 1.33 | 2.11E-02 |
| Q8K442 | Abca8a | ABC-type organic anion transporter ABCA8A | 1.25 | 1.14E-02 |
| Q8R054 | Srpx2 | Sushi repeat-containing protein SRPX2 | 1.22 | 2.37E-02 |
| A0A1B0GSZ9 | Mrpl23 | 39S ribosomal protein L23, mitochondrial | 1.27 | 1.53E-03 |
| Q3UHM2 | Traf3 | TNF receptor-associated factor | 1.20 | 4.21E-02 |
| O09047 | C3ar1 | C3a anaphylatoxin chemotactic receptor | 1.65 | 4.01E-02 |
| Q8BIG4 | Fbxo28 | F-box only protein 28 | 1.67 | 3.49E-02 |
| Q6PGA2 | Rrad | GTP-binding protein | 1.28 | 3.84E-03 |
| O09130 | Nfatc2ip | NFATC2-interacting protein | 1.38 | 2.44E-02 |
| Q9CQF6 | Aasdhppt | L-aminoadipate-semialdehyde dehydrogenase-phosphopantetheinyl transferase | 1.33 | 4.77E-02 |
| Q9JKC5 | Scn4a | Sodium channel alpha-subunit (Fragment) | 1.21 | 1.16E-02 |
| E0CYC4 | Cdk8 | Cyclin-dependent kinase 8 | 1.30 | 4.48E-04 |
| Q3TVA4 | Tex2 | Uncharacterized protein | 1.24 | 1.46E-02 |
| Q3UJR4 | Jtb | Uncharacterized protein | 1.41 | 4.90E-03 |
| Q9DBV3 | Dhx34 | Probable ATP-dependent RNA helicase DHX34 | 1.29 | 1.93E-02 |
| Q9JM03 | Slc15a2 | Proton-dependent high affinity oligopeptide transporter PepT2 | 1.46 | 2.47E-03 |
| Q499W5 | Neurl1a | Neuralized homolog 1A (Drosophila) | 1.41 | 1.78E-02 |
| Q9QXG1 | Muc4 | Mucin Muc4 | 1.42 | 4.48E-03 |
| Q8VC03 | Eml3 | Echinoderm microtubule-associated protein-like 3 | 3.04 | 2.54E-02 |
| Q3UG98 | Nat9 | Alpha/beta-tubulin-N-acetyltransferase 9 | 1.24 | 1.43E-02 |
| Q9JJW0 | Pxmp4 | Peroxisomal membrane protein 4 | 1.39 | 1.14E-03 |
| A0A5H1ZRN0 | Cep350 | Centrosome-associated protein 350 | 1.24 | 4.89E-02 |
| F7AGE9 | Obsl1 | Obscurin-like protein 1 | 1.49 | 2.99E-03 |
| B2RQQ1 | Myh6 | Myosin, heavy polypeptide 6, cardiac muscle, alpha | 0.59 | 1.85E-03 |
| P19096 | Fasn | Fatty acid synthase | 0.81 | 3.31E-02 |
| Q3UH59 | Myh10 | Myosin-10 | 0.79 | 1.67E-02 |
| P24549 | Aldh1a1 | Aldehyde dehydrogenase 1A1 | 0.60 | 1.15E-02 |
| O08553 | Dpysl2 | Dihydropyrimidinase-related protein 2 | 0.79 | 1.94E-02 |
| O55143 | Atp2a2 | Sarcoplasmic/endoplasmic reticulum calcium ATPase 2 | 0.82 | 2.56E-02 |
| A0A668KLV9 | Akap12 | A-kinase anchor protein 12 | 0.65 | 1.93E-03 |
| A0A571BG24 | Limch1 | LIM and calponin homology domains-containing protein 1 | 0.80 | 1.74E-02 |
| Q3UKH3 | Acaa2 | Uncharacterized protein | 0.73 | 2.33E-02 |
| Q8CAQ8 | Immt | MICOS complex subunit Mic60 | 0.83 | 7.88E-03 |
| Q8QZT1 | Acat1 | Acetyl-CoA acetyltransferase, mitochondrial | 0.74 | 7.00E-04 |
| Q1MWP9 | Ehd4 | EH-domain containing 4 | 0.71 | 4.84E-02 |
| Q3V117 | Acly | ATP-citrate synthase | 0.68 | 3.12E-03 |
| E9Q852 | Afdn | Afadin | 0.61 | 5.98E-03 |
| Q542P5 | Cbr2 | Uncharacterized protein | 0.49 | 5.82E-03 |
| P46735 | Myo1b | Unconventional myosin-Ib | 0.75 | 3.13E-02 |
| B1ATS5 | Atp2a3 | Calcium-transporting ATPase | 0.73 | 4.83E-02 |
| Q02257 | Jup | Junction plakoglobin | 0.78 | 2.98E-02 |
| P37040 | Por | NADPH--cytochrome P450 reductase | 0.75 | 3.20E-02 |
| Q8C6E3 | Cat | Catalase | 0.80 | 2.77E-02 |
| P97315 | Csrp1 | Cysteine and glycine-rich protein 1 | 0.81 | 4.10E-02 |
| Q8VCT4 | Ces1d | Carboxylesterase 1D | 0.66 | 2.64E-03 |
| P54071 | Idh2 | Isocitrate dehydrogenase [NADP], mitochondrial | 0.73 | 1.05E-02 |
| D3Z5J3 | Sorbs1 | Sorbin and SH3 domain-containing protein 1 | 0.80 | 2.41E-02 |
| P15626 | Gstm2 | Glutathione S-transferase Mu 2 | 0.75 | 1.69E-02 |
| P33267 | Cyp2f2 | Cytochrome P450 2F2 | 0.66 | 1.61E-02 |
| D3Z041 | Acsl1 | Arachidonate--CoA ligase | 0.69 | 2.31E-02 |
| E9Q175 | Myo6 | Unconventional myosin-VI | 0.80 | 3.36E-02 |
| Q9Z2I8 | Suclg2 | Succinate--CoA ligase [GDP-forming] subunit beta, mitochondrial | 0.78 | 2.06E-02 |
| Q8CGB6 | Tns2 | Tensin-2 | 0.79 | 3.90E-02 |
| Q8BW75 | Maob | Amine oxidase [flavin-containing] B | 0.76 | 1.04E-02 |
| Q91ZA3 | Pcca | Propionyl-CoA carboxylase alpha chain, mitochondrial | 0.77 | 8.36E-05 |
| Q6V956 | Aox3 | Aldehyde oxidase | 0.63 | 2.45E-02 |
| P10649 | Gstm1 | Glutathione S-transferase Mu 1 | 0.64 | 4.50E-02 |
| P40936 | Inmt | Indolethylamine N-methyltransferase | 0.48 | 2.04E-02 |
| A0PJE6 | Pccb | Pccb protein | 0.76 | 6.81E-03 |
| E9PUD2 | Dnm1l | Dynamin-1-like protein | 0.78 | 3.41E-02 |
| Q70IV5 | Synm | Synemin | 0.73 | 1.33E-04 |
| Q505N6 | Rps6ka1 | Ribosomal protein S6 kinase | 0.78 | 4.82E-02 |
| A2AT02 | Nsfl1c | NSFL1 cofactor p47 | 0.82 | 2.96E-02 |
| Q62467 | Scn7a | Sodium channel protein | 0.73 | 4.95E-02 |
| A0A6I8MX27 | Ldhb | L-lactate dehydrogenase | 0.75 | 6.92E-03 |
| Q9D172 | Gatd3a | Glutamine amidotransferase-like class 1 domain-containing protein 3, mitochondrial | 0.73 | 8.92E-04 |
| Q9CZ19 | Myl4 | Myosin light chain 4 | 0.60 | 5.45E-03 |
| Q9DC07 | Nebl | LIM zinc-binding domain-containing Nebulette | 0.76 | 4.21E-02 |
| Q61739 | Itga6 | Integrin alpha-6 | 0.79 | 3.71E-02 |
| Q8BJH2 | Add3 | Aldolase_II domain-containing protein | 0.77 | 6.09E-03 |
| E9Q3Q6 | Alcam | CD166 antigen | 0.75 | 4.49E-02 |
| P55284 | Cdh5 | Cadherin-5 | 0.78 | 1.71E-02 |
| Q3UNK1 | Add3 | Aldolase_II domain-containing protein | 0.82 | 3.54E-03 |
| P12382 | Pfkl | ATP-dependent 6-phosphofructokinase, liver type | 0.83 | 2.08E-03 |
| Q61391 | Mme | Neprilysin | 0.79 | 2.01E-02 |
| Q99NB1 | Acss1 | Acetyl-coenzyme A synthetase 2-like, mitochondrial | 0.81 | 9.80E-04 |
| P08228 | Sod1 | Superoxide dismutase [Cu-Zn] | 0.72 | 3.49E-02 |
| Q9Z0E6 | Gbp2 | Guanylate-binding protein 2 | 0.80 | 8.56E-03 |
| F8VQ28 | Pxn | Paxillin | 0.82 | 3.29E-02 |
| O08749 | Dld | Dihydrolipoyl dehydrogenase, mitochondrial | 0.83 | 6.77E-03 |
| W5XQG0 | H2-D1 | MHC class I antigen | 0.80 | 4.99E-02 |
| P42125 | Eci1 | Enoyl-CoA delta isomerase 1, mitochondrial | 0.72 | 2.54E-02 |
| P30416 | Fkbp4 | Peptidyl-prolyl cis-trans isomerase FKBP4 | 0.65 | 3.04E-02 |
| Q9DBF1 | Aldh7a1 | Alpha-aminoadipic semialdehyde dehydrogenase | 0.82 | 1.02E-02 |
| Q99JY3 | Gimap4 | GTPase IMAP family member 4 | 0.76 | 3.67E-02 |
| Q9CRB6 | Tppp3 | Tubulin polymerization-promoting protein family member 3 | 0.54 | 1.89E-02 |
| Q9WUM5 | Suclg1 | Succinate--CoA ligase [ADP/GDP-forming] subunit alpha, mitochondrial | 0.79 | 1.34E-02 |
| Q9QYR9 | Acot2 | Acyl-coenzyme A thioesterase 2, mitochondrial | 0.82 | 3.01E-02 |
| Q5SSZ5 | Tns3 | Tensin-3 | 0.81 | 4.61E-02 |
| A1L2Z3 | Emc1 | ER membrane protein complex subunit 1 | 0.81 | 2.15E-02 |
| Q8CHT0 | Aldh4a1 | Delta-1-pyrroline-5-carboxylate dehydrogenase, mitochondrial | 0.77 | 2.92E-03 |
| Q2M4I9 | Plcg1 | 1-phosphatidylinositol 4,5-bisphosphate phosphodiesterase gamma | 0.79 | 3.14E-02 |
| P97333 | Nrp1 | Neuropilin-1 | 0.78 | 3.53E-02 |
| B2RSU6 | Cgnl1 | Cingulin-like 1 | 0.80 | 3.96E-03 |
| Q9CQ62 | Decr1 | 2,4-dienoyl-CoA reductase [(3E)-enoyl-CoA-producing], mitochondrial | 0.83 | 1.19E-02 |
| Q3TFP8 | Pgrmc1 | Cytochrome b5 heme-binding domain-containing protein | 0.82 | 3.02E-02 |
| Q9DD05 | Alad | Delta-aminolevulinic acid dehydratase | 0.76 | 2.94E-02 |
| P01901 | H2-K1 | H-2 class I histocompatibility antigen, K-B alpha chain | 0.79 | 4.66E-03 |
| Q3ULD5 | Mccc2 | Methylcrotonoyl-CoA carboxylase beta chain, mitochondrial | 0.66 | 1.78E-03 |
| P52196 | Tst | Thiosulfate sulfurtransferase | 0.66 | 5.09E-03 |
| Q9DAR7 | Dcps | m7GpppX diphosphatase | 0.77 | 2.30E-02 |
| P59764 | Dock4 | Dedicator of cytokinesis protein 4 | 0.72 | 2.10E-02 |
| F7BJK1 | Pcdh1 | Protocadherin 1 | 0.77 | 3.23E-02 |
| Q63961 | Eng | Endoglin | 0.80 | 4.56E-02 |
| P62331 | Arf6 | ADP-ribosylation factor 6 | 0.79 | 4.89E-02 |
| A0A0R4J0J8 | Specc1l | Cytospin-A | 0.81 | 4.04E-02 |
| Q3TUQ7 | Prkaa1 | Acetyl-CoA carboxylase kinase | 0.82 | 1.34E-03 |
| Q61425 | Hadh | Hydroxyacyl-coenzyme A dehydrogenase, mitochondrial | 0.67 | 3.73E-02 |
| A0A5F8MP99 | Macf1 | Microtubule-actin cross-linking factor 1 | 0.66 | 1.31E-02 |
| Q99MR8 | Mccc1 | Methylcrotonoyl-CoA carboxylase subunit alpha, mitochondrial | 0.73 | 5.40E-03 |
| Q6P9J5 | Kank4 | KN motif and ankyrin repeat domain-containing protein 4 | 0.68 | 1.22E-02 |
| A2AQ25 | Etl4 | Sickle tail protein | 0.78 | 2.25E-02 |
| Q8CGF1 | Arhgap29 | Rho GTPase-activating protein 29 | 0.76 | 1.70E-02 |
| Q6P8I4 | Pcnp | PEST proteolytic signal-containing nuclear protein | 0.75 | 1.34E-02 |
| Q6P8J7 | Ckmt2 | Creatine kinase S-type, mitochondrial | 0.81 | 6.34E-03 |
| Q91Z40 | Gbp7 | Guanylate-binding protein 7 | 0.78 | 2.34E-02 |
| Q9DC11 | Plxdc2 | Plexin domain-containing protein 2 | 0.76 | 2.93E-02 |
| D3Z3N4 | Hnrnph3 | Heterogeneous nuclear ribonucleoprotein H3 | 0.80 | 6.42E-03 |
| A0A0G2JDV3 | Gbp6 | Guanylate-binding protein 6 | 0.77 | 3.37E-02 |
| P15306 | Thbd | Thrombomodulin | 0.70 | 3.24E-02 |
| Q8VCW8 | Acsf2 | Medium-chain acyl-CoA ligase ACSF2, mitochondrial | 0.77 | 1.43E-02 |
| E9Q7B0 | P4ha1 | Procollagen-proline 4-dioxygenase | 0.77 | 4.20E-02 |
| E9QP76 | Rtkn2 | Rhotekin-2 | 0.73 | 1.48E-02 |
| A2AR99 | Calcrl | Calcitonin gene-related peptide type 1 receptor | 0.70 | 1.72E-02 |
| A2AUX5 | Dab2ip | Disabled homolog 2-interacting protein | 0.82 | 9.04E-03 |
| Q8C0E2 | Vps26b | Vacuolar protein sorting-associated protein 26B | 0.80 | 9.43E-03 |
| A2CG35 | Rab12 | Ras-related protein Rab-12 | 0.80 | 1.29E-02 |
| Q95H92 | H2-Q4 | H2-gs10 protein | 0.76 | 1.24E-02 |
| Q9CXS4 | Cenpv | Centromere protein V | 0.73 | 4.97E-02 |
| Q4VA93 | Prkca | Protein kinase C | 0.74 | 1.78E-02 |
| Q8R387 | Aox1 | Aldehyde oxidase | 0.80 | 6.09E-03 |
| Q8BGZ7 | Krt75 | Keratin, type II cytoskeletal 75 | 0.54 | 2.23E-02 |
| Q91WS0 | Cisd1 | CDGSH iron-sulfur domain-containing protein 1 | 0.82 | 4.91E-02 |
| Q9DBE0 | Csad | Cysteine sulfinic acid decarboxylase | 0.81 | 1.17E-03 |
| P19123 | Tnnc1 | Troponin C, slow skeletal and cardiac muscles | 0.60 | 1.40E-02 |
| Q8K2C9 | Hacd3 | Very-long-chain (3R)-3-hydroxyacyl-CoA dehydratase 3 | 0.79 | 3.49E-02 |
| K3W4R7 | Tnnt2 | Troponin T, cardiac muscle | 0.60 | 2.19E-02 |
| B7ZN33 | Nisch | Nisch protein | 0.78 | 1.71E-02 |
| G3UZM9 | Pkn2 | Protein kinase C | 0.75 | 1.52E-02 |
| Q9CS42 | Prps2 | Ribose-phosphate pyrophosphokinase 2 | 0.81 | 4.08E-02 |
| Q8VCF0 | Mavs | Mitochondrial antiviral-signaling protein | 0.79 | 1.44E-03 |
| Q0P557 | Spata18 | Mitochondria-eating protein | 0.83 | 2.48E-02 |
| B2RS24 | Ocln | Occludin | 0.72 | 4.99E-02 |
| Q8R310 | Tmcc3 | Transmembrane and coiled-coil domain protein 3 | 0.77 | 2.56E-02 |
| Q9DBB8 | Dhdh | Trans-1,2-dihydrobenzene-1,2-diol dehydrogenase | 0.77 | 5.02E-03 |
| Q68FM4 | Ptprm | Protein-tyrosine-phosphatase | 0.66 | 7.69E-03 |
| P48787 | Tnni3 | Troponin I, cardiac muscle | 0.68 | 2.41E-03 |
| Q8CEW1 | Hmgn1 | Uncharacterized protein | 0.79 | 2.32E-02 |
| Q8BZI0 | Afap1l1 | Actin filament-associated protein 1-like 1 | 0.73 | 3.48E-02 |
| B2RWZ5 | Aqp5 | Aquaporin-5 | 0.74 | 2.13E-02 |
| Q6A074 | Asap2 | MKIAA0400 protein | 0.83 | 3.25E-02 |
| H3BL28 | Ppp1r9a | Protein phosphatase 1, regulatory subunit 9A | 0.80 | 1.81E-02 |
| Q8R5G7 | Arap3 | Arf-GAP with Rho-GAP domain, ANK repeat and PH domain-containing protein 3 | 0.81 | 1.85E-02 |
| P52430 | Pon1 | Serum paraoxonase/arylesterase 1 | 0.69 | 5.49E-04 |
| Q99L20 | Gstt3 | Glutathione S-transferase theta-3 | 0.83 | 3.97E-02 |
| Q8R4D4 | Stat6 | Signal transducer and activator of transcription | 0.74 | 2.35E-03 |
| Q8C541 | Faah | Fatty acid amide hydrolase | 0.81 | 2.71E-02 |
| Q8VHH5 | Agap3 | Arf-GAP with GTPase, ANK repeat and PH domain-containing protein 3 | 0.80 | 1.92E-02 |
| A0A0M3HEQ0 | Txnrd2 | Thioredoxin-disulfide reductase | 0.81 | 2.15E-02 |
| Q71RI9 | Kyat3 | Kynurenine-oxoglutarate transaminase 3 | 0.78 | 3.24E-02 |
| P05532 | Kit | Mast/stem cell growth factor receptor Kit | 0.79 | 6.36E-03 |
| Q99PL6 | Ubxn6 | UBX domain-containing protein 6 | 0.82 | 1.29E-02 |
| Q8CB30 | Krt23 | IF rod domain-containing protein | 0.82 | 1.89E-02 |
| Q922U1 | Prpf3 | U4/U6 small nuclear ribonucleoprotein Prp3 | 0.72 | 3.10E-02 |
| Q8BWF0 | Aldh5a1 | Succinate-semialdehyde dehydrogenase, mitochondrial | 0.82 | 1.05E-02 |
| P11404 | Fabp3 | Fatty acid-binding protein, heart | 0.70 | 2.87E-02 |
| Q8C141 | Acss2 | Propionate-CoA ligase | 0.79 | 1.23E-03 |
| A0A0R4J1G9 | Steap3 | Metalloreductase STEAP3 | 0.79 | 2.66E-02 |
| Q9QZH3 | Ppie | Peptidyl-prolyl cis-trans isomerase E | 0.76 | 3.75E-02 |
| Q0PD53 | Rab6b | Rab6B | 0.74 | 4.29E-02 |
| P12710 | Fabp1 | Fatty acid-binding protein, liver | 0.68 | 3.50E-04 |
| P59279 | Rab2b | Ras-related protein Rab-2B | 0.80 | 1.84E-02 |
| Q9CVF5 | Nqo2 | Flavodoxin_2 domain-containing protein | 0.76 | 7.47E-03 |
| P06537 | Nr3c1 | Glucocorticoid receptor | 0.80 | 8.03E-03 |
| Q91WU0 | Ces1f | Carboxylesterase 1F | 0.72 | 4.81E-02 |
| Q3UJY1 | Slc29a1 | Uncharacterized protein | 0.80 | 2.93E-02 |
| O88876 | Dhrs3 | Short-chain dehydrogenase/reductase 3 | 0.81 | 1.55E-02 |
| A0A0R4J094 | Fahd2a | Fumarylacetoacetate hydrolase domain-containing 2A | 0.77 | 4.31E-03 |
| Q99JY8 | Plpp3 | Phospholipid phosphatase 3 | 0.80 | 3.72E-02 |
| G3UW40 | Mcc | Mutated in colorectal cancers | 0.75 | 2.23E-02 |
| Q8VCC2 | Ces1g | Liver carboxylesterase 1 | 0.73 | 1.34E-02 |
| Q922E4 | Pcyt2 | Ethanolamine-phosphate cytidylyltransferase | 0.78 | 3.50E-03 |
| J3QNU1 | Ate1 | Arginyl-tRNA--protein transferase 1 | 0.81 | 2.83E-02 |
| A0A5H1ZRL3 | Nelfb | Negative elongation factor B | 0.77 | 3.94E-02 |
| P97872 | Fmo5 | Flavin-containing monooxygenase 5 | 0.71 | 1.49E-03 |
| A0A0U1RPX3 | Dguok | Deoxyguanosine kinase, mitochondrial | 0.67 | 4.57E-03 |
| Q3TDY6 | Tpp1 | Tripeptidyl-peptidase 1 | 0.81 | 4.71E-02 |
| Q8R1H0 | Hopx | Homeodomain-only protein | 0.82 | 3.76E-02 |
| Q61466 | Smarcd1 | SWI/SNF-related matrix-associated actin-dependent regulator of chromatin subfamily D member 1 | 0.80 | 2.17E-02 |
| Q62415 | Ppp1r13b | Apoptosis-stimulating of p53 protein 1 | 0.76 | 4.28E-03 |
| Q8VHJ5 | Mark1 | Serine/threonine-protein kinase MARK1 | 0.61 | 4.33E-03 |
| Q9Z0T9 | Itgb6 | Integrin beta-6 | 0.81 | 2.01E-02 |
| Q5FWH6 | Arhgef15 | Rho guanine nucleotide exchange factor 15 | 0.82 | 2.60E-02 |
| Q91VA0 | Acsm1 | Acyl-coenzyme A synthetase ACSM1, mitochondrial | 0.68 | 2.49E-03 |
| B9EKB3 | Wwp1 | E3 ubiquitin-protein ligase | 0.79 | 4.83E-02 |
| Q5CZY4 | Nfix | Nuclear factor 1 | 0.77 | 5.62E-03 |
| Q8VEJ9 | Vps4a | Vacuolar protein sorting-associated protein 4A | 0.83 | 2.38E-02 |
| Q3LAC4 | Prex2 | Phosphatidylinositol 3,4,5-trisphosphate-dependent Rac exchanger 2 protein | 0.71 | 1.98E-02 |
| E9QLK3 | Brat1 | BRCA1-associated ATM activator 1 | 0.80 | 2.01E-03 |
| A0A1Y7VM56 | Sirt5 | NAD-dependent protein deacylase sirtuin-5, mitochondrial | 0.82 | 3.33E-03 |
| A0A338P7L8 | Chmp2b | Charged multivesicular body protein 2b | 0.83 | 1.60E-02 |
| A0A1L1SSA8 | Tmem205 | Transmembrane protein 205 | 0.67 | 2.84E-04 |
| O89090 | Sp1 | Transcription factor Sp1 | 0.78 | 2.47E-02 |
| Q9DAK9 | Phpt1 | 14 kDa phosphohistidine phosphatase | 0.81 | 2.79E-02 |
| Q8VCP9 | Clec14a | C-type lectin domain family 14 member A | 0.54 | 2.48E-02 |
| O54942 | Cldn5 | Claudin-5 | 0.78 | 4.62E-02 |
| A0A668KM31 | Amph | Amphiphysin | 0.79 | 4.04E-02 |
| Q8R138 | Tmem119 | Transmembrane protein 119 | 0.69 | 1.92E-03 |
| Q9JM62 | Reep6 | Receptor expression-enhancing protein 6 | 0.70 | 1.81E-02 |
| Q6ZWS1 | Fgf1 | Multifunctional fusion protein | 0.77 | 1.05E-02 |
| D3YTR7 | Cap2 | Adenylyl cyclase-associated protein | 0.83 | 3.96E-02 |
| Q61624 | Zfp148 | Zinc finger protein 148 | 0.74 | 3.91E-03 |
| Q99JI1 | Mustn1 | Musculoskeletal embryonic nuclear protein 1 | 0.74 | 3.30E-03 |
| P63213 | Gng2 | Guanine nucleotide-binding protein G(I)/G(S)/G(O) subunit gamma-2 | 0.79 | 1.35E-02 |
| B1AVD2 | Xpnpep2 | Xaa-Pro aminopeptidase 2 | 0.69 | 3.95E-04 |
| Q91VK4 | Itm2c | Integral membrane protein 2C | 0.77 | 1.68E-02 |
| Q3UK79 | Gzma | Peptidase S1 domain-containing protein | 0.55 | 1.66E-02 |
| Q9CQV1 | Pam16 | Mitochondrial import inner membrane translocase subunit TIM16 | 0.68 | 2.93E-02 |
| Q80VJ2 | Sra1 | Steroid receptor RNA activator 1 | 0.74 | 6.20E-04 |
| A2AFS3 | Elapor1 | Endosome/lysosome-associated apoptosis and autophagy regulator 1 | 0.72 | 2.55E-02 |
| E9Q6H8 | Plekha5 | Pleckstrin homology domain-containing, family A member 5 | 0.78 | 4.07E-02 |
| Q8BH44 | Coro2b | Coronin-2B | 0.82 | 3.75E-02 |
| A0A1B0GSU0 | Aldh16a1 | Aldehyde dehydrogenase family 16 member A1 | 0.81 | 1.27E-02 |
| Q6W8Q3 | Pcp4l1 | Purkinje cell protein 4-like protein 1 | 0.51 | 1.25E-04 |
| Q8BG48 | Stk17b | Serine/threonine-protein kinase 17B | 0.73 | 3.96E-03 |
| Q8R411 | Myct1 | Myc target protein 1 | 0.79 | 9.93E-03 |
| A0A0A0MQE8 | Arhgap21 | Rho GTPase-activating protein 21 | 0.72 | 2.78E-03 |
| Q64520 | Guk1 | Guanylate kinase | 0.83 | 3.02E-02 |
| Q5FW53 | Mybphl | Myosin-binding protein H-like | 0.58 | 3.93E-03 |
| Q8C8R9 |  | Diacylglycerol kinase (ATP) | 0.79 | 2.65E-02 |
| E9Q3C1 | C2cd2 | C2 domain-containing protein 2 | 0.77 | 5.42E-03 |
| Q8BIU1 | Chdh | GMC_OxRdtase_N domain-containing protein | 0.78 | 1.64E-03 |
| Q8BFP9 | Pdk1 | [Pyruvate dehydrogenase (acetyl-transferring)] kinase isozyme 1, mitochondrial | 0.82 | 9.61E-03 |
| Q8K4P0 | Wdr33 | pre-mRNA 3' end processing protein WDR33 | 0.83 | 4.51E-04 |
| B9EIX2 | Cep170b | AW555464 protein | 0.82 | 1.22E-02 |
| Q8BSD5 | She | SH2 domain-containing adapter protein E | 0.73 | 1.20E-02 |
| Q05A20 | Cyp1a1 | Cytochrome P450 1A | 0.69 | 4.88E-03 |
| Q3URM1 | Clpb | Uncharacterized protein | 0.83 | 3.44E-02 |
| A0A087WR62 | Apbb1 | Amyloid beta precursor protein-binding family B member 1 | 0.76 | 3.09E-02 |
| A0A1Y7VM96 | Crppa | D-ribitol-5-phosphate cytidylyltransferase | 0.80 | 2.03E-02 |
| Q9DCC4 | Pycrl | Pyrroline-5-carboxylate reductase 3 | 0.81 | 2.62E-02 |
| Q8CJ96 | Rassf8 | Ras association domain-containing protein 8 | 0.81 | 7.34E-04 |
| Q6P6M7 | Sepsecs | O-phosphoseryl-tRNA(Sec) selenium transferase | 0.82 | 2.13E-02 |
| B1AS46 | Casz1 | Zinc finger protein castor homolog 1 | 0.79 | 2.65E-02 |
| Q8R5M0 | Gipc3 | PDZ domain-containing protein GIPC3 | 0.82 | 3.90E-02 |
| A0A338P6D4 | Heg1 | Protein HEG homolog 1 | 0.82 | 1.26E-02 |
| Q9JMA2 | Qtrt1 | Queuine tRNA-ribosyltransferase catalytic subunit 1 | 0.70 | 1.55E-02 |
| Q91WE4 | BC031181 | UPF0729 protein C18orf32 homolog | 0.76 | 3.24E-02 |
| Q9WV03 | Fam50a | Protein FAM50A | 0.81 | 3.22E-02 |
| A0A0N4SVE6 | Clec1a | C-type lectin domain family 1 member A (Fragment) | 0.75 | 2.57E-02 |
| A2AMH4 | Slc44a1 | Choline transporter-like protein | 0.82 | 4.71E-02 |
| Q3U2H8 | Cebpa | BZIP domain-containing protein | 0.75 | 4.32E-02 |
| P35918 | Kdr | Vascular endothelial growth factor receptor 2 | 0.78 | 8.75E-04 |
| B2RV71 | Card19 | CARD domain-containing protein | 0.82 | 1.06E-02 |
| Q8C4R2 | Prkg2 | cGMP-dependent protein kinase | 0.71 | 1.90E-02 |
| P97366 | Evi5 | Ecotropic viral integration site 5 protein | 0.61 | 5.87E-03 |
| Q80UK0 | Sestd1 | SEC14 domain and spectrin repeat-containing protein 1 | 0.83 | 2.39E-02 |
| Q8C0J6 | Sowahc | Ankyrin repeat domain-containing protein SOWAHC | 0.79 | 2.19E-02 |
| Q8CHX7 | Rftn2 | Raftlin-2 | 0.73 | 2.88E-04 |
| Q3V3N5 | Gnl2 | Nucleolar GTP-binding protein 2 (Fragment) | 0.80 | 2.82E-02 |
| A2AWS5 | Hdac5 | Histone deacetylase | 0.82 | 7.09E-03 |
| Q8BGY7 | Fam210a | Protein FAM210A | 0.80 | 3.15E-02 |
| A0A2R8VHI2 | Ift27 | Intraflagellar transport protein 27 homolog | 0.58 | 1.21E-02 |
| Q8BW87 | Ap1s2 | AP complex subunit sigma | 0.70 | 4.57E-03 |
| D3Z079 | Stxbp5 | Syntaxin-binding protein 5 | 0.77 | 8.07E-04 |
| B1AVN9 | Phactr2 | Phosphatase and actin regulator | 0.66 | 6.51E-04 |
| Q5SVF7 | Nipsnap1 | NIPSNAP domain-containing protein | 0.81 | 3.44E-02 |
| E9Q6E0 | Mapk8ip3 | C-Jun-amino-terminal kinase-interacting protein 3 | 0.74 | 1.15E-02 |
| G3UZU4 | Gtf2i | General transcription factor II-I | 0.79 | 7.45E-03 |
| P13609 | Srgn | Serglycin | 0.82 | 3.24E-02 |
| Q3U447 | Tyk2 | Tyrosine-protein kinase | 0.81 | 3.69E-02 |
| Q69ZR9 | Tasor | Protein TASOR | 0.83 | 2.32E-02 |
| D3YU39 | Chpt1 | Cholinephosphotransferase 1 | 0.72 | 2.76E-02 |
| Q9D7V1 | Sh2d4a | SH2 domain-containing protein 4A | 0.58 | 9.95E-04 |
| D3YXJ5 | Lratd2 | LRAT domain-containing 1 | 0.80 | 1.73E-03 |
| A0A0U1RPA0 | Plekha7 | Pleckstrin homology domain-containing family A member 7 | 0.80 | 3.56E-02 |
| A0A0A0MQ80 | Spata5 | Vesicle-fusing ATPase | 0.78 | 2.21E-02 |
| A5A4Z0 | Ppp1r11 | E3 ubiquitin-protein ligase PPP1R11 | 0.68 | 1.58E-02 |
| Q9R0M5 | Tpk1 | Thiamin pyrophosphokinase 1 | 0.80 | 1.77E-02 |
| A2ASX1 | Grb14 | Growth factor receptor-bound protein 14 | 0.69 | 2.53E-02 |
| Q05CS3 | Cyp39a1 | Cyp39a1 protein | 0.66 | 5.91E-03 |
| Q05BP5 | Aktip | Aktip protein | 0.69 | 2.29E-02 |
| Q60707 | Tbx2 | T-box transcription factor TBX2 | 0.78 | 4.54E-03 |
| A0A1L1ST06 | Nt5dc1 | 5'-nucleotidase domain-containing protein 1 | 0.79 | 1.14E-02 |
| H3BJM1 | Foxp1 | Forkhead box protein P1 | 0.75 | 2.99E-02 |
| Q9CQG9 | Tmem100 | Transmembrane protein 100 | 0.64 | 7.87E-04 |
| Q8BW94 | Dnah3 | Dynein axonemal heavy chain 3 | 0.53 | 2.47E-02 |
| O88986 | Gcat | 2-amino-3-ketobutyrate coenzyme A ligase, mitochondrial | 0.74 | 5.58E-04 |
| Q3V441 | Ptpn18 | Tyrosine-protein phosphatase non-receptor type 18 | 0.82 | 2.60E-02 |
| Q3US81 | Gpm6b | Uncharacterized protein | 0.74 | 1.16E-02 |
| A0A4W9 | Negr1 | Neuronal growth regulator 1 | 0.69 | 4.80E-02 |
| O35659 | Glp1r | Glucagon-like peptide 1 receptor | 0.60 | 2.09E-02 |
| Q8R4W6 | Pcolce2 | Procollagen C-endopeptidase enhancer 2 | 0.76 | 3.18E-02 |
| A0A1D5RM67 | Psme3ip1 | PSME3-interacting protein | 0.74 | 2.82E-02 |
| Q9CWU4 | 2410004B18Rik | UPF0690 protein C1orf52 homolog | 0.71 | 5.08E-03 |
| Q791N7 | Polr1h | DNA-directed RNA polymerase I subunit RPA12 | 0.77 | 5.23E-03 |
| A6PWX9 | Uqcc1 | Ubiquinol-cytochrome-c reductase complex assembly factor 1 | 0.77 | 2.05E-02 |
| Q9CPR7 | Sike1 | Suppressor of IKBKE 1 | 0.75 | 6.52E-03 |
| Q921T5 | Kdm5a | [Histone H3]-trimethyl-L-lysine(4) demethylase | 0.81 | 3.29E-03 |
| P70187 | Mfsd14a | Hippocampus abundant transcript 1 protein | 0.79 | 1.46E-04 |
| P11609 | Cd1d1 | Antigen-presenting glycoprotein CD1d1 | 0.78 | 3.90E-02 |
| Q8VBT1 | Txlnb | Beta-taxilin | 0.74 | 3.59E-02 |
| Q3TPL3 | Uros | Uroporphyrinogen-III synthase | 0.54 | 7.66E-03 |
| Q3TP05 | Amotl2 | Angiomotin-like protein 2 | 0.77 | 4.77E-02 |
| E0CZE8 | Tmbim6 | Bax inhibitor 1 | 0.79 | 1.50E-02 |
| Q99L02 | Pagr1a | PAXIP1-associated glutamate-rich protein 1A | 0.80 | 4.94E-02 |
| Q8BSI6 | R3hcc1 | R3H and coiled-coil domain-containing protein 1 | 0.69 | 2.55E-02 |
| Q6P5C5 | Smug1 | Single-strand selective monofunctional uracil DNA glycosylase | 0.78 | 3.17E-02 |
| Q3UTQ8 | Cdkl5 | Cyclin-dependent kinase-like 5 | 0.62 | 7.16E-03 |
| Q99LT0 | Dpy30 | Protein dpy-30 homolog | 0.82 | 1.00E-02 |
| Q99KK1 | Reep3 | Receptor expression-enhancing protein 3 | 0.82 | 3.58E-02 |
| Q8K2J0 | Plcd3 | 1-phosphatidylinositol 4,5-bisphosphate phosphodiesterase delta-3 | 0.78 | 2.51E-02 |
| Q9CQJ2 | Pih1d1 | PIH1 domain-containing protein 1 | 0.82 | 1.48E-02 |
| Q71RY3 | Clasrp | Suppressor-of-white-apricot-like protein 2 | 0.75 | 4.81E-03 |
| Q9CZ16 | Tmem178 | Transmembrane protein 178A | 0.75 | 3.22E-03 |
| B2RUK8 | Klhdc9 | Kelch domain containing 9 | 0.62 | 4.34E-02 |
| Q8CEB5 | Spata33 | Uncharacterized protein | 0.82 | 3.44E-02 |
| Q149H7 | Cenpu | Centromere protein U | 0.66 | 2.06E-02 |
| Q3U652 | Mcoln2 | PKD_channel domain-containing protein | 0.78 | 2.31E-03 |
| G3X9E9 | Wdr66 | WD repeat domain 66 | 0.68 | 1.28E-02 |
| Q3UBG2 | Pid1 | PTB-containing, cubilin and LRP1-interacting protein | 0.51 | 1.79E-03 |
| Q9D6V8 | Paip2 | Polyadenylate-binding protein-interacting protein 2 | 0.60 | 1.96E-02 |
| Q09XV5 | Chd8 | Chromodomain-helicase-DNA-binding protein 8 | 0.67 | 4.84E-03 |
| Q543H3 | Otc | Ornithine transcarbamylase, mitochondrial | 0.80 | 1.88E-02 |
| Q8K0C1 | Ipo13 | Importin-13 | 0.68 | 6.29E-03 |
| E9Q933 | Tmem11 | Transmembrane protein 11, mitochondrial | 0.81 | 3.13E-03 |
| Q9EQR4 | Clca3a2 | Chloride channel accessory 3A2 | 0.78 | 3.10E-03 |
| Q71M36 | Cspg5 | Chondroitin sulfate proteoglycan 5 | 0.74 | 7.05E-03 |
| Q9D8Z1 | Ascc1 | Activating signal cointegrator 1 complex subunit 1 | 0.66 | 1.29E-02 |
| A0A3B2WDB9 | Vmn2r57 | Vomeronasal 2, receptor 57 | 0.56 | 3.04E-04 |
| Q6KAN2 | mFLJ00311 | MFLJ00311 protein | 0.69 | 4.95E-02 |
| B7ZP07 | Usp17lf | Ubiquitin carboxyl-terminal hydrolase | 0.72 | 1.53E-02 |

Table S2 The potential active components and ADME parameters of Ginseng honeysuckle

superfine powdered tea

| Mol ID | Molecule Name | OB(%) | DL | Related target |
| --- | --- | --- | --- | --- |
| **Panax ginseng C.A. Mey (17)** | | | | |
| MOL000358 | beta-sitosterol | 36.91 | 0.75 | 38 |
| MOL000422 | Kaempferol | 41.88 | 0.24 | 63 |
| MOL000449 | Stigmasterol | 43.83 | 0.76 | 31 |
| MOL000787 | Fumarine | 59.26 | 0.83 | 28 |
| MOL002879 | Diop | 43.59 | 0.39 | 3 |
| MOL003648 | Inermin | 65.83 | 0.54 | 18 |
| MOL005308 | Aposiopolamine | 66.65 | 0.22 | 8 |
| MOL005317 | Deoxyharringtonine | 39.27 | 0.81 | 2 |
| MOL005318 | Dianthramine | 40.45 | 0.20 | 3 |
| MOL005320 | arachidonate | 45.57 | 0.20 | 4 |
| MOL005321 | Frutinone A | 65.90 | 0.34 | 16 |
| MOL005344 | Ginsenoside-Rh2 | 36.32 | 0.56 | 12 |
| MOL005348 | Ginsenoside-Rh4_qt | 31.11 | 0.78 | 2 |
| MOL005356 | Girinimbin | 61.22 | 0.31 | 10 |
| MOL005376 | Panaxadiol | 33.09 | 0.79 | 1 |
| MOL005384 | suchilactone | 57.52 | 0.56 | 16 |
| MOL005399 | Alexandrine_qt | 36.91 | 0.75 | 1 |
| **Lonicera japonica Thunb (17)** | | | | |
| MOL000006 | luteolin | 36.16 | 0.25 | 57 |
| MOL000098 | quercetin | 46.43 | 0.28 | 154 |
| MOL000358 | beta-sitosterol | 36.91 | 0.75 | 38 |
| MOL000422 | kaempferol | 41.88 | 0.24 | 63 |
| MOL000449 | Stigmasterol | 43.83 | 0.76 | 31 |
| MOL001494 | Mandenol | 42.00 | 0.19 | 3 |
| MOL001495 | Ethyl linolenate | 46.10 | 0.20 | 2 |
| MOL002773 | beta-carotene | 37.18 | 0.58 | 22 |
| MOL002914 | Eriodyctiol (flavanone) | 41.35 | 0.24 | 8 |
| MOL003006 | (-)-(3R,8S,9R,9aS,10aS)-9-ethenyl-8-(beta-D-glucopyranosyloxy)-2,3,9,9a,10,10a-hexahydro-5-oxo-5H,8H-pyrano[4,3-d]oxazolo[3,2-a]pyridine-3-carboxylic acid_qt | 87.47 | 0.23 | 4 |
| MOL003014 | secologanic dibutylacetal_ qt | 53.65 | 0.29 | 3 |
| MOL003036 | ZINC03978781 | 43.83 | 0.76 | 3 |
| MOL003044 | Chryseriol | 35.85 | 0.27 | 18 |
| MOL003095 | 5-hydroxy-7-methoxy-2-(3,4,5-trimethoxyphenyl) chromone | 51.96 | 0.41 | 26 |
| MOL003111 | Centauroside_qt | 55.79 | 0.50 | 9 |
| MOL003117 | Ioniceracetalides B_qt | 61.19 | 0.19 | 6 |
| MOL003128 | dinethylsecologanoside | 48.46 | 0.48 | 2 |
| **Wurfbainia villosa var. villosa (8)** | | | | |
| MOL000358 | beta-sitosterol | 36.91 | 0.75 | 38 |
| MOL000449 | Stigmasterol | 43.83 | 0.76 | 31 |
| MOL001755 | 24-Ethylcholest-4-en-3-one | 36.08 | 0.76 | 2 |
| MOL001771 | poriferast-5-en-3beta-ol | 36.91 | 0.75 | 2 |
| MOL001973 | Sitosteryl acetate | 40.39 | 0.85 | 1 |
| MOL007180 | vitamin-e | 32.29 | 0.70 | 1 |
| MOL007535 | (5S,8S,9S,10R,13R,14S,17R)-17-[(1R,4R)-4-ethyl-1,5-dimethylhexyl]-10,13-dimethyl-2,4,5,7,8,9,11,12,14, 15,16,17-dodecahydro-1H-cyclopenta[a]phenanthrene-3,6-dione | 33.12 | 0.79 | 1 |
| MOL007536 | Stigmasta-5,22-dien-3-beta-yl acetate | 46.44 | 0.86 | 1 |
| **Citrus × aurantium f. deliciosa (5)** | | | | |
| MOL000359 | sitosterol | 36.91 | 0.75 | 3 |
| MOL004328 | naringenin | 59.29 | 0.21 | 37 |
| MOL005100 | 5,7-dihydroxy-2-(3-hydroxy-4-methoxyphenyl) chroman-4-one | 47.74 | 0.27 | 10 |
| MOL005815 | Citromitin | 86.90 | 0.51 | 10 |
| MOL005828 | nobiletin | 61.67 | 0.52 | 35 |
| **Poria cocos (6)** | | | | |
| MOL000273 | (2R)-2-[(3S,5R,10S,13R, 14R,16R,17R)-3,16-dihydroxy-4,4,10,13,14-pentamethyl-2,3,5,6, 12,15, 16,17-octahydro-1H-cyclopenta[a]phenanthren-17-yl]-6-methylhept-5-enoic acid | 30.93 | 0.81 | 2 |
| MOL000275 | trametenolic acid | 38.71 | 0.80 | 1 |
| MOL000279 | Cerevisterol | 37.96 | 0.77 | 1 |
| MOL000282 | ergosta-7,22E-dien-3beta-ol | 43.51 | 0.72 | 1 |
| MOL000283 | Ergosterol peroxide | 40.36 | 0.81 | 1 |
| MOL000296 | hederagenin | 36.91 | 0.75 | 24 |
| **Glycyrrhiza uralensis Fisch. ex DC (88)** | | | | |
| MOL000098 | quercetin | 46.43 | 0.28 | 154 |
| MOL000211 | Mairin | 55.38 | 0.78 | 1 |
| MOL000239 | Jaranol | 50.83 | 0.29 | 13 |
| MOL000354 | isorhamnetin | 49.60 | 0.31 | 37 |
| MOL000359 | sitosterol | 36.91 | 0.75 | 3 |
| MOL000392 | formononetin | 69.67 | 0.21 | 39 |
| MOL000417 | Calycosin | 47.75 | 0.24 | 22 |
| MOL000422 | kaempferol | 41.88 | 0.24 | 63 |
| MOL000497 | licochalcone a | 40.79 | 0.29 | 32 |
| MOL000500 | Vestitol | 74.66 | 0.21 | 30 |
| MOL001484 | Inermine | 75.18 | 0.54 | 17 |
| MOL001792 | DFV | 32.76 | 0.18 | 12 |
| MOL002311 | Glycyrol | 90.78 | 0.67 | 11 |
| MOL002565 | Medicarpin | 49.22 | 0.34 | 34 |
| MOL003656 | Lupiwighteone | 51.64 | 0.37 | 21 |
| MOL003896 | 7-Methoxy-2-methyl isoflavone | 42.56 | 0.20 | 43 |
| MOL004328 | naringenin | 59.29 | 0.21 | 37 |
| MOL004805 | (2S)-2-[4-hydroxy-3-(3-methylbut-2-enyl)phenyl]-8,8-dimethyl-2,3-dihydropyrano[2,3-f]chromen-4-one | 31.79 | 0.72 | 12 |
| MOL004806 | euchrenone | 30.29 | 0.57 | 10 |
| MOL004808 | glyasperin B | 65.22 | 0.44 | 21 |
| MOL004810 | glyasperin F | 75.84 | 0.54 | 18 |
| MOL004811 | Glyasperin C | 45.56 | 0.40 | 24 |
| MOL004814 | Isotrifoliol | 31.94 | 0.42 | 14 |
| MOL004815 | (E)-1-(2,4-dihydroxyphenyl)-3-(2,2-dimethylchromen-6-yl)prop-2-en-1-one | 39.62 | 0.35 | 20 |
| MOL004820 | kanzonols W | 50.48 | 0.52 | 21 |
| MOL004824 | (2S)-6-(2,4-dihydroxyphenyl)-2-(2-hydroxypropan-2-yl)-4-methoxy-2,3-dihydrofuro[3,2-g]chromen-7-one | 60.25 | 0.63 | 21 |
| MOL004827 | Semilicoisoflavone B | 48.78 | 0.55 | 17 |
| MOL004828 | Glepidotin A | 44.72 | 0.35 | 26 |
| MOL004829 | Glepidotin B | 64.46 | 0.35 | 15 |
| MOL004833 | Phaseolinisoflavan | 32.01 | 0.45 | 22 |
| MOL004835 | Glypallichalcone | 61.60 | 0.19 | 27 |
| MOL004838 | 8-(6-hydroxy-2-benzofuranyl)-2,2-dimethyl-5-chromenol | 58.44 | 0.38 | 6 |
| MOL004841 | Licochalcone B | 76.76 | 0.19 | 19 |
| MOL004848 | licochalcone G | 49.25 | 0.32 | 17 |
| MOL004849 | 3-(2,4-dihydroxyphenyl)-8-(1,1-dimethylprop-2-enyl)-7-hydroxy-5-methoxy-coumarin | 59.62 | 0.43 | 23 |
| MOL004855 | Licoricone | 63.58 | 0.47 | 15 |
| MOL004856 | Gancaonin A | 51.08 | 0.40 | 20 |
| MOL004857 | Gancaonin B | 48.79 | 0.45 | 22 |
| MOL004863 | 3-(3,4-dihydroxyphenyl)-5,7-dihydroxy-8-(3-methylbut-2-enyl)chromone | 66.37 | 0.41 | 18 |
| MOL004864 | 5,7-dihydroxy-3-(4-methoxyphenyl)-8-(3-methylbut-2-enyl)chromone | 30.49 | 0.41 | 20 |
| MOL004866 | 2-(3,4-dihydroxyphenyl)-5,7-dihydroxy-6-(3-methylbut-2-enyl)chromone | 44.15 | 0.41 | 16 |
| MOL004879 | Glycyrin | 52.61 | 0.47 | 17 |
| MOL004882 | Licocoumarone | 33.21 | 0.36 | 7 |
| MOL004883 | Licoisoflavone | 41.61 | 0.42 | 19 |
| MOL004884 | Licoisoflavone B | 38.93 | 0.55 | 17 |
| MOL004885 | licoisoflavanone | 52.47 | 0.54 | 20 |
| MOL004891 | shinpterocarpin | 80.30 | 0.73 | 30 |
| MOL004898 | (E)-3-[3,4-dihydroxy-5-(3-methylbut-2-enyl)phenyl]-1-(2,4-dihydroxyphenyl)prop-2-en-1-one | 46.27 | 0.31 | 12 |
| MOL004903 | liquiritin | 65.69 | 0.74 | 6 |
| MOL004904 | licopyranocoumarin | 80.36 | 0.65 | 16 |
| MOL004907 | Glyzaglabrin | 61.07 | 0.35 | 18 |
| MOL004908 | Glabridin | 53.25 | 0.47 | 25 |
| MOL004910 | Glabranin | 52.90 | 0.31 | 11 |
| MOL004911 | Glabrene | 46.27 | 0.44 | 19 |
| MOL004912 | Glabrone | 52.51 | 0.50 | 21 |
| MOL004913 | 1,3-dihydroxy-9-methoxy-6-benzofurano[3,2-c]chromenone | 48.14 | 0.43 | 10 |
| MOL004914 | 1,3-dihydroxy-8,9-dimethoxy-6-benzofurano[3,2-c]chromenone | 62.90 | 0.53 | 9 |
| MOL004915 | Eurycarpin A | 43.28 | 0.37 | 19 |
| MOL004924 | (-)-Medicocarpin | 40.99 | 0.95 | 2 |
| MOL004935 | Sigmoidin-B | 34.88 | 0.41 | 6 |
| MOL004941 | (2R)-7-hydroxy-2-(4-hydroxyphenyl)chroman-4-one | 71.12 | 0.18 | 15 |
| MOL004945 | (2S)-7-hydroxy-2-(4-hydroxyphenyl)-8-(3-methylbut-2-enyl)chroman-4-one | 36.57 | 0.32 | 12 |
| MOL004948 | Isoglycyrol | 44.70 | 0.84 | 7 |
| MOL004949 | Isolicoflavonol | 45.17 | 0.42 | 15 |
| MOL004957 | HMO | 38.37 | 0.21 | 27 |
| MOL004959 | 1-Methoxyphaseollidin | 69.98 | 0.64 | 29 |
| MOL004961 | Quercetin der. | 46.45 | 0.33 | 17 |
| MOL004966 | 3'-Hydroxy-4'-O-Methylglabridin | 43.71 | 0.57 | 28 |
| MOL004974 | 3'-Methoxyglabridin | 46.16 | 0.57 | 28 |
| MOL004978 | 2-[(3R)-8,8-dimethyl-3,4-dihydro-2H-pyrano[6,5-f]chromen-3-yl]-5-methoxyphenol | 36.21 | 0.52 | 31 |
| MOL004980 | Inflacoumarin A | 39.71 | 0.33 | 15 |
| MOL004985 | icos-5-enoic acid | 30.70 | 0.20 | 1 |
| MOL004988 | Kanzonol F | 32.47 | 0.89 | 8 |
| MOL004989 | 6-prenylated eriodictyol | 39.22 | 0.41 | 8 |
| MOL004990 | 7,2',4'-trihydroxy-5-methoxy-3-arylcoumarin | 83.71 | 0.27 | 15 |
| MOL004991 | 7-Acetoxy-2-methylisoflavone | 38.92 | 0.26 | 25 |
| MOL004993 | 8-prenylated eriodictyol | 53.79 | 0.40 | 8 |
| MOL004996 | gadelaidic acid | 30.70 | 0.20 | 1 |
| MOL005000 | Gancaonin G | 60.44 | 0.39 | 20 |
| MOL005001 | Gancaonin H | 50.10 | 0.78 | 12 |
| MOL005003 | Licoagrocarpin | 58.81 | 0.58 | 29 |
| MOL005007 | Glyasperins M | 72.67 | 0.59 | 26 |
| MOL005008 | Glycyrrhiza flavonol A | 41.28 | 0.60 | 17 |
| MOL005012 | Licoagroisoflavone | 57.28 | 0.49 | 18 |
| MOL005016 | Odoratin | 49.95 | 0.30 | 20 |
| MOL005017 | Phaseol | 78.77 | 0.58 | 14 |
| MOL005018 | Xambioona | 54.85 | 0.87 | 8 |
| MOL005020 | dehydroglyasperins C | 53.82 | 0.37 | 18 |
| **Gardenia jasminoides J.Ellis (12)** | | | | |
| MOL000098 | quercetin | 46.43 | 0.28 | 154 |
| MOL000358 | beta-sitosterol | 36.91 | 0.75 | 38 |
| MOL000422 | kaempferol | 41.88 | 0.24 | 63 |
| MOL000449 | Stigmasterol | 43.83 | 0.76 | 31 |
| MOL001406 | crocetin | 35.30 | 0.26 | 14 |
| MOL001494 | Mandenol | 42.00 | 0.19 | 3 |
| MOL001941 | Ammidin | 34.55 | 0.22 | 8 |
| MOL001942 | isoimperatorin | 45.46 | 0.23 | 1 |
| MOL002883 | Ethyl oleate (NF) | 32.40 | 0.19 | 1 |
| MOL003095 | 5-hydroxy-7-methoxy-2-(3,4,5-trimethoxyphenyl) chromone | 51.96 | 0.41 | 26 |
| MOL004561 | Sudan III | 84.07 | 0.59 | 13 |
| MOL007245 | 3-Methylkempferol | 60.16 | 0.26 | 11 |
| **Camellia sinensis (L.) Kuntze (6)** | | | | |
| MOL000492 | Catechin | 54.83 | 0.24 | 11 |
| MOL006504 | Catechin gallate | 53.57 | 0.75 | 1 |
| MOL006821 | Epigallocatechin gallate (EGCG) | 55.09 | 0.77 | 140 |
| MOL000569 | m-Digallic acid | 61.85 | 0.26 | 3 |
| MOL000422 | kaempferol | 41.88 | 0.24 | 63 |
| MOL000098 | quercetin | 46.43 | 0.28 | 154 |

Table S3 The potential active ingredients and related targets of Ginseng honeysuckle superfine

powdered tea against pulmonary fibrosis

| Mol ID | Ingredient | Medicine | Target for pulmonary fibrosis |
| --- | --- | --- | --- |
| MOL000098 | quercetin | Lonicera japonica Thunb, Glycyrrhiza uralensis Fisch. ex DC, Gardenia jasminoides J.Ellis, Camellia sinensis (L.) Kuntze | ACHE,AKT1,ALOX5,BCL2,CAV1,CCL2,CCND1,CDKN1A,CXCL10,CXCL11,CXCL8,ERBB2,ERBB3,F2,FOS,GSTM1,HAS2,HIF1A,HSF1,HSP90AA1,HSPA5,HSPB1,IFNG,IGF2,IGFBP3,IL1A,IL1B,IL2,IL6,JUN,MAPK1,MMP1,MMP2,MMP3,MMP9,MYC,NCF1,NFE2L2,NFKBIA,PIK3CG,PLAU,PPARG,PRSS1,PTEN,PTGS2,RELA,SELE,SERPINE1,SOD1,SPP1,TGFB1,THBD,TNF,TP53,VCAM1 |
| MOL006821 | Epigallocatechin gallate (EGCG) | Camellia sinensis (L.) Kuntze | PTGS2,BCL2,JUN,PPARG,RELA,AKT1,TNF,MAPK8,MMP1,PLAU,NFKBIA,CCND1,CDKN1A,MMP2,MMP9,MAPK1,IL6,TP53,MDM2,APP,ERBB2,MMP3,FOS,SOD1,HIF1A,HSPA5,CAV1,TIMP1,CREB1,STAT3,FOSL2,TBK1,AGT,FGFR1,EGR1,FGF10,PDGFRB,MMP14,BBC3,JUND,MMP7,EDN1,VIM,FOXO1,FAS,CEBPA,TLR4,SP1,SRD5A2,IL6ST,MAPK7,MUC5AC |
| MOL000006 | luteolin | Lonicera japonica Thunb | PTGS2,HSP90AA1,PIK3CG,JUN,PPARG,PRSS1,RELA,AKT1,TNF,MMP1,NFKBIA,IFNG,CCND1,CDKN1A,MMP2,MMP9,MAPK1,IL6,TP53,MDM2,APP,ERBB2,IL2,IL4,MET |
| MOL000422 | kaempferol | Panax ginseng C.A. Mey, Lonicera japonica Thunb, Glycyrrhiza uralensis Fisch. ex DC, Gardenia jasminoides J.Ellis, Camellia sinensis (L.) Kuntze | ACHE,AKT1,ALOX5,BCL2,F2,GSTM1,HAS2,HSP90AA1,JUN,MAPK8,MMP1,PIK3CG,PPARG,PRSS1,PTGS2,RELA,SELE,TNF,VCAM1 |
| MOL000392 | formononetin | Glycyrrhiza uralensis Fisch. ex DC | PTGS2,HSP90AA1,JUN,PPARG,PRSS1,F2,ACHE,IL4,MAPK14,PIM1,SIRT1 |
| MOL004328 | naringenin | Citrus × aurantium f. deliciosa, Glycyrrhiza uralensis Fisch. ex DC | AKT1,BCL2,HSP90AA1,MAPK1,PIK3CG,PPARG,PTGS2,RELA,SOAT1,SOD1 |
| MOL003896 | 7-Methoxy-2-methyl isoflavone | Glycyrrhiza uralensis Fisch. ex DC | PTGS2,HSP90AA1,PPARG,PRSS1,F2,ACHE,MAPK14,PIM1 |
| MOL000354 | isorhamnetin | Glycyrrhiza uralensis Fisch. ex DC | PTGS2,HSP90AA1,PIK3CG,PPARG,PRSS1,F2,ACHE,RELA,NCF1,MAPK14,PIM1 |
| MOL005828 | nobiletin | Citrus × aurantium f. deliciosa | PTGS2,HSP90AA1,BCL2,JUN,PPARG,PRSS1,F2,MAPK8,MMP9,TP53,TIMP1,CREB1,CD163 |
| MOL004991 | 7-Acetoxy-2-methylisoflavone | Glycyrrhiza uralensis Fisch. ex DC | PTGS2,HSP90AA1,PPARG,PRSS1,F2,ACHE,MAPK14 |
| MOL003095 | 5-hydroxy-7-methoxy-2-(3,4,5-trimethoxyphenyl) chromone | Lonicera japonica Thunb, Gardenia jasminoides J.Ellis | F2,HSP90AA1,MAPK14,PPARG,PRSS1,PTGS2 |
| MOL005003 | Licoagrocarpin | Glycyrrhiza uralensis Fisch. ex DC | PTGS2,HSP90AA1,PPARG,PRSS1,F2,ACHE,MAPK14,PIM1 |
| MOL000497 | Licochalcone a | Glycyrrhiza uralensis Fisch. ex DC | PTGS2,HSP90AA1,BCL2,PPARG,RELA,CCND1,MAPK1,MAPK14,PIM1,STAT3,FOSL2 |
| MOL004959 | 1-Methoxyphaseollidin | Glycyrrhiza uralensis Fisch. ex DC | PTGS2,HSP90AA1,PIK3CG,PPARG,PRSS1,F2,MAPK14,PIM1 |
| MOL004978 | 2-[(3R)-8,8-dimethyl-3,4-dihydro-2H-pyrano[6,5-f]chromen-3-yl]-5-methoxyphenol | Glycyrrhiza uralensis Fisch. ex DC | PTGS2,PPARG,PRSS1,ACHE,MAPK14,PIM1 |
| MOL000358 | beta-sitosterol | Panax ginseng C.A. Mey, Lonicera japonica Thunb, Wurfbainia villosa var. villosa, Gardenia jasminoides J.Ellis | BCL2,HSP90AA1,JUN,PIK3CG,PTGS2,TGFB1 |
| MOL000417 | Calycosin | Glycyrrhiza uralensis Fisch. ex DC | PTGS2,HSP90AA1,PPARG,PRSS1,MAPK14,PIM1 |
| MOL005321 | Frutinone A | Panax ginseng C.A. Mey | PTGS2,HSP90AA1,PIK3CG,PPARG,F2,ACHE |
| MOL004828 | Glepidotin A | Glycyrrhiza uralensis Fisch. ex DC | PTGS2,HSP90AA1,PPARG,PRSS1,F2,MAPK14,PIM1 |
| MOL000500 | Vestitol | Glycyrrhiza uralensis Fisch. ex DC | PTGS2,HSP90AA1,PPARG,PRSS1,MAPK14,PIM1 |
| MOL004974 | 3'-Methoxyglabridin | Glycyrrhiza uralensis Fisch. ex DC | PTGS2,HSP90AA1,PPARG,PRSS1,ACHE,MAPK14,PIM1 |
| MOL007245 | 3-Methylkempferol | Gardenia jasminoides J.Ellis | PTGS2,HSP90AA1,PIK3CG,MAPK14 |
| MOL004990 | 7,2',4'-trihydroxy－5-methoxy-3－arylcoumarin | Glycyrrhiza uralensis Fisch. ex DC | PTGS2,HSP90AA1,PPARG,MAPK14,PIM1 |
| MOL003044 | Chryseriol | Lonicera japonica Thunb | PTGS2,HSP90AA1,PIK3CG,PPARG,PRSS1,MAPK14 |
| MOL005000 | Gancaonin G | Glycyrrhiza uralensis Fisch. ex DC | PTGS2,HSP90AA1,PPARG,PRSS1,F2,MAPK14,PIM1 |
| MOL004912 | Glabrone | Glycyrrhiza uralensis Fisch. ex DC | PTGS2,PPARG,PRSS1,F2,ACHE,MAPK14,PIM1 |
| MOL004811 | Glyasperin C | Glycyrrhiza uralensis Fisch. ex DC | PTGS2,HSP90AA1,PPARG,PRSS1,F2,ACHE,MAPK14,PIM1 |
| MOL004835 | Glypallichalcone | Glycyrrhiza uralensis Fisch. ex DC | PTGS2,HSP90AA1,PPARG,MAPK14 |
| MOL004907 | Glyzaglabrin | Glycyrrhiza uralensis Fisch. ex DC | PTGS2,HSP90AA1,PIK3CG,PPARG,PRSS1,MAPK14,PIM1 |
| MOL004957 | HMO | Glycyrrhiza uralensis Fisch. ex DC | PTGS2,PPARG,PRSS1,MAPK14,PIM1 |
| MOL004841 | Licochalcone B | Glycyrrhiza uralensis Fisch. ex DC | PTGS2,HSP90AA1,PPARG,MAPK14,PIM1 |
| MOL004849 | 3-(2,4-dihydroxyphenyl)-8-(1,1-dimethylprop-2-enyl)-7-hydroxy-5-methoxy-coumarin | Glycyrrhiza uralensis Fisch. ex DC | PTGS2,HSP90AA1,PPARG,PRSS1,F2,MAPK14,PIM1 |
| MOL004966 | 3'-Hydroxy-4'-O-Methylglabridin | Glycyrrhiza uralensis Fisch. ex DC | PTGS2,HSP90AA1,PPARG,PRSS1,MAPK14,PIM1 |
| MOL002773 | beta-carotene | Lonicera japonica Thunb | PTGS2,BCL2,JUN,AKT1,MMP1,MMP2,CAV1,MYC,ALB,CTNNB1 |
| MOL004915 | Eurycarpin A | Glycyrrhiza uralensis Fisch. ex DC | PTGS2,HSP90AA1,PPARG,PRSS1,F2,MAPK14,PIM1 |
| MOL004856 | Gancaonin A | Glycyrrhiza uralensis Fisch. ex DC | PTGS2,HSP90AA1,PPARG,PRSS1,F2,ACHE,PIM1 |
| MOL004857 | Gancaonin B | Glycyrrhiza uralensis Fisch. ex DC | PTGS2,HSP90AA1,PPARG,PRSS1,F2,PIM1 |
| MOL004911 | Glabrene | Glycyrrhiza uralensis Fisch. ex DC | PTGS2,HSP90AA1,PPARG,PRSS1,MAPK14,PIM1 |
| MOL004908 | Glabridin | Glycyrrhiza uralensis Fisch. ex DC | PTGS2,PPARG,PRSS1,ACHE,MAPK14,PIM1 |
| MOL004808 | glyasperin B | Glycyrrhiza uralensis Fisch. ex DC | PTGS2,HSP90AA1,PPARG,PRSS1,F2,ACHE,PIM1 |
| MOL005007 | Glyasperins M | Glycyrrhiza uralensis Fisch. ex DC | PTGS2,HSP90AA1,PPARG,PRSS1,ACHE,PIM1 |
| MOL004980 | Inflacoumarin A | Glycyrrhiza uralensis Fisch. ex DC | PTGS2,HSP90AA1,PPARG,PRSS1,F2,PIM1 |
| MOL003656 | Lupiwighteone | Glycyrrhiza uralensis Fisch. ex DC | PTGS2,HSP90AA1,PPARG,PRSS1,F2,MAPK14,PIM1 |
| MOL002565 | Medicarpin | Glycyrrhiza uralensis Fisch. ex DC | PTGS2,HSP90AA1,PIK3CG,PRSS1,PIM1 |
| MOL005016 | Odoratin | Glycyrrhiza uralensis Fisch. ex DC | PTGS2,HSP90AA1,PPARG,PRSS1,MAPK14,PIM1 |
| MOL005017 | Phaseol | Glycyrrhiza uralensis Fisch. ex DC | PTGS2,HSP90AA1,PPARG,F2,MAPK14,PIM1 |
| MOL004961 | Quercetin der. | Glycyrrhiza uralensis Fisch. ex DC | PTGS2,HSP90AA1,PPARG,PRSS1,MAPK14 |
| MOL004891 | shinpterocarpin | Glycyrrhiza uralensis Fisch. ex DC | PTGS2,PIK3CG,PPARG,PRSS1,MAPK14,PIM1 |
| MOL004561 | Sudan III | Gardenia jasminoides J.Ellis | PTGS2,F2,MAPK14,PIM1 |
| MOL004824 | (2S)-6-(2,4-dihydroxyphenyl)-2-(2-hydroxypropan-2-yl)-4-methoxy-2,3-dihydrofuro[3,2-g]chromen-7-one | Glycyrrhiza uralensis Fisch. ex DC | PTGS2,PPARG,PRSS1,F2,ACHE,MAPK14,PIM1 |
| MOL004914 | 1,3-dihydroxy-8,9-dimethoxy-6-benzofurano[3,2-c]chromenone | Glycyrrhiza uralensis Fisch. ex DC | HSP90AA1,PPARG,MAPK14 |
| MOL004866 | 2-(3,4-dihydroxyphenyl)-5,7-dihydroxy-6-(3-methylbut-2-enyl)chromone | Glycyrrhiza uralensis Fisch. ex DC | PTGS2,HSP90AA1,PPARG,PRSS1,F2,PIM1 |
| MOL004863 | 3-(3,4-dihydroxyphenyl)-5,7-dihydroxy-8-(3-methylbut-2-enyl)chromone | Glycyrrhiza uralensis Fisch. ex DC | PTGS2,HSP90AA1,PPARG,PRSS1,F2,MAPK14,PIM1 |
| MOL004864 | 5,7-dihydroxy-3-(4-methoxyphenyl)-8-(3-methylbut-2-enyl)chromone | Glycyrrhiza uralensis Fisch. ex DC | PTGS2,HSP90AA1,PPARG,PRSS1,MAPK14,PIM1 |
| MOL000787 | Fumarine | Panax ginseng C.A. Mey | PTGS2,HSP90AA1 |
| MOL005344 | Ginsenoside-Rh2 | Panax ginseng C.A. Mey | PTGS2,TNF,NFKBIA,IL1B,IFNG |
| MOL004810 | glyasperin F | Glycyrrhiza uralensis Fisch. ex DC | PTGS2,HSP90AA1,PPARG,PRSS1,MAPK14,PIM1 |
| MOL005008 | Glycyrrhiza flavonol A | Glycyrrhiza uralensis Fisch. ex DC | PTGS2,HSP90AA1,PRSS1,ACHE,PIM1 |
| MOL004814 | Isotrifoliol | Glycyrrhiza uralensis Fisch. ex DC | PTGS2,HSP90AA1,PIK3CG,MAPK14,PIM1 |
| MOL005012 | Licoagroisoflavone | Glycyrrhiza uralensis Fisch. ex DC | PTGS2,PPARG,PRSS1,F2,MAPK14,PIM1 |
| MOL004885 | licoisoflavanone | Glycyrrhiza uralensis Fisch. ex DC | PTGS2,HSP90AA1,PPARG,PRSS1,ACHE,PIM1 |
| MOL004883 | Licoisoflavone | Glycyrrhiza uralensis Fisch. ex DC | PTGS2,HSP90AA1,PPARG,PRSS1,F2,MAPK14,PIM1 |
| MOL004833 | Phaseolinisoflavan | Glycyrrhiza uralensis Fisch. ex DC | PTGS2,PPARG,PRSS1,ACHE,MAPK14,PIM1 |
| MOL004827 | Semilicoisoflavone B | Glycyrrhiza uralensis Fisch. ex DC | PTGS2,HSP90AA1,PPARG,PRSS1,F2,ACHE |
| MOL004941 | (2R)-7-hydroxy-2-(4-hydroxyphenyl)chroman-4-one | Glycyrrhiza uralensis Fisch. ex DC | PTGS2,HSP90AA1,PIK3CG |
| MOL004815 | (E)-1-(2,4-dihydroxyphenyl)-3-(2,2-dimethylchromen-6-yl)prop-2-en-1-one | Glycyrrhiza uralensis Fisch. ex DC | PTGS2,PPARG,MAPK14,PIM1 |
| MOL004898 | (E)-3-[3,4-dihydroxy-5-(3-methylbut-2-enyl)phenyl]-1-(2,4-dihydroxyphenyl)prop-2-en-1-one | Glycyrrhiza uralensis Fisch. ex DC | PTGS2,HSP90AA1,PPARG,MAPK14,PIM1 |
| MOL004913 | 1,3-dihydroxy-9-methoxy-6-benzofurano[3,2-c]chromenone | Glycyrrhiza uralensis Fisch. ex DC | HSP90AA1,PPARG,MAPK14 |
| MOL003111 | Centauroside_qt | Lonicera japonica Thunb | HSP90AA1,PRSS1,F2 |
| MOL005020 | dehydroglyasperins C | Glycyrrhiza uralensis Fisch. ex DC | PTGS2,HSP90AA1,PPARG,PRSS1,MAPK14,PIM1 |
| MOL001792 | DFV | Glycyrrhiza uralensis Fisch. ex DC | PTGS2,HSP90AA1,PIK3CG |
| MOL004910 | Glabranin | Glycyrrhiza uralensis Fisch. ex DC | PTGS2,HSP90AA1 |
| MOL003648 | Inermin | Panax ginseng C.A. Mey | PTGS2,HSP90AA1,PIK3CG,PRSS1 |
| MOL001484 | Inermine | Glycyrrhiza uralensis Fisch. ex DC | PTGS2,HSP90AA1,PIK3CG,PRSS1 |
| MOL004949 | Isolicoflavonol | Glycyrrhiza uralensis Fisch. ex DC | PTGS2,HSP90AA1,PPARG,PRSS1,F2,PIM1 |
| MOL000239 | Jaranol | Glycyrrhiza uralensis Fisch. ex DC | PTGS2,HSP90AA1,PRSS1 |
| MOL004820 | kanzonols W | Glycyrrhiza uralensis Fisch. ex DC | PTGS2,PPARG,PRSS1,MAPK14,PIM1 |
| MOL004848 | licochalcone G | Glycyrrhiza uralensis Fisch. ex DC | PTGS2,HSP90AA1,PPARG,MAPK14,PIM1 |
| MOL004884 | Licoisoflavone B | Glycyrrhiza uralensis Fisch. ex DC | PTGS2,PPARG,PRSS1,F2,ACHE,PIM1 |
| MOL005384 | suchilactone | Panax ginseng C.A. Mey | PTGS2,HSP90AA1 |
| MOL004805 | (2S)-2-[4-hydroxy-3-(3-methylbut-2-enyl)phenyl]-8,8-dimethyl-2,3-dihydropyrano[2,3-f]chromen-4-one | Glycyrrhiza uralensis Fisch. ex DC | PTGS2,PPARG,MAPK14,PIM1 |
| MOL004945 | (2S)-7-hydroxy-2-(4-hydroxyphenyl)-8-(3-methylbut-2-enyl)chroman-4-one | Glycyrrhiza uralensis Fisch. ex DC | PTGS2,HSP90AA1 |
| MOL005100 | 5,7-dihydroxy-2-(3-hydroxy-4-methoxyphenyl) chroman-4-one | Citrus × aurantium f. deliciosa | PTGS2,HSP90AA1,PIK3CG |
| MOL001941 | Ammidin | Gardenia jasminoides J.Ellis | PTGS2,PIK3CG,F2 |
| MOL000492 | Catechin | Camellia sinensis (L.) Kuntze | PTGS2,HSP90AA1,HAS2 |
| MOL002914 | Eriodyctiol (flavanone) | Lonicera japonica Thunb | PTGS2,HSP90AA1,PIK3CG |
| MOL005356 | Girinimbin | Panax ginseng C.A. Mey | PTGS2,PIK3CG |
| MOL004829 | Glepidotin B | Glycyrrhiza uralensis Fisch. ex DC | PTGS2,HSP90AA1 |
| MOL004879 | Glycyrin | Glycyrrhiza uralensis Fisch. ex DC | PTGS2,PPARG,PRSS1,F2,PIM1 |
| MOL002311 | Glycyrol | Glycyrrhiza uralensis Fisch. ex DC | PTGS2,PPARG,F2,MAPK14,PIM1 |
| MOL004948 | Isoglycyrol | Glycyrrhiza uralensis Fisch. ex DC | PTGS2,PIM1 |
| MOL004882 | Licocoumarone | Glycyrrhiza uralensis Fisch. ex DC | HSP90AA1 |
| MOL004904 | licopyranocoumarin | Glycyrrhiza uralensis Fisch. ex DC | PTGS2,PPARG,PRSS1,F2,ACHE,PIM1 |
| MOL000449 | Stigmasterol | Panax ginseng C.A. Mey, Lonicera japonica Thunb, Wurfbainia villosa var. villosa, Gardenia jasminoides J.Ellis | PTGS2,PLAU |
| MOL005318 | Dianthramine | Panax ginseng C.A. Mey | PTGS2,HSP90AA1 |
| MOL005001 | Gancaonin H | Glycyrrhiza uralensis Fisch. ex DC | PTGS2,HSP90AA1,PRSS1,PIM1 |
| MOL003117 | Ioniceracetalides B_qt | Lonicera japonica Thunb | PTGS2,F2 |
| MOL004855 | Licoricone | Glycyrrhiza uralensis Fisch. ex DC | PTGS2,PPARG,PRSS1,F2,PIM1 |
| MOL003006 | (-)-(3R,8S,9R,9aS, 10aS)-9-ethenyl-8-(beta-D-glucopyranosyloxy)-2,3,9,9a,10,10a-hexahydro-5-oxo-5H,8H-pyrano[4,3-d]oxazolo[3,2-a]pyridine-3-carboxylic acid_qt | Lonicera japonica Thunb | PTGS2 |
| MOL004924 | (-)-Medicocarpin | Glycyrrhiza uralensis Fisch. ex DC | PTGS2,ACHE |
| MOL004989 | 6-prenylated eriodictyol | Glycyrrhiza uralensis Fisch. ex DC | PTGS2,HSP90AA1 |
| MOL004838 | 8-(6-hydroxy-2-benzofuranyl)-2,2-dimethyl-5-chromenol | Glycyrrhiza uralensis Fisch. ex DC | PTGS2,HSP90AA1,PIK3CG |
| MOL004993 | 8-prenylated eriodictyol | Glycyrrhiza uralensis Fisch. ex DC | PTGS2,HSP90AA1 |
| MOL005320 | arachidonate | Panax ginseng C.A. Mey | PTGS2 |
| MOL005815 | Citromitin | Citrus × aurantium f. deliciosa | PTGS2,HSP90AA1 |
| MOL000296 | hederagenin | Poria cocos | PTGS2 |
| MOL004988 | Kanzonol F | Glycyrrhiza uralensis Fisch. ex DC | PTGS2,PIM1 |
| MOL004903 | liquiritin | Glycyrrhiza uralensis Fisch. ex DC | PTGS2,SOD1 |
| MOL001494 | Mandenol | Lonicera japonica Thunb, Gardenia jasminoides J.Ellis | PTGS2 |
| MOL000569 | m-Digallic acid | Camellia sinensis (L.) Kuntze | PTGS2,HSP90AA1 |
| MOL003014 | secologanic dibutylacetal_ qt | Lonicera japonica Thunb | PTGS2,F2 |
| MOL004935 | Sigmoidin-B | Glycyrrhiza uralensis Fisch. ex DC | PTGS2,HSP90AA1 |
| MOL001406 | crocetin | Gardenia jasminoides J.Ellis | PTGS2,VCAM1 |
| MOL005018 | Xambioona | Glycyrrhiza uralensis Fisch. ex DC | PTGS2,PIM1 |
| MOL001942 | isoimperatorin | Gardenia jasminoides J.Ellis | PTGS2 |
| MOL004806 | euchrenone | Glycyrrhiza uralensis Fisch. ex DC | PTGS2 |

Table S4 The possible targets of Ginseng honeysuckle superfine powdered tea against pulmonary fibrosis

| NO. | Target name | Gene symbol | Degree | Betweenness | Closeness centrality |
| --- | --- | --- | --- | --- | --- |
| 1 | prostaglandin-endoperoxide synthase 2 | PTGS2 | 113 | 0.324 | 0.678 |
| 2 | heat shock protein 90 alpha family class A member 1 | HSP90AA1 | 80 | 0.131 | 0.558 |
| 3 | Peroxisome proliferator-activated receptor gamma | PPARG | 69 | 0.084 | 0.527 |
| 4 | Proto-oncogene serine/threonine-protein kinase Pim-1 | PIM1 | 63 | 0.051 | 0.511 |
| 5 | Trypsin-1 | PRSS1 | 61 | 0.057 | 0.506 |
| 6 | Mitogen-activated protein kinase 14 | MAPK14 | 52 | 0.036 | 0.485 |
| 7 | Prothrombin | F2 | 41 | 0.033 | 0.461 |
| 8 | Acetylcholinesterase | ACHE | 25 | 0.015 | 0.431 |
| 9 | Phosphatidylinositol-4,5-bisphosphate 3-kinase catalytic subunit gamma isoform | PIK3CG | 24 | 0.021 | 0.429 |
| 10 | Apoptosis regulator Bcl-2 | BCL2 | 9 | 0.003 | 0.404 |
| 11 | Transcription factor AP-1 | JUN | 9 | 0.003 | 0.404 |
| 12 | Transcription factor p65 | RELA | 8 | 0.002 | 0.402 |
| 13 | RAC-alpha serine/threonine-protein kinase | AKT1 | 7 | 0.001 | 0.401 |
| 14 | Tumor necrosis factor | TNF | 6 | 0.001 | 0.399 |
| 15 | Interstitial collagenase | MMP1 | 6 | 9.21E-04 | 0.399 |
| 16 | Mitogen-activated protein kinase 1 | MAPK1 | 6 | 0.001 | 0.399 |
| 17 | NF-kappa-B inhibitor alpha | NFKBIA | 5 | 0.001 | 0.398 |
| 18 | G1/S-specific cyclin-D1 | CCND1 | 5 | 6.20E-04 | 0.398 |
| 19 | 72 kDa type IV collagenase | MMP2 | 5 | 6.56E-04 | 0.398 |
| 20 | Cellular tumor antigen p53 | TP53 | 5 | 5.54E-04 | 0.398 |
| 21 | Matrix metalloproteinase-9 | MMP9 | 5 | 5.54E-04 | 0.398 |
| 22 | Superoxide dismutase [Cu-Zn] | SOD1 | 5 | 0.002 | 0.398 |
| 23 | Mitogen-activated protein kinase 8 | MAPK8 | 4 | 4.33E-04 | 0.396 |
| 24 | Vascular cell adhesion protein 1 | VCAM1 | 4 | 0.002 | 0.396 |
| 25 | Hyaluronan synthase 2 | HAS2 | 4 | 0.001 | 0.396 |
| 26 | Urokinase-type plasminogen activator | PLAU | 4 | 0.002 | 0.396 |
| 27 | Interferon gamma | IFNG | 4 | 7.48E-04 | 0.396 |
| 28 | Cyclin-dependent kinase inhibitor 1 | CDKN1A | 4 | 2.18E-04 | 0.396 |
| 29 | Interleukin-6 | IL6 | 4 | 2.18E-04 | 0.396 |
| 30 | Receptor tyrosine-protein kinase erbB-2 | ERBB2 | 4 | 2.18E-04 | 0.396 |
| 31 | Caveolin-1 | CAV1 | 4 | 4.72E-04 | 0.396 |
| 32 | Transforming growth factor beta-1 | TGFB1 | 3 | 4.74E-04 | 0.395 |
| 33 | E-selectin | SELE | 3 | 1.60E-04 | 0.395 |
| 34 | Arachidonate 5-lipoxygenase | ALOX5 | 3 | 1.60E-04 | 0.395 |
| 35 | Glutathione S-transferase Mu 1 | GSTM1 | 3 | 1.60E-04 | 0.395 |
| 36 | Interleukin-1 beta | IL1B | 3 | 5.81E-04 | 0.395 |
| 37 | E3 ubiquitin-protein ligase Mdm2 | MDM2 | 3 | 1.28E-04 | 0.395 |
| 38 | Amyloid beta A4 protein | APP | 3 | 1.28E-04 | 0.395 |
| 39 | Interleukin-2 | IL2 | 3 | 1.21E-04 | 0.395 |
| 40 | Interleukin-4 | IL4 | 3 | 3.08E-04 | 0.395 |
| 41 | Stromelysin-1 | MMP3 | 3 | 8.03E-05 | 0.395 |
| 42 | Proto-oncogene c-Fos | FOS | 3 | 8.03E-05 | 0.395 |
| 43 | Hypoxia-inducible factor 1-alpha | HIF1A | 3 | 8.03E-05 | 0.395 |
| 44 | 78 kDa glucose-regulated protein | HSPA5 | 3 | 8.03E-05 | 0.395 |
| 45 | Myc proto-oncogene protein | MYC | 3 | 3.06E-04 | 0.395 |
| 46 | Neutrophil cytosol factor 1 | NCF1 | 3 | 3.07E-04 | 0.395 |
| 47 | Metalloproteinase inhibitor 1 | TIMP1 | 3 | 2.46E-04 | 0.395 |
| 48 | Cyclic AMP-responsive element-binding protein 1 | CREB1 | 3 | 2.46E-04 | 0.395 |
| 49 | Signal transducer and activator of transcription 3 | STAT3 | 3 | 2.92E-04 | 0.395 |
| 50 | Fos-related antigen 2 | FOSL2 | 3 | 2.92E-04 | 0.395 |
| 51 | Hepatocyte growth factor receptor | MET | 2 | 6.59E-05 | 0.393 |
| 52 | Small inducible cytokine A2 | CCL2 | 2 | 2.19E-05 | 0.393 |
| 53 | Interleukin-8 | CXCL8 | 2 | 2.19E-05 | 0.393 |
| 54 | Heat shock protein beta-1 | HSPB1 | 2 | 2.19E-05 | 0.393 |
| 55 | Thrombomodulin | THBD | 2 | 2.19E-05 | 0.393 |
| 56 | Plasminogen activator inhibitor 1 | SERPINE1 | 2 | 2.19E-05 | 0.393 |
| 57 | Phosphatidylinositol-3,4,5-trisphosphate 3-phosphatase and dual-specificity protein phosphatase PTEN | PTEN | 2 | 2.19E-05 | 0.393 |
| 58 | Interleukin-1 alpha | IL1A | 2 | 2.19E-05 | 0.393 |
| 59 | Nuclear factor erythroid 2-related factor 2 | NFE2L2 | 2 | 2.19E-05 | 0.393 |
| 60 | C-X-C motif chemokine 11 | CXCL11 | 2 | 2.19E-05 | 0.393 |
| 61 | Heat shock factor protein 1 | HSF1 | 2 | 2.19E-05 | 0.393 |
| 62 | C-X-C motif chemokine 10 | CXCL10 | 2 | 2.19E-05 | 0.393 |
| 63 | Osteopontin | SPP1 | 2 | 2.19E-05 | 0.393 |
| 64 | Insulin-like growth factor-binding protein 3 | IGFBP3 | 2 | 2.19E-05 | 0.393 |
| 65 | Insulin-like growth factor II | IGF2 | 2 | 2.19E-05 | 0.393 |
| 66 | Receptor tyrosine-protein kinase erbB-3 | ERBB3 | 2 | 2.19E-05 | 0.393 |
| 67 | Serum albumin | ALB | 2 | 1.87E-04 | 0.393 |
| 68 | Catenin beta-1 | CTNNB1 | 2 | 1.87E-04 | 0.393 |
| 69 | Sterol O-acyltransferase 1 | SOAT1 | 2 | 1.78E-04 | 0.393 |
| 70 | Scavenger receptor cysteine-rich type 1 protein M130 | CD163 | 2 | 1.43E-04 | 0.393 |
| 71 | NAD-dependent deacetylase sirtuin-1 | SIRT1 | 2 | 2.01E-04 | 0.393 |
| 72 | Serine/threonine-protein kinase TBK1 | TBK1 | 2 | 2.37E-05 | 0.393 |
| 73 | Angiotensinogen | AGT | 2 | 2.37E-05 | 0.393 |
| 74 | Basic fibroblast growth factor receptor 1 | FGFR1 | 2 | 2.37E-05 | 0.393 |
| 75 | Early growth response protein 1 | EGR1 | 2 | 2.37E-05 | 0.393 |
| 76 | Fibroblast growth factor 10 | FGF10 | 2 | 2.37E-05 | 0.393 |
| 77 | Beta-type platelet-derived growth factor receptor | PDGFRB | 2 | 2.37E-05 | 0.393 |
| 78 | Matrix metalloproteinase-14 | MMP14 | 2 | 2.37E-05 | 0.393 |
| 79 | Bcl-2-binding component 3 | BBC3 | 2 | 2.37E-05 | 0.393 |
| 80 | Transcription factor jun-D | JUND | 2 | 2.37E-05 | 0.393 |
| 81 | Matrilysin | MMP7 | 2 | 2.37E-05 | 0.393 |
| 82 | Endothelin-1 | EDN1 | 2 | 2.37E-05 | 0.393 |
| 83 | Vimentin | VIM | 2 | 2.37E-05 | 0.393 |
| 84 | Forkhead box protein O1 | FOXO1 | 2 | 2.37E-05 | 0.393 |
| 85 | Tumor necrosis factor receptor superfamily member 6 | FAS | 2 | 2.37E-05 | 0.393 |
| 86 | CCAAT/enhancer-binding protein alpha | CEBPA | 2 | 2.37E-05 | 0.393 |
| 87 | Toll-like receptor 4 | TLR4 | 2 | 2.37E-05 | 0.393 |
| 88 | Transcription factor Sp1 | SP1 | 2 | 2.37E-05 | 0.393 |
| 89 | 3-oxo-5-alpha-steroid 4-dehydrogenase 2 | SRD5A2 | 2 | 2.37E-05 | 0.393 |
| 90 | Interleukin-6 receptor subunit beta | IL6ST | 2 | 2.37E-05 | 0.393 |
| 91 | Mitogen-activated protein kinase 7 | MAPK7 | 2 | 2.37E-05 | 0.393 |
| 92 | Mucin-5AC | MUC5AC | 2 | 2.37E-05 | 0.393 |

Table S5 Serum migrant compounds and related targets of Ginseng honeysuckle superfine

powdered tea against pulmonary fibrosis

| Mol ID | Ingredient | Medicine | Target for pulmonary fibrosis |
| --- | --- | --- | --- |
| MOL000511 | Ursolic Acid | Glycyrrhiza uralensis Fisch. ex DC, Lonicera japonica Thunb, Gardenia jasminoides J.Ellis | PLAU,STAT3,VEGFA,CCND1,FOS,MMP2,MMP9,TNF,JUN,IL6,TP53,MAPK8,PTGS2,MMP1,MMP3,MMP10,IL1B |
| MOL006505 | (-)-Epicatechin | Camellia sinensis (L.) Kuntze | PTGS2,HSP90AA1,AKT1,PLAU,TNF,JUN,IL6,CCL2,IL1A,ACE,HAS2 |
| MOL001789 | Isoliquiritigenin | Glycyrrhiza uralensis Fisch. ex DC | PPARG,PTGS2,MAPK14,HSP90AA1,PIK3CG,FOS,VCAM1,JAK2 |
| MOL011400 | Ginsenoside Rf | Panax ginseng C.A. Mey | TNF,PTGS2,IL1B,IFNG,IL4 |
| MOL001792 | Liquiritigenin | Glycyrrhiza uralensis Fisch. ex DC | PTGS2,HSP90AA1,PIK3CG |
| MOL002844 | Pinocembrin | Glycyrrhiza uralensis Fisch. ex DC | PTGS2,HSP90AA1,PIK3CG |
| MOL000492 | Catechin | Camellia sinensis (L.) Kuntze | PTGS2,HSP90AA1,HAS2 |
| MOL004804 | Glycyrrhetinic acid | Glycyrrhiza uralensis Fisch. ex DC | CTNNB1,JUP |
| MOL001648 | Genipin | Gardenia jasminoides J.Ellis | PTGS2 |
| MOL000263 | Oleanolic acid | Glycyrrhiza uralensis Fisch. ex DC, Lonicera japonica Thunb, Gardenia jasminoides J.Ellis | HMOX1 |
| MOL001641 | Methyl linoleate | Lonicera japonica Thunb, Gardenia jasminoides J.Ellis | PTGS2 |
| MOL006527 | Theobromine | Camellia sinensis (L.) Kuntze | PTGS2 |

Table S6. Pathway enrichment analysis of differential expression proteins of the lung

in the pulmonary fibrosis mice

| Pathway ID | Pathway name | Identified components | *P* value | Count |
| --- | --- | --- | --- | --- |
| mmu04142 | Lysosome | Ctsd,Ctsh,Ctss,Ctsb,Scarb2,Npc2,Tcirg1,Lgmn,Gusb,Atp6v0d1,Ctsz,Hexb,Ctsa,Ctsl,Atp6v0c,Tpp1,Lamp2,Lamp1,Cd63,Ctsk,Acp2,Gla,Acp5,Ap1s2,Neu1 | 2.54E-13 | 25 |
| mmu00380 | Tryptophan metabolism | Acat1,Cat,Maob,Aox3,Inmt,Dld,Aldh7a1,Hadh,Aox1,Kyat3,Cyp1a1 | 3.58E-07 | 11 |
| mmu00280 | Valine, leucine and isoleucine degradation | Acaa2,Acat1,Pcca,Aox3,Pccb,Dld,Aldh7a1,Mccc2,Hadh,Mccc1,Aox1 | 9.54E-07 | 11 |
| mmu00640 | Propanoate metabolism | Suclg2,Pcca,Pccb,Ldhb,Acss1,Dld,Suclg1,Acss2 | 2.97E-06 | 8 |
| mmu01200 | Carbon metabolism | Eno1,Acat1,Cat,Idh2,Suclg2,Pcca,Pccb,Pfkl,Acss1,Dld,Suclg1,Prps2,Acss2,Psat1,Hkdc1 | 3.88E-06 | 15 |
| mmu00330 | Arginine and proline metabolism | Maob,Aldh7a1,Aldh4a1,Ckmt2,P4ha1,Ckmt1,Aldh18a1,P4ha2,Arg2,Pycrl | 4.45E-06 | 10 |
| mmu00630 | Glyoxylate and dicarboxylate metabolism | Acat1,Cat,Pcca,Pccb,Acss1,Dld,Acss2 | 4.06E-05 | 7 |
| mmu05410 | Hypertrophic cardiomyopathy | Myh6,Atp2a2,Lama2,Atp2a3,Itga6,Prkaa1,Tnnc1,Tnnt2,Tnni3,Itgb6 | 4.42E-04 | 10 |
| mmu01230 | Biosynthesis of amino acids | Eno1,Idh2,Pfkl,Prps2,Aldh18a1,Psat1,Arg2,Pycrl,Otc | 6.51E-04 | 9 |
| mmu00010 | Glycolysis / Gluconeogenesis | Eno1,Ldhb,Pfkl,Acss1,Dld,Aldh7a1,Acss2,Hkdc1 | 9.47E-04 | 8 |
| mmu00260 | Glycine, serine and threonine metabolism | Maob,Dld,Aldh7a1,Psat1,Chdh,Gcat | 1.24E-03 | 6 |
| mmu04216 | Ferroptosis | Cp,Acsl1,Ftl1,Fth1,Steap3,Prnp | 1.24E-03 | 6 |
| mmu00982 | Drug metabolism - cytochrome P450 | Gstm2,Maob,Aox3,Gstm1,Aox1,Gstt3,Ugt1a7c,Fmo5 | 1.39E-03 | 8 |
| mmu00450 | Selenocompound metabolism | Inmt,Txnrd2,Kyat3,Sepsecs | 1.49E-03 | 4 |
| mmu00980 | Metabolism of xenobiotics by cytochrome P450 | Cbr2,Gstm2,Cyp2f2,Gstm1,Dhdh,Gstt3,Ugt1a7c,Cyp1a1 | 1.67E-03 | 8 |
| mmu00620 | Pyruvate metabolism | Acat1,Ldhb,Acss1,Dld,Aldh7a1,Acss2 | 2.06E-03 | 6 |
| mmu05414 | Dilated cardiomyopathy | Myh6,Atp2a2,Lama2,Atp2a3,Itga6,Tnnc1,Tnnt2,Tnni3,Itgb6 | 2.26E-03 | 9 |
| mmu00020 | Citrate cycle (TCA cycle) | Acly,Idh2,Suclg2,Dld,Suclg1 | 2.65E-03 | 5 |
| mmu04145 | Phagosome | H2-D1,H2-K1,Ctss,Tcirg1,H2-Q4,Atp6v0d1,Ctsl,Cyba,Atp6v0c,Fcgr2b,Lamp2,Lamp1,Pikfyve | 3.85E-03 | 13 |
| mmu00040 | Pentose and glucuronate interconversions | Gusb,Dhdh,Ugt1a7c,Akr1b7,Crppa | 4.48E-03 | 5 |
| mmu00071 | Fatty acid degradation | Acaa2,Acat1,Acsl1,Eci1,Aldh7a1,Hadh | 4.86E-03 | 6 |
| mmu00983 | Drug metabolism - other enzymes | Ces1d,Gstm2,Gstm1,Hprt,Gusb,Gstt3,Ugt1a7c,Ces1f | 7.02E-03 | 8 |
| mmu04915 | Estrogen signaling pathway | Krt19,Krt18,Ctsd,Fkbp4,Fkbp5,Mmp2,Krt23,Krt42,Krt24,Sp1 | 7.96E-03 | 10 |
| mmu04210 | Apoptosis | Ctsd,Ctsh,Ctss,Ctsb,Parp3,Dab2ip,Ctsz,Ctsl,Ctsk,Il3ra | 8.80E-03 | 10 |
| mmu00860 | Porphyrin metabolism | Cp,Alad,Gusb,Ugt1a7c,Uros | 9.63E-03 | 5 |
| mmu00650 | Butanoate metabolism | Acat1,Hadh,Aldh5a1,Acsm1 | 9.85E-03 | 4 |
| mmu04670 | Leukocyte transendothelial migration | Afdn,Cdh5,Pxn,Plcg1,Prkca,Ocln,Mmp2,Cyba,Cldn5 | 1.01E-02 | 9 |
| mmu00062 | Fatty acid elongation | Acaa2,Acot2,Hadh,Hacd3 | 1.12E-02 | 4 |
| mmu01212 | Fatty acid metabolism | Fasn,Acaa2,Acat1,Acsl1,Hadh,Hacd3 | 1.14E-02 | 6 |
| mmu05418 | Fluid shear stress and atherosclerosis | Gstm2,Gstm1,Cdh5,Prkaa1,Thbd,Mmp2,Ctsl,Gstt3,Cyba,Kdr | 1.53E-02 | 10 |
| mmu00052 | Galactose metabolism | Pfkl,Akr1b7,Hkdc1,Gla | 1.57E-02 | 4 |
| mmu04146 | Peroxisome | Cat,Idh2,Prdx5,Acsl1,Sod1,Decr2,Pxmp4 | 1.60E-02 | 7 |
| mmu04260 | Cardiac muscle contraction | Myh6,Atp2a2,Atp2a3,Myl4,Tnnc1,Tnnt2,Tnni3 | 1.70E-02 | 7 |
| mmu05230 | Central carbon metabolism in cancer | Idh2,Ldhb,Pfkl,Kit,Hkdc1,Pdk1 | 1.86E-02 | 6 |
| mmu04612 | Antigen processing and presentation | H2-D1,H2-K1,Ctss,Ctsb,H2-Q4,Lgmn,,Ctsl | 2.01E-02 | 7 |
| mmu04666 | Fc gamma R-mediated phagocytosis | Plcg1,Arf6,Prkca,Asap2,Plpp3,Fcgr2b,Amph | 2.24E-02 | 7 |
| mmu00600 | Sphingolipid metabolism | Hexb,Plpp3,Gla,Degs1,Neu1 | 2.41E-02 | 5 |
| mmu05412 | Arrhythmogenic right ventricular cardiomyopathy | Atp2a2,Lama2,Atp2a3,Jup,Itga6,Itgb6 | 3.01E-02 | 6 |
| mmu04621 | NOD-like receptor signaling pathway | Dnm1l,Gbp2,Ctsb,Gbp7,Pkn2,Mavs,Cyba,Oas3,Pstpip1,Tyk2,Ripk2,Traf3 | 3.05E-02 | 12 |
| mmu04919 | Thyroid hormone signaling pathway | Myh6,Atp2a2,Atp2a3,Pfkl,Plcg1,Prkca,Rxrb,Plcd3 | 3.07E-02 | 8 |
| mmu04370 | VEGF signaling pathway | Pxn,Plcg1,Prkca,Ppp3cc,Kdr | 3.17E-02 | 5 |
| mmu04144 | Endocytosis | Ehd4,H2-D1,H2-K1,Arf6,Vps26b,H2-Q4,Asap2,Snx4,Arap3,Agap3,Wwp1,Vps4a,Chmp2b,Amph | 3.97E-02 | 14 |
| mmu04514 | Cell adhesion molecules | Itga6,Alcam,Cdh5,H2-D1,H2-K1,H2-Q4,Ocln,Ptprm,Cldn5,Negr1 | 4.10E-02 | 10 |
| mmu05150 | Staphylococcus aureus infection | Krt19,Krt18,Krt23,Krt42,Krt24,Fcgr2b,C1qa,C3ar1 | 4.25E-02 | 8 |
| mmu00561 | Glycerolipid metabolism | Aldh7a1,Plpp3,Akr1b7,Gla,Lpin3 | 4.31E-02 | 5 |
| mmu01240 | Biosynthesis of cofactors | Dld,Alad,Gusb,Ugt1a7c,Dhrs3,Psat1,Dhfr,Tpk1,Uros | 4.36E-02 | 9 |
| mmu04261 | Adrenergic signaling in cardiomyocytes | Myh6,Atp2a2,Atp2a3,Scn7a,Myl4,Prkca,Tnnc1,Tnnt2,Tnni3 | 4.36E-02 | 9 |
| mmu04966 | Collecting duct acid secretion | Tcirg1,Atp6v0d1,Atp6v0c | 4.83E-02 | 3 |
| mmu05152 | Tuberculosis | Ctsd,Ctss,Tcirg1,Atp6v0d1,Atp6v0c,Fcgr2b,Lamp2,Ppp3cc,Lamp1,Ripk2 | 4.96E-02 | 10 |

Table S7. Pathway enrichment analysis of differential expression proteins of the lung in the pulmonary fibrosis mice as reversed by Ginseng honeysuckle superfine powdered tea

| Pathway ID | Pathway name | Identified components | *P* value | Count |
| --- | --- | --- | --- | --- |
| mmu04216 | Ferroptosis | Fth1 | 0.0393 | 1 |
| mmu00565 | Ether lipid metabolism | Chpt1 | 0.0471 | 1 |
| mmu00520 | Amino sugar and nucleotide sugar metabolism | Npl | 0.0499 | 1 |

Table S8. Pathway enrichment analysis of Ginseng honeysuckle superfine powdered tea and pulmonary fibrosis co-targeted genes

| Pathway ID | Pathway name | Identified components | *P* value | Count |
| --- | --- | --- | --- | --- |
| hsa04620 | Toll-like receptor signaling pathway | AKT1,CXCL10,CXCL11,CXCL8,FOS,IL1B,IL6,JUN,MAPK1,MAPK14,MAPK8,NFKBIA,PIK3CG,RELA,SPP1,TBK1,TLR4,TNF | 1.24E-15 | 18 |
| hsa04380 | Osteoclast differentiation | AKT1,CREB1,FOS,FOSL2,IFNG,IL1A,IL1B,JUN,JUND,MAPK1,MAPK14,MAPK8,NCF1,NFKBIA,PIK3CG,PPARG,RELA,TGFB1,TNF | 5.16E-15 | 19 |
| hsa04621 | NOD-like receptor signaling pathway | CCL2,CXCL8,HSP90AA1,IL1B,IL6,MAPK1,MAPK14,MAPK8,NFKBIA,RELA,TNF | 3.55E-10 | 11 |
| hsa04010 | MAPK signaling pathway | AKT1,FAS,FGF10,FGFR1,FOS,HSPB1,IL1A,IL1B,JUN,JUND,MAPK1,MAPK14,MAPK7,MAPK8,MYC,PDGFRB,RELA,TGFB1,TNF,TP53 | 4.37E-10 | 20 |
| hsa04660 | T cell receptor signaling pathway | AKT1,FOS,IFNG,IL2,IL4,JUN,MAPK1,MAPK14,NFKBIA,PIK3CG,RELA,TNF | 2.53E-08 | 12 |
| hsa05222 | Small cell lung cancer | AKT1,BCL2,CCND1,MYC,NFKBIA,PIK3CG,PTEN,PTGS2,RELA,TP53 | 2.35E-07 | 10 |
| hsa04210 | Apoptosis | AKT1,BCL2,FAS,IL1A,IL1B,NFKBIA,PIK3CG,RELA,TNF,TP53 | 3.64E-07 | 10 |
| hsa04115 | p53 signaling pathway | BBC3,CCND1,CDKN1A,FAS,IGFBP3,MDM2,PTEN,SERPINE1,TP53 | 4.00E-07 | 9 |
| hsa05020 | Prion diseases | EGR1,HSPA5,IL1A,IL1B,IL6,MAPK1,SOD1 | 5.10E-07 | 7 |
| hsa04510 | Focal adhesion | AKT1,BCL2,CAV1,CCND1,CTNNB1,ERBB2,JUN,MAPK1,MAPK8,MET,PDGFRB,PIK3CG,PTEN,SPP1 | 5.72E-07 | 14 |
| hsa04060 | Cytokine-cytokine receptor interaction | CCL2,CXCL10,CXCL11,CXCL8,FAS,IFNG,IL1A,IL1B,IL2,IL4,IL6,IL6ST,MET,PDGFRB,TGFB1,TNF | 6.07E-07 | 16 |
| hsa04722 | Neurotrophin signaling pathway | AKT1,BCL2,JUN,MAPK1,MAPK14,MAPK7,MAPK8,NFKBIA,PIK3CG,RELA,TP53 | 1.30E-06 | 11 |
| hsa05332 | Graft-versus-host disease | FAS,IFNG,IL1A,IL1B,IL2,IL6,TNF | 1.82E-06 | 7 |
| hsa04012 | ErbB signaling pathway | AKT1,CDKN1A,ERBB2,ERBB3,JUN,MAPK1,MAPK8,MYC,PIK3CG | 2.95E-06 | 9 |
| hsa04622 | RIG-I-like receptor signaling pathway | CXCL10,CXCL8,MAPK14,MAPK8,NFKBIA,RELA,TBK1,TNF | 5.70E-06 | 8 |
| hsa04630 | Jak-STAT signaling pathway | AKT1,CCND1,IFNG,IL2,IL4,IL6,IL6ST,MYC,PIK3CG,PIM1,STAT3 | 9.21E-06 | 11 |
| hsa04940 | Type I diabetes mellitus | FAS,IFNG,IL1A,IL1B,IL2,TNF | 3.41E-05 | 6 |
| hsa05120 | Epithelial cell signaling in Helicobacter pylori infection | CXCL8,JUN,MAPK14,MAPK8,MET,NFKBIA,RELA | 4.12E-05 | 7 |
| hsa04062 | Chemokine signaling pathway | AKT1,CCL2,CXCL10,CXCL11,CXCL8,MAPK1,NCF1,NFKBIA,PIK3CG,RELA,STAT3 | 5.92E-05 | 11 |
| hsa04662 | B cell receptor signaling pathway | AKT1,FOS,JUN,MAPK1,NFKBIA,PIK3CG,RELA | 7.80E-05 | 7 |
| hsa05223 | Non-small cell lung cancer | AKT1,CCND1,ERBB2,MAPK1,PIK3CG,TP53 | 9.78E-05 | 6 |
| hsa04664 | Fc epsilon RI signaling pathway | AKT1,IL4,MAPK1,MAPK14,MAPK8,PIK3CG,TNF | 1.09E-04 | 7 |
| hsa04623 | Cytosolic DNA-sensing pathway | CXCL10,IL1B,IL6,NFKBIA,RELA,TBK1 | 1.20E-04 | 6 |
| hsa05330 | Allograft rejection | FAS,IFNG,IL2,IL4,TNF | 1.94E-04 | 5 |
| hsa05131 | Shigellosis | CXCL8,MAPK1,MAPK14,MAPK8,NFKBIA,RELA | 2.13E-04 | 6 |
| hsa04920 | Adipocytokine signaling pathway | AKT1,MAPK8,NFKBIA,RELA,STAT3,TNF | 3.54E-04 | 6 |
| hsa04912 | GnRH signaling pathway | JUN,MAPK1,MAPK14,MAPK7,MAPK8,MMP14,MMP2 | 5.04E-04 | 7 |
| hsa04370 | VEGF signaling pathway | AKT1,HSPB1,MAPK1,MAPK14,PIK3CG,PTGS2 | 6.47E-04 | 6 |
| hsa05014 | Amyotrophic lateral sclerosis (ALS) | BCL2,MAPK14,SOD1,TNF,TP53 | 9.03E-04 | 5 |
| hsa04350 | TGF-beta signaling pathway | IFNG,MAPK1,MYC,SP1,TGFB1,TNF | 1.17E-03 | 6 |
| hsa04670 | Leukocyte transendothelial migration | CTNNB1,MAPK14,MMP2,MMP9,NCF1,PIK3CG,VCAM1 | 1.21E-03 | 7 |
| hsa04914 | Progesterone-mediated oocyte maturation | AKT1,HSP90AA1,MAPK1,MAPK14,MAPK8,PIK3CG | 1.32E-03 | 6 |
| hsa04520 | Adherens junction | CTNNB1,ERBB2,FGFR1,MAPK1,MET | 3.48E-03 | 5 |
| hsa04930 | Type II diabetes mellitus | MAPK1,MAPK8,PIK3CG,TNF | 4.44E-03 | 4 |
| hsa04612 | Antigen processing and presentation | CREB1,HSP90AA1,HSPA5,IFNG,TNF | 4.63E-03 | 5 |
| hsa04672 | Intestinal immune network for IgA production | IL2,IL4,IL6,TGFB1 | 4.79E-03 | 4 |
| hsa04310 | Wnt signaling pathway | CCND1,CTNNB1,JUN,MAPK8,MMP7,MYC,TP53 | 5.14E-03 | 7 |
| hsa04150 | mTOR signaling pathway | AKT1,HIF1A,MAPK1,PIK3CG | 5.92E-03 | 4 |
| hsa04640 | Hematopoietic cell lineage | IL1A,IL1B,IL4,IL6,TNF | 7.71E-03 | 5 |
| hsa04110 | Cell cycle | CCND1,CDKN1A,MDM2,MYC,TGFB1,TP53 | 9.00E-03 | 6 |
| hsa04610 | Complement and coagulation cascades | F2,PLAU,SERPINE1,THBD | 1.58E-02 | 4 |
| hsa05100 | Bacterial invasion of epithelial cells | CAV1,CTNNB1,MET,PIK3CG | 1.74E-02 | 4 |
| hsa04650 | Natural killer cell mediated cytotoxicity | FAS,IFNG,MAPK1,PIK3CG,TNF | 4.21E-02 | 5 |
| hsa04666 | Fc gamma R-mediated phagocytosis | AKT1,MAPK1,NCF1,PIK3CG | 4.43E-02 | 4 |
| hsa04910 | Insulin signaling pathway | AKT1,FOXO1,MAPK1,MAPK8,/PIK3CG | 4.44E-02 | 5 |

**Figure S1**

**
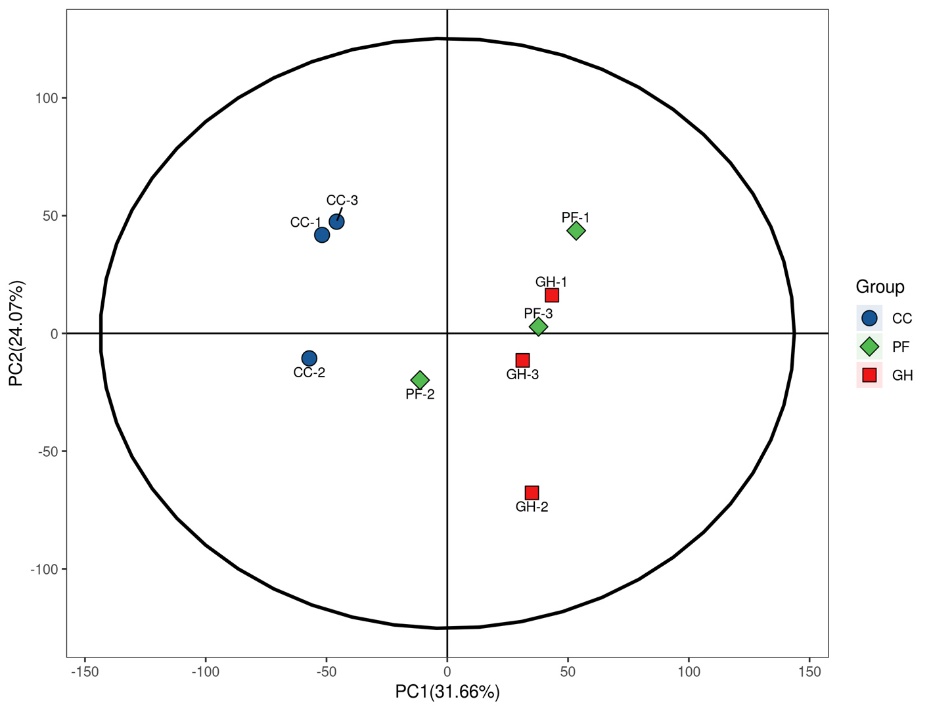
**

Figure S1 PCA plots for the proteomics of mice lung tissues.

**Figure S2**


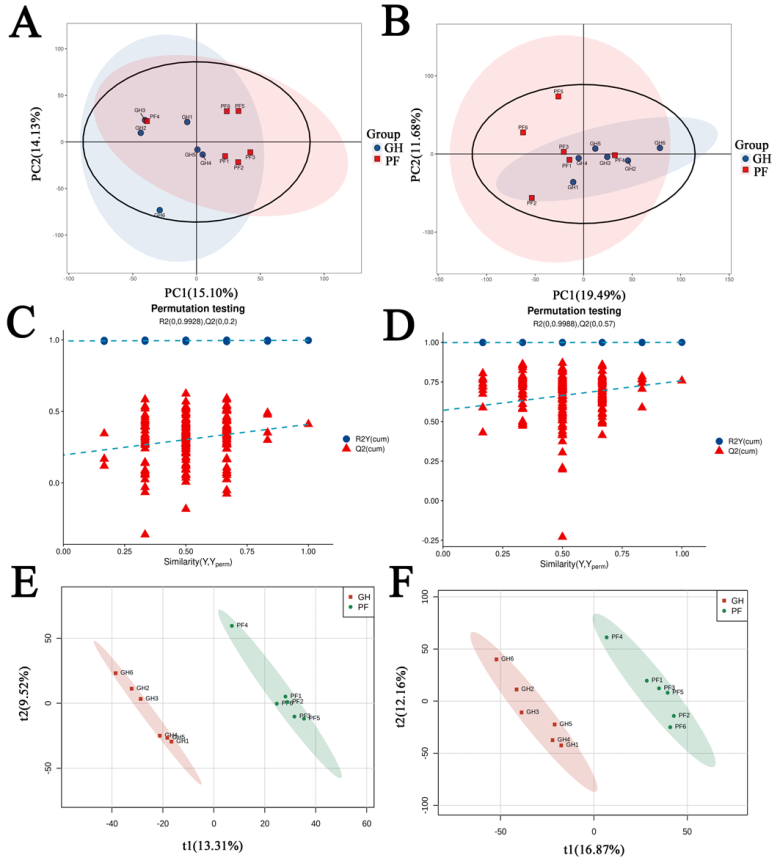


Figure S2 Metabolomic investigation of mice blood samples. (GH group, n=6; PF group, n=6).
